# Effectiveness of Robotic Devices for Medical Rehabilitation: An Umbrella Review

**DOI:** 10.3390/jcm13216616

**Published:** 2024-11-04

**Authors:** Kei Kiyono, Shigeo Tanabe, Satoshi Hirano, Takuma Ii, Yuki Nakagawa, Koki Tan, Eiichi Saitoh, Yohei Otaka

**Affiliations:** 1 Faculty of Rehabilitation, School of Health Sciences, Fujita Health University, Toyoake 470-1192, Aichi, Japan; kkiyono@fujita-hu.ac.jp (K.K.); tanabes@fujita-hu.ac.jp (S.T.); takuma@fujita-hu.ac.jp (T.I.); 2 Department of Rehabilitation Medicine, School of Medicine, Fujita Health University, Toyoake 470-1192, Aichi, Japan; sshirano@fujita-hu.ac.jp (S.H.); yuki.nakagawa@fujita-hu.ac.jp (Y.N.); koki.tan@fujita-hu.ac.jp (K.T.); esaitoh@fujita-hu.ac.jp (E.S.); 3 Graduate School of Health Sciences, Fujita Health University, Toyoake 470-1192, Aichi, Japan

**Keywords:** activities of daily living, electromechanical, physical therapy, occupational therapy, stroke, spinal cord injury, multiple sclerosis, cerebral palsy, Parkinson’s disease, brain injury

## Abstract

**Background/Objectives:** Clinical trials have investigated the efficacy of rehabilitation robotics for various pathological conditions, but the overall impact on rehabilitation practice remains unclear. We comprehensively examined and analyzed systematic reviews (SRs) of randomized controlled trials (RCTs) investigating rehabilitative interventions with robotic devices. **Methods:** Four databases were searched using term combinations of keywords related to robotic devices, rehabilitation, and SRs. The SR meta-analyses were categorized into “convincing”, “highly suggestive”, “suggestive”, “weak”, or “non-significant” depending on evidence strength and validity. **Results:** Overall, 62 SRs of 341 RCTs involving 14,522 participants were identified. Stroke was most frequently reported (40 SRs), followed by spinal cord injury (eight SRs), multiple sclerosis (four SRs), cerebral palsy (four SRs), Parkinson’s disease (three SRs), and neurological disease (any disease causing limited upper- and lower-limb functioning; three SRs). Furthermore, 38, 21, and 3 SRs focused on lower-limb devices, upper-limb devices, and both upper- and lower-limb devices, respectively. Quantitative synthesis of robotic intervention effects was performed by 51 of 62 SRs. Robot-assisted training was effective for various outcome measures per disease. Meta-analyses offering suggestive evidence were limited to studies on stroke. Upper-limb devices were effective for motor control and activities of daily living, and lower-limb devices for walking independence in stroke. **Conclusions:** Robotic devices are useful for improving impairments and disabilities in several diseases. Further high-quality SRs including RCTs with large sample sizes and meta-analyses of these RCTs, particularly on non-stroke-related diseases, are required. Further research should also ascertain which type of robotic device is the most effective for improving each specific impairment or disability.

## 1. Introduction

The number of people with physical impairments caused by diseases is increasing especially in countries with an aging population, leading to physical disabilities that substantially affect their activities of daily living (ADLs). It was recently reported that 2.41 billion individuals have conditions that would benefit from rehabilitation medicine [1]. In rehabilitation for physical impairments, an appropriate number of training programs that do not cause overuse and disuse should be provided to patients with optimal task difficulty in learning, repeated practice, and feedback. In addition to conventional physical and occupational therapy, interventions using technologies have been proposed to support the achievement of this essential goal. In particular, numerous novel robotic devices equipped with excellent features, such as high-precision sensing and accurate actuators without time delay, have been developed to establish better rehabilitation programs [2,3]. These robotic devices enable optimal assistance and load settings using feedback based on quantitative evaluation.

Many reviews concerned with the effect of robotic devices have been published for different diseases. Recent reviews have reported that various types of robotic devices for rehabilitative interventions could improve physical impairments and disabilities in patients with various diseases [4,5,6,7,8], and some clinical guidelines have suggested the use of robotic devices in the treatment of specific diseases [9,10]. Nevertheless, thus far, no review has provided a comprehensive overview of the cross-disease effectiveness of currently available robot-assisted training (RT). Through this umbrella review, we aimed to evaluate the comprehensive effectiveness of RT or robotic devices for medical rehabilitation, focusing on five areas: number of systematic reviews (SRs), randomized controlled trials (RCTs), and participants studied for each disease; quality of SRs for each disease; types of robotic devices used for each disease; outcome measures used in SRs for each disease; and effectiveness of RT for each outcome measure.

## 2. Materials and Methods

### 2.1. Study Design and Registration

The present study was designed as an umbrella review to comprehensively analyze SRs on the effectiveness of robotic devices for medical rehabilitation. The reporting of the key aspects of methods and results in this study adhered to the protocol for the Preferred Reporting Items for Overviews of Reviews (PRIOR) [11] guidelines. The review protocol was registered on the International Prospective Register of Systematic Reviews (PROSPERO; https://www.crd.york.ac.uk/prospero/, accessed on 10 June 2024) (registration number: CRD42024553070).

### 2.2. Criteria for Considering Studies for This SR

#### 2.2.1. Type of SRs

The present review considered SRs that assessed the effect of RT on upper- and lower-limb rehabilitation in patients with various symptoms and diseases and were published in English after 2009. SRs that met one of the following two criteria were included: (i) SRs of only RCTs with or without meta-analysis and (ii) SRs including at least one meta-analysis using only RCTs. The exclusion criteria were as follows: (i) SRs that did not include RCTs and (ii) SRs that did not perform a meta-analysis or that conducted a meta-analysis for only a mixture of both RCTs and non-RCTs in the case of SRs involving both RCTs and non-RCTs. (iii) SRs comparing effects through indirect comparison (network meta-analysis).

#### 2.2.2. Types of Participants

The present review considered participants with various symptoms and diseases but without any specific exclusion criteria, such as pathological conditions, age, and sex.

#### 2.2.3. Types of Interventions

The present review included interventions with robotic devices for rehabilitative treatment in patients with physical impairments and disabilities. Among various types of robotic devices developed for medical rehabilitation, robots involving interactive automation, sensors, and dynamic control mechanisms were included. However, (i) devices without features that deliver passive motion, such as treadmills, bicycles, and other simple mechanical trainers and (ii) a combination of robotics that utilized transcranial direct current stimulation and electromyography/electroencephalography during robotic gait training were excluded from analysis. The targeted robotic devices comprised different types of exoskeletons or end-effectors and unilateral or bilateral devices.

#### 2.2.4. Types of Outcome Measures

The present review included all physical function measures such as gait function and upper-limb function measures; ADL measures such as the Functional Independence Measure (FIM) and Barthel Index; and quality of life (QOL) measures.

### 2.3. Search Methods for the Identification of Studies

MEDLINE, EMBASE, Cochrane Database of Systematic Reviews, and PEDro were searched for studies published from 1 January 2009 to 31 December 2023 using term combinations of keywords (“rehabilitation” OR “physical therapy” OR “occupational therapy”) AND (“robotics” OR “robotic devices” OR “electromechanical” OR “robot-assisted”) AND (“systematic review” OR “meta-analysis”).

### 2.4. Data Collection Process and Analysis

#### 2.4.1. Selection of SRs

Two reviewers (K.K. and Y.O.) independently screened the titles and abstracts of all records. Any discrepancies between the reviewers were resolved through discussion or consultation with a third reviewer (S.T.) until consensus was achieved.

#### 2.4.2. Data Extraction and Management

Full texts were acquired, and two independent reviewers (K.K. and Y.O.) assessed them for inclusion. The first reviewer (K.K.) extracted the data from the SRs, and the second reviewer (Y.O.) subsequently checked the accuracy of these data. Errors were considered, and corrections were made. Disagreements regarding data extraction were resolved through discussion or by consulting a third reviewer (S.T.) until consensus was achieved.

#### 2.4.3. Managing Overlap of Primary Studies

A list of the primary studies included in each SR was assembled, and a matrix table was created to determine overlap between SRs. To avoid double counting outcome data, the number of RCTs, study participants, and robotic devices were calculated from RCTs that excluded overlap between SRs contained in the present review.

### 2.5. Analysis and Synthesis of Results

#### 2.5.1. Characteristics of Each SR

Data were extracted from each SR, and a table presenting characteristics such as participants, interventions, and comparators was created.

#### 2.5.2. Number of the Included SRs, RCTs, and Participants

The number of SRs, RCTs included in each SR, and participants enrolled in each RCT was examined with respect to diseases (stroke, spinal cord injury, etc.) and type of devices (upper- or lower-limb devices).

### 2.6. Quality Assessment of SRs

Two reviewers (K.K. and Y.O.) independently evaluated all SRs using the A MeaSurement Tool to Assess systematic Reviews 2 (AMSTAR 2) checklist [12], which contains 16 items such as the literature search procedure, data extraction, quality assessment, and statistical analyses of meta-analyses. In line with recommendations, Items 2, 4, 7, 9, 11, 13, and 15 were extracted as critical domains for AMSTAR 2 [12]. The overall score of AMSTAR 2 was used to rate the quality of each SR included in this review on the grade of 4 scale (high, moderate, low, or critically low) [12].

### 2.7. Robotic Devices Used in the Included SRs

The type of robots utilized was investigated according to diseases (stroke, spinal cord injury, etc.), and the number of robotic devices used was summarized.

### 2.8. Outcome Measures Used in the Included SRs

Five reviewers (K.K., T.I., Y.N., K.T., and Y.O.) discussed the reported outcome measures in each SR and categorized them according to the International Classification of Functioning, Disability and Health (ICF) using linking rules [13,14].

### 2.9. Grading the Evidence for the Effectiveness of RT

Depending on the strength and validity of evidence, meta-analyses of all SRs were categorized into “convincing”, “highly suggestive”, “suggestive”, “weak”, or “non-significant” [15]. “Convincing” meta-analyses were defined as meta-analyses that satisfied all of the following criteria: statistical significance at *p* < 10^−6^ per the random-effects model; based on >1000 participants; without large between-study heterogeneity (*I*^2^ < 50%); 95% prediction intervals excluding the null value; and no evidence of small-study effects and excess significance bias. “Highly suggestive” meta-analyses were defined as meta-analyses based on >1000 participants that included the largest study presenting a statistically significant effect at *p* < 10^−6^. “Suggestive” meta-analyses referred to meta-analyses based on >1000 participants with a significant effect at *p* < 10^−3^. The remaining nominally significant associations (*p* < 0.05) were considered to provide “weak” evidence. No statistically significant differences were classified as “non-significant”.

The largest number of participants in the meta-analyses corresponding to each disease and outcome measure category was extracted and summarized as the effectiveness of robot-assisted rehabilitation on various outcome measures in each disease. Stroke was further categorized as acute, chronic, and overall (no-specific phase) stroke. The phases from onset were classified into the following four categories according to each paper: (i) <3 months or ≤3 months; (ii) >3 months or ≥3 months; (iii) <6 months or ≤6 months; and (iv) >6 months or ≥6 months. Additionally, proximal and distal motor control was summarized as a subclassification of upper-limb motor control in stroke.

## 3. Results

**Figure 1:**
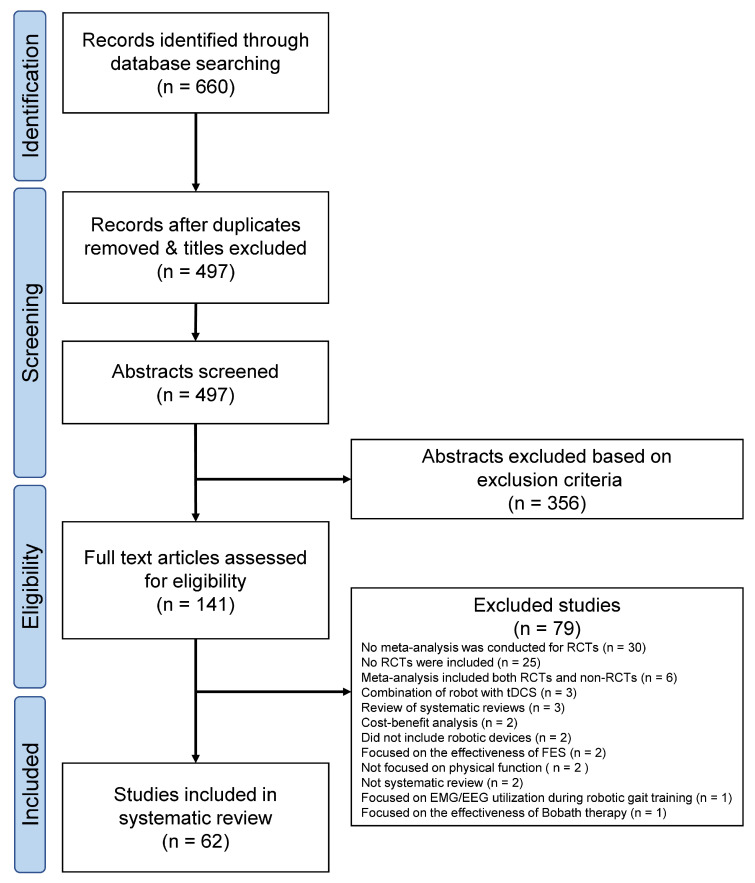
PRIOR flowchart. EEG, electroencephalography; EMG, electromyography; RCT, randomized controlled trials; tDCS, transcranial direct current stimulation.

Figure 1 presents the PRIOR flow diagram. The database search resulted in 660 abstracts. The two reviewers identified 141 articles by independent screening, for which the full texts were obtained. Independent screening of these 141 articles led to the selection of 62 SRs satisfying all criteria. Table S1 summarizes the characteristics of the included studies, whereas Table S2 lists the 79 excluded studies along with the reasons for exclusion.

### 3.1. Number of Included SRs, RCTs, and Participants

**Figure 2:**
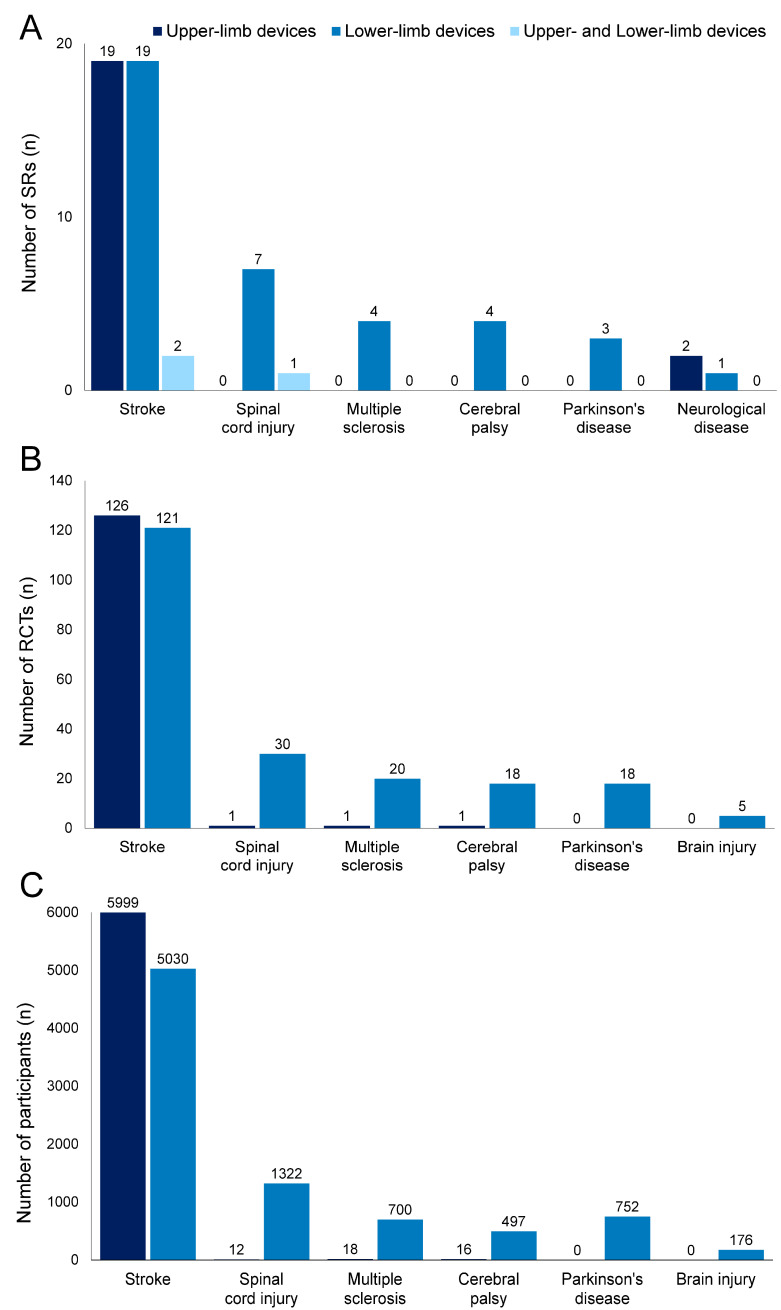
Number of systematic reviews (SRs), randomized controlled trials (RCTs), and participants according to diseases. Dark blue, upper-limb devices; blue, lower-limb devices; light blue, upper- and lower-limb devices. Number of SRs (**A**), RCTs (**B**), and participants (**C**). SRs on neurological disease included RCTs investigating stroke, cerebral palsy, and brain injury. Duplicates were excluded from RCTs (**B**) and corresponding participants (**C**).

Figure 2 shows the number of included SRs, RCTs, and participants (excluding overlaps in RCTs and their participants) according to diseases. Among the 62 SRs identified, stroke was the most frequently reported disease, with 40 SRs (upper-limb devices, 19 SRs [16,17,18,19,20,21,22,23,24,25,26,27,28,29,30,31,32,33,34]; lower-limb devices, 19 SRs [35,36,37,38,39,40,41,42,43,44,45,46,47,48,49,50,51,52,53]; upper- and lower-limb devices, 2 SR [54,55]), followed by spinal cord injury with 8 SRs (lower-limb devices, 7 SRs [56,57,58,59,60,61,62]; upper- and lower-limb devices, 1 SR [63]), multiple sclerosis with 4 SRs (lower-limb devices, 4 SRs [64,65,66,67]), cerebral palsy with 4 SRs (lower-limb devices, 4 SRs [68,69,70,71]), Parkinson’s disease with 3 SR (lower-limb devices, 3 SR [72,73,74]), and neurological disease (any disease causing limited upper-, and lower-limb functioning) with 3 SRs (upper-limb devices, 2 SRs [75,76]; lower-limb device, 1 SR [77]).

Table S3 (upper-limb devices) and Table S4 (lower-limb devices) show detailed information for all RCTs included in the SRs. The number of overlapping primary studies between SRs is extracted in Tables S3 and S4. The number of RCTs was 1110 and the number of participants was 48,099 before study overlap was removed. The most overlapping RCTs for upper and lower limb devices were used in 13 SRs and 12 SRs, respectively. After removing duplicates, 341 RCTs were included in SRs involving 14,522 participants. With respect to stroke, 126 RCTs (5999 participants) focused on upper-limb devices, whereas 121 RCTs (5030 participants) focused on lower-limb devices. Regarding spinal cord injury, one RCT (12 participants) focused on upper-limb devices, whereas 30 RCTs (1322 participants) focused on lower-limb devices. Regarding multiple sclerosis, one RCT (18 participants) focused on upper-limb devices, whereas 20 RCTs (700 participants) focused on lower-limb devices. Regarding cerebral palsy, one RCT (16 participants) focused on upper-limb devices, whereas 18 RCTs (497 participants) focused on lower-limb devices. Regarding Parkinson’s disease, 18 RCTs (752 participants) focused on lower-limb devices. SRs on neurological disease included RCTs investigating stroke, cerebral palsy, and brain injury. Among them, five RCTs on brain injury (176 participants) focused on lower-limb devices. 

### 3.2. Quality Assessment of SRs

Table S5 shows the methodological quality of the 62 included SRs, as assessed using AMSTAR 2. A total of 51 SRs [19,20,21,22,23,24,25,26,27,28,29,30,32,33,34,35,37,38,39,40,41,42,44,45,46,47,48,50,51,52,53,54,55,56,57,58,59,60,61,63,64,66,67,68,69,71,72,73,74,75,77] performed meta-analyses, whereas the remaining 11 SRs [16,17,18,20,31,43,49,62,65,70,76] attempted qualitative synthesis only. The quality of included reviews ranged from critically low to high. Of the 62 SRs, 3, 3, 7, and 49 SRs were classified to be of high, moderate, low, and critically low quality, respectively. For patients with stroke, 21 SRs conducted assessment of the included trials on upper-limb devices, and their quality ranged from critically low to high (AMSTAR 2 assessment: high for 1 SR, moderate for 1 SR, low for 4 SRs, and critically low for 15 SRs); 21 SRs also performed assessment of the included trials on lower-limb devices, and their quality ranged from critically low to high (AMSTAR 2 assessment: high for 2 SRs, moderate for 1 SR, low for 2 SRs, and critically low for 16 SRs). The reviews conducted by Saragih [54] and Lo [55] were counted as both upper- and lower-limb device studies. For patients with spinal cord injury, eight SRs conducted an assessment of the included trials on lower-limb devices, and their quality was critically low to moderate (AMSTAR 2 assessment: moderate for one SR, low for one SR, critically low for six SRs); one SR performed an assessment of the included trials on upper-limb devices, and its quality was also determined to be critically low based on the AMSTAR 2 assessment. The reviews conducted by Cheung [63] counted as both upper- and lower-limb device studies. For patients with multiple sclerosis, four SRs conducted assessment of the included trials on lower-limb devices, and their quality was critically low and moderate (AMSTAR 2 assessment: moderate for one SR, critically low for three SRs). With respect to other conditions, all SRs on upper- and lower-limb devices for cerebral palsy, Parkinson’s disease, and neurological disease achieved a critically low value based on AMSTAR 2 assessment.

Various critical flaws were identified in the reporting of the included SRs. Specifically, 87% of the SRs did not perform an adequate literature search (Q4), and 79% did not provide justification for excluding studies (Q7). Conversely, 94% of the included SRs reported the assessed risk of bias (Q9) and 84% of the included SRs used appropriate meta-analytical methods (Q11).

### 3.3. Robotic Devices Used in the Included SRs

**Figure 3:**
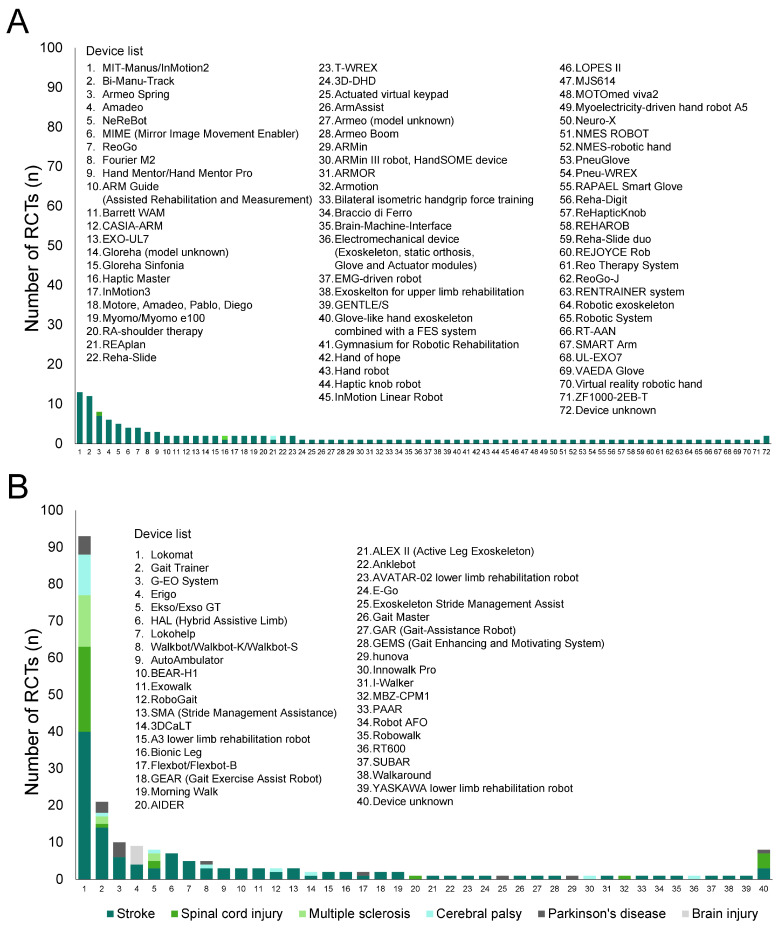
Number of robotic devices according to diseases. (**A**) Upper-limb devices; (**B**) lower-limb devices.

Figure 3 presents histograms of upper-limb (Figure 3A) and lower-limb (Figure 3B) robotic devices used in the RCTs included in the SRs (excluding overlaps). Detailed numbers are presented in Tables S6 and S7.

In the 129 analyzed studies, 71 types of upper-limb robotic devices, except for unknown devices, were used. All types were used in studies on patients with stroke, and three of the devices were also utilized in studies on patients with spinal cord injury, multiple sclerosis, and cerebral palsy. The most frequently used device was MIT-Manus/InMotion2 (13 RCTs), followed by Bi-Manu-Track (12 RCTs).

In the 212 analyzed studies, 39 types of lower-limb robotic devices, except for unknown devices, were used. Among the devices, 33 types were used in studies on stroke, whereas 5, 3, 8, 7, and 1 types were used in studies on spinal cord injury, multiple sclerosis, cerebral palsy, Parkinson’s disease and brain injury, respectively. The most frequently used device was Lokomat (93 RCTs) followed by Gait Trainer (21 RCTs).

### 3.4. Outcome Measures Used in the Included SRs

Table 1 and Table 2 show the outcome measures used in the included SRs for upper-limb and lower-limb devices, respectively. Outcome measures were categorized as “Body functions”, “Activities and participation”, and “Other measures” according to ICF. “Body functions” included motor control, muscle strength, muscle tone, range of motion, sensory function, pain, and fatigue. “Activities and participation” included upper-limb capacity, walking independence, walking speed, walking capacity, gait index, balance capacity, ADLs, and comprehensive measure. Lastly, “Other measures” included outcomes that fit into neither category of “Body functions” and “Activities and participation”.

Regarding upper-limb devices, studies on patients with stroke utilized various outcome measures on various aspects, ranging from body functions to QOL. In particular, 18 and 10 SRs performed meta-analyses with the Fugl-Meyer Assessment for Upper Extremity (FMA-UE) and the FIM, respectively; the Stroke Impact Scale was used in eight SRs for meta-analyses. In contrast, studies on patients with neurological disease used few outcome measures for meta-analysis. No outcome measures were used for meta-analyses of spinal cord injury.

As for lower-limb devices, studies on patients with stroke frequently used gait-related outcomes. Specifically, the Functional Ambulation Scale (FAC) was used in 12 SRs for meta-analysis, the 6-Minute Walk Test was used in 8 SRs for meta-analysis. Studies on patients with spinal cord injury, multiple sclerosis, cerebral palsy, and Parkinson’s disease used various aspect components; the outcome measures frequently used for meta-analysis were gait-related outcomes, notably walking speed and capacity. Table S8 presents the outcome measures used in the included studies in detail.

### 3.5. Effectiveness of RT

Table 3 and Table 4 show the effectiveness of RT and grading of evidence in various outcome measures for each disease. With respect to upper-limb devices, RT showed “suggestive” effectiveness for overall (including proximal and distal) motor control and ADLs in patients with stroke and “weak” effectiveness for proximal motor control, muscle strength, muscle tone, pain, and upper-limb capacity. Regarding the effectiveness by phases from onset, “weak” effectiveness was found for motor control, muscle tone, and ADLs in several phases from onset; however, there was no “suggestive” effectiveness. As for lower-limb devices, RT showed “suggestive” effectiveness for walking independence and “weak” effectiveness for motor control, walking speed, and balance capacity in patients with stroke (no specific phase). By phases from onset, effectiveness on walking independence was “suggestive” even in SRs limited to the acute phase, and “weak” effectiveness was found for gait index regardless of phases. RT exhibited “weak” effectiveness for muscle strength, walking independence, balance capacity, and ADLs in patients with spinal cord injury; muscle tone, fatigue, walking speed, walking capacity, and balance capacity in patients with multiple sclerosis; and walking speed, walking capacity, gait index, and other measures in patients with Parkinson’s disease. In patients with cerebral palsy, RT showed no significant differences compared with conventional therapy.

No “convincing” and “highly suggestive” grades of evidence were found in any meta-analyses among all SRs. Table S9 (upper-limb devices) and Table S10 (lower-limb devices) provide detailed data on all meta-analyses in SRs.

## 4. Discussion

In the present study, we thoroughly reviewed SRs on the effectiveness of robotic devices for medical rehabilitation and successfully elucidated the current quantity and quality of evidence, types of interventions and outcome measures used, and knowledge regarding the efficacy of robotic devices on various outcome measures in several diseases.

### 4.1. Number of the Included SRs, RCTs, and Participants

A substantially greater number of SRs focused on stroke than on other diseases (as shown in Figure 2). Furthermore, more SRs and RCTs focused on lower-limb devices than on upper-limb devices.

Two possible reasons could explain the limited number of SRs and RCTs for diseases other than stroke. First, the number of patients with stroke is predominantly higher than that of patients with other diseases and conditions. As of 2019, the global prevalence of stroke was estimated to be 86 million people, whereas those of spinal cord injury, Parkinson’s disease, and multiple sclerosis were 21 million, 3.9 million, and 1.4 million people, respectively [1]. Cost–benefit issues when attempting to develop robots or modify existing robots, such as size-down for pediatric use, may have also contributed to the poor availability of robotic devices and smaller amount of evidence in relatively fewer diseases. Second, the adaptability of robotic devices differs among pathological conditions. For instance, limb paresis in patients with stroke may be relatively easy to target when considering the application of robotic devices to aid them; thus, designing a robot may be easier. In contrast, narrowing down the target of RT and designing a robot are both difficult for patients with Parkinson’s disease, who present with bradykinesia and rigidity as systemic symptoms, and patients with multiple sclerosis, where the site of impairment varies between time and among individuals. This background may also be the reason for the lack of robotic applications.

### 4.2. Quality Assessment of SRs

Quality assessment of the reviews indicated that most SRs included for each disease were of critically low quality (49 SRs). SRs deemed to be of high methodological quality could only be found for patients with stroke. Hence, high-quality reviews on patients with other diseases or conditions are required, and the quality of each SR should be improved by disclosing the adequacy of the literature search for each RCT and justification for excluding studies.

### 4.3. Robotic Devices Used in the Included SRs

This study also explored the types of robotic devices used in the RCTs included in the SRs. A total of 71 upper-limb robotic devices and 39 lower-limb robotic devices were utilized in the trials (as shown in Figure 3). Compared with upper-limb devices, the types of lower-limb devices used were limited. Among lower-limb devices, Lokomat followed by Gait Trainer were investigated and used in most RCTs. Although the SRs included several RCTs, most presented evidence was from trials with a limited number of robots. Therefore, the results strongly reflect the effects of specific types of robots rather than the effects of robots in general. If multiple robots are combined in a study, the robots used in only a small number of RCTs may not be well represented, and the effects could be overshadowed. Sub-analyses of the types of robots used provide some ideas [25,33,41,66,68]; nonetheless, current studies have not clarified the optimal matching of pathological conditions and appropriate type of robotic devices, and more studies focusing on each device are needed to determine which robotic devices are effective against each disease.

### 4.4. Outcome Measures Used in the Included SRs

For upper-limb devices, the most frequently investigated outcome measure was the FMA-UE, which is related to ICF’s control of voluntary movement functions [b760]. Robotic rehabilitation could enhance motor control by providing patients with intensive and repetitive rehabilitation training and by consequently boosting the use-dependent plasticity process [78,79]. Considering that upper-limb devices are suitable for performing repetitive motion tasks, it is reasonable that outcome measures related to the control of voluntary movement functions have been often used in SRs.

For lower-limb devices, the most frequently investigated outcome measures were the FAC, which is related to walking independence of ICF’s walking [b450] domain. This result may be attributed to the fact that most of lower-limb robots are designed to assist in the walking independence of patients who are unable to walk. Lower-limb devices aim to improve walking performance, given that the main role of the lower limbs is mobility. Walking independence has often been set as a goal because it is important for medical rehabilitation.

### 4.5. Effectiveness of RT and Implications to Future Studies

Based on the included SRs, RT including upper and lower-limb devices was found to be effective for patients with stroke, spinal cord injury, multiple sclerosis, and Parkinson’s disease compared to conventional therapy or other interventions (as shown in Table 3 and Table 4). Specifically, the effectiveness of RT was rated as “suggestive” for upper-limb motor control and ADLs on patients with stroke (no specific phase) and for walking independence on patients with acute stroke. Evidence supporting the use of RT for patients with spinal cord injury, multiple sclerosis, cerebral palsy, and Parkinson’s disease was rated as “weak” for several outcomes. Thus, further studies, including RCTs with large sample sizes and meta-analyses of RCTs, should be conducted. This is especially important for diseases identified in the present study other than stroke and for diseases not identified in the study, such as musculoskeletal disorders. Additionally, because the effectiveness depending on severity and length from onset is still unclear, further studies should assess the effects of each robotic device while taking the disease severity and time after onset into account. By focusing on exploring the mechanisms underlying their effects helps to determine which type of robotic device is the most effective tool for improving each specific type of impairment or disability.

Additionally, the users’ acceptance of robotic devices and cost effectiveness of robot-assisted rehabilitation should be investigated. Some studies analyzed the cost–benefit of the use of robot-assisted rehabilitation [80,81]; however, evidence in the related area was limited. A comprehensive cost–benefit analysis that considers various scenarios for the use of robotic devices in actual clinical settings is essential for the widespread use of robotic rehabilitation.

### 4.6. Study Limitations

There were a few limitations in the study. Only studies in English were included in the present review, which might have led to an overestimation of the effectiveness of robotic devices because papers with positive results were possibly more likely to be published in English than in a local language. Nonetheless, we included studies from various regions worldwide, and a previous report confirmed that there was no evidence of a systematic bias from the use of language restrictions in SR-based meta-analyses in conventional medicine [82]. Thus, we believe that the lack of SRs written in non-English languages did not have a major impact. Another limitation is that this review did not include more recent reviews. Robotic rehabilitation is a fast-paced advancing field with respect to scientific publications and development speed, and newer reviews are therefore constantly required.

## 5. Conclusions

There were more types of upper-limb devices than of lower-limb devices, and in contrast, there were more RCTs and SRs on lower-limb devices than on upper-limb devices. There was suggestive evidence of robotic rehabilitation’s effectiveness for upper-limb motor control and walking independence in patients with stroke. Further high-quality SRs including RCTs with large sample sizes and meta-analyses of these RCTs, particularly on diseases other than stroke, are required.

## Figures and Tables

**Table 1:** Outcome measures used in the included studies on upper-limb devices.

	Body Functions						Activities and Participation			Other Measures
	Motor Control	Muscle Strength	Muscle Tone	Range of Motion	Sensory Function	Pain	Upper-Limb Capacity	Activities of Daily Living	Comprehensive Measures	
	Control of Voluntary Movement Functions [b760]	Muscle Power Functions [b730]	Muscle Tone Functions [b735]	Mobility of Joint Functions [b710]	Touch Function [b265]	Sensation of Pain [b280]	Fine Hand Use [d440], Hand and Arm Use [d445]	Washing Oneself [d510], Toileting [d520], Dressing [d540], Eating [d550]		
Stroke	FMA-UE (18) CMSA (3) FMA-WH (2) MSS (2) FMA-SE (1) K-SDQ	MI (6) ^a^ MRC (4) MPS (3) Grip force (1) Grip strength (1) Maximum resistive force (1) MMT (1) Motor power range (1) Dynamometer Surface EMG	MAS (5) AS (1)	pROM (1) ROM	Revised Nottingham Sensation Assessment Semmes–Weinstein hand monofilament test	VAS (2) CMSA Pain Inventory Scale (1) Pain Scale of FM (1) California functional evaluation (1) DN4 (1) Pain scale (1) NRS The severity degree of the painful shoulder was defined in four grades	WMFT (5) ARAT (4) QuickDASH (3) FAT (3) MAL (3) ABILHAND (2) AMAT (2) BBT (2) CAHAI (1) NHPT MAL-QOM Reaching Performance Scale Shoulder/Elbow Coordination Index	FIM (10) BI (7) MBI (4) mRS (2) ACTIVLIM questionnaire (1) Korean MBI (1)	SIS (8) ^a^ SF-36 (1) MCS PCS	Addenbrooke cognitive examination—revised (1) Dropouts during the intervention period (1) MMSE (1) participants’ cognitive function (1) Goal Attainment scale
Spinal cord injury	GRASSP						ARAT			
Neurological disease	FMA-UE Jebsen–Taylor Hand Function Test	MI ^a^						FIM	Life Habits (1) SIS (1) SF-36	QUEST UPDRS

Numbers within brackets [] indicate the categories of International Classification of Functioning, Disability and Health (ICF). Values within parentheses () indicate the number of systematic reviews that used the measure in a meta-analysis. ^a^ The outcome measure that might include more than one ICF code was classified according to the intent of the paper. Abbreviations: ABILHAND, A Measure of Manual Ability for People with Upper Limb Impairment; ACTIVLIM, Activity Limitations for Patients with Upper and/or Lower Limb Impairments; AMAT, Arm Motor Ability Test; ARAT, Action Research Arm Test; AS, Ashworth Scale; BBT, Box and Block Test; BI, Barthel Index; CAHAI, Chedoke Arm and Hand Activity Inventory; CMSA, Chedoke–McMaster Stroke Assessment; EMG, electromyography; FIM, Functional Independence Measure; FMA, Fugl–Meyer Assessment; FMA-SE, Fugl–Meyer Assessment-Shoulder and Elbow; FMA-UE, Fugl–Meyer Assessment-Upper Extremity; FMA-WH, Fugl–Meyer Assessment-Wrist and Hand; FAT, Frenchay Arm Test; GRASSP, Graded Redefined Assessment of Strength, Sensibility and Prehension; K-SDQ, Korean version of the Shoulder Disability Questionnaire; MAL, Motor Activity Log; MAL-QOM, the Quality of 124 Movement Section of the Motor Activity Log; MAS, Modified Ashworth Scale; MBI, Modified Barthel Index; MCS, Mental Composite Score; MI, Motricity Index; MMT, Manual Muscle Testing; MPS, Motor Power Scale; MRC, Medical Research Council; mRS, modified Rankin Scale; MSS, Motor Status Scale; NHPT, Nine-Hole Peg Test; NRS, Numerical Rating Scale; PCS, Physical Composite Score; QUEST, Quality of Upper Extremity Skills Test; QuickDASH, Quick version of the Disabilities of the Arm, Shoulder, and Hand questionnaire; pROM, Passive Range of Motion; SF-36, Short-Form 36; SIS, Stroke Impact Scale; UPDRS, Unified Parkinson’s Disease Rating Scale; VAS, Visual Analog Scale; WMFT, Wolf Motor Function Test.

**Table 2:** Outcome measures used in the included studies on lower-limb devices.

	Body Functions					Activities and Participation							Other Measures
	Motor Control	Muscle Strength	Muscle Tone	Pain	Fatigue	Walking Independence	Walking Speed	Walking Capacity	Gait Index	Balance Capacity	Activities of Daily Living	Comprehensive Measures	
	Control of Voluntary Movement Functions [b760]	Muscle Power Functions [b730]	Muscle Tone Functions [b735]	Sensation of Pain [b280]	Fatiguability [b4552]	Walking [d450]				Changing and Maintaining Body Position [d410–d429]	Washing Oneself [d510], Toileting [d520], Dressing [d540], Eating [d550]		
Stroke	CMSR (1) FMA-B (1) FMA-LE (1) MI (1) ^a^					FAC (12) 15 m continuously with no aid (1) FGA	10MWT (6) Walking speed (4) 5MWT (4) Maximum walking speed	6MWT (8) RMI (6) 2MWT (4) EMS (1) Endurance (1) EU walking (1) 5MWT (1) ^a^ MMAS (1) Motor Assessment Scale (1) Peak VO_2_ (1) RMA (1) VO_2_ during the 5-Minute Walk Test (1) mEFAP MM WHS	Cadence (2) Spatial symmetry (1) Step length (1) Stride length (1) Temporal symmetry (1) Consistency of intralimb movements on the impaired limb Number of steps Rivermead Gait Assessment Stance duration and single-support time of both legs Stance time Stride duration and cadence Walking distance	BBS (8) TUG (6) Brunel Balance Assessment (1) PASS (1) ABC Dynamic balance time Dynamic balance trip Functional reach POMA-B SPPB Standing forward reach test Static balance test Sit-to-stand testTrunk impairment scale TWT	FIM (6) BI (5) ADL-IADL (1) FAI (1) MeEAP (1) SAS (1) SIS (1) ^a^	QOL	Death from all causes until the end of the intervention phase (2) Dropouts (2) Lost to study during the intervention phase (2) Acceptability Mood Safety
Spinal cord injury	GRASSP	LEMS (6) AMI MRC	MAS (2) AS (1) Intrinsic stiffness (1) Reflex stiffness (1) Intrinsic & reflex stiffness SCATS	VAS (1) PGIC		WISCI II (3) WISCI (2)	10MWT (5) 15MWT (1) Walking speed	6MWT (5) 2MWT (2) Peak VO_2_ (1) Walking distance (1) AT FEV1 FVC Max VO_2_ Maximal voluntary ventilation Maximum heart rate Maximum oxygen consumption during a functional task Maximum oxygen consumption during a nonfunctional task Metabolic equivalent of energy PEF	Ankle kinematic and kinetic assessments Gait characteristics	TUG (1) BBS MFR	FIM-locomotor (2) SCIM III (1) MBI SCIM		
Multiple sclerosis			MAS (2) VAS (1) ^a^	VAS (2) ^a^ “Bodily pain” from SF-36 (1) Medical Outcomes Study Pain Effects Scale (1)	Fatigue Severity Scale (2) Cognitive and physical fatigue score (1) Modified fatigue impact scale (1), Würzburger Erschöpfungsinventar bei Multipler Sklerose scale (1)	FAC (1)	10MWT (3) 20MWT (3) T25FW (3) Laboratory measures for walking speed evaluation (1) Walking speed (1) 3MWS	2MWT (3) 6MWT (3) 3MWT (2) RMI (2)	Cadence (1) Double support time (1) Stride length (1) Step length	BBS (2) TUG (2) Tinetti Test (1) ABC SOT	FIM (2) BI (1) MBI (1)	MSQOL-54 (2) RAND-36 (2) SF-36 (2)	EDSS (2) Treatment acceptance: VAS (1)
Cerebral palsy		Muscle strength				FAQ-WL (1) FAC	10MWT (3) 3D gait (1) Cadence (1) Free walking speed (1) Step length (1) Step width (1) Stride length (1) Walking speed	6MWT (2) Peripheral O_2_ saturation Walking ability	Gait parameters Gait patterns Lower-limb kinematics	Balance Postural and locomotor functions Standing activity Upper-body control	WeeFIM (1) COPM Functional independence Running and climbing activities		GMFM-D (2) GMFM-E (2)
Parkinson’s disease							10MWT (3) Walking speed (2)	6MWT (2)	Cadence (3) Stride length (3) Step length (1) Time (1)	BBS (3) TUG (3) ABC (2)			UPDRS-III (3)
Neurological disease	FM	MRC (1) Dynamometry	MAS (1)							Tinetti test	FIM		Blood pressure Coma Recovery Scale-Revised Heart rate Oxygen saturation

Numbers within brackets [] indicate the categories of International Classification of Functioning, Disability and Health (ICF). Values within parentheses () indicate the number of systematic reviews that used the measure in a meta-analysis. ^a^ The outcome measure that might include more than one ICF code was classified according to the intent of the paper. Abbreviations: 10MWT, 10-Meter Walk Test; 15MWT, 15-Meter Walk Test; 20MWT, 20-Meter Walk Test; 2MWT, 2-Minute Walk Test; 3D, Three-Dimensional; 3MWS, 3-Minute Walking Speed; 3MWT, 3-Minute Walk Test; 5MWT, 5-Meter Walk Test; 6MWT, 6-Minute Walk Test; ABC, Activities-specific Balance Confidence scale; ADL, Activities of Daily Living; AMI, Ambulatory Motor Index; AS, Ashworth Scale; AT, Anaerobic Threshold; BBS, Berg Balance Scale; BI, Barthel Index; CMSR, Chedoke–McMaster Stages of Recovery; COPM, Canadian Occupational Performance Measure; EDSS, Expanded Disability Status Scale; EMS, Elderly Mobility Scale; FAC, Functional Ambulation Scale; FAI, Frenchay Activities Index; FEV1; Forced Expiratory Volume in the First 1 Second; FVC, Forced Vital Capacity; FGA, Functional Gait Assessment; FIM, Functional Independence Measure; FMA-B, Fugl–Meyer Assessment-Balance; FMA-LE, Fugl–Meyer Assessment-Lower Extremity; GMFM, Gross Motor Function Measure; GRASSP, Graded Redefined Assessment of Strength, Sensibility and Prehension; IADL, Instrumental Activities of Daily Living; LEMS, Lower Extremity Motor Score; MAS, Modified Ashworth Scale; MeEAP, Measure of Experiential Aspects of Participation; mEFAP, Modified Emory Functional Ambulation Profile; MFR, Modified Functional Reach; MI, Motricity Index; MM, Mobility Milestones; MMAS, Modified Motor Assessment Scale; MRC, Medical Research Council; MSQOL-54, Multiple Sclerosis Quality of Life-54; PASS, Postural Assessment Scale for Stroke; PEF, Peak Expiratory Flow; PGIC, Patient Global Impression of Change; POMA-B, Performance-Oriented Mobility Assessment-Balance; RAND-36, RAND 36-Item Health Survey; RMA, Rivermead Motor Assessment; RMI, Rivermead Mobility Index; SAS, Stroke Activity Scale; SCATS, Spinal Cord Assessment Tools for Spastic reflexes; SCIM, Spinal Cord Independence Measure; SF-36, Short-Form 36; SIS, Stroke Impact Scale; SOT, Sensory Organization Balance Test; SPPB, Short Physical Performance Battery; T25FW, Timed 25-Foot Walk; TUG, Timed Up and Go; TWT, Timed Walk Test; UPDRS, Unified Parkinson’s Disease Rating Scale; VAS, Visual Analog Scale; WHS, Walking Handicap Scale; WISCI, Walking Index for Spinal Cord Injury.

**Table 3:** Effectiveness of robot-assisted training with upper-limb devices.

	Body Functions							Activities and Participation			Other Measures
	Motor Control			Muscle Strength	Muscle Tone	Range of Motion	Pain	Upper-Limb Capacity	Activities of Daily Living	Comprehensive Measures	
	Control of Voluntary Movement Functions [b760]			Muscle Power Functions [b730]	Muscle Tone Functions [b735]	Mobility of Joint Functions [b710]	Sensation of Pain [b280]	Fine Hand Use [d440], Hand and Arm Use [d445]	Washing Oneself [d510], Toileting [d520], Dressing [d540], Eating [d550]		
	Overall	Proximal	Distal								
Stroke no specific phase	1. FMA-UE	1. FMA-SEC score	1. FMA-WH score	1. Grip force, MI, MRC	1. MAS	-	1. California functional evaluation, Douleur Neuropathique pain scale, pain scale, VAS	1. AMAT, ARAT, BBT, CAHAI, NHPT, WMFT	1. ABILHAND, BI, FIM, MBI, SIS	1. SF-36, SIS	1. Dropouts during the intervention period
	2. n = 2305	2. n = 369	2. n = 443	2. n = 826	2. n = 485		2. n = 261	2. n = 1557	2. n = 1768	2. n = 849	2. n = 1619
	3. 0.20 [0.09 0.32]	3. 2.62 [1.48 3.76]	3. 1.22 [−0.61 3.05]	3. 0.46 [0.16 0.77]	3. −1.03 [−2.06 0.01]		3. −0.34 [−0.58 −0.09]	3. 0.109 [−0.066 0.284]	3. 0.30 [0.14 0.45]	3. −0.06 [−0.20 0.08]	3. 0.00 [−0.02 0.02]
	4. *p* = 0.0007	4. *p* < 0.00001	4. *p* = 0.19	4. *p* = 0.0032	4. *p* < 0.00001		4. *p* = 0.01	4. *p* = 0.02	4. *p* = 0.0002	4. *p* = 0.378	4. *p* = 0.93
	5. 37%	5. 34%	5. 75%	5. 76%	5. 96%		5. 0%	5. 56.4%	5. 53%	5. 35.6%	5. 0%
	6. Zhang (2022) [22]	6. Veerbeek (2017) [32]	6. Veerbeek (2017) [32]	6. Mehrholz (2018) [29]	6. Yang (2023) [20]		6. Saragih (2023) [54]	6. Chen (2020) [26]	6. Zhang (2022) [22]	6. Chen (2020) [26]	6. Mehrholz (2018) [29]
Stroke < 3 months	1. FMA-UE	1. FMA-SEC score	1. FMA-WH score	1. MI (Arm subscale), MPS, MRC	1. AS, MAS	-	-	1. AMAT, ARAT, BBT, WMFT	1. ABILHAND, BI, FIM, Frenchay Arm Test, MBI, SIS 2.0, SIS 3.0 (motor function, social participation)	-	-
	2. n = 650	2. n = 251	2. n = 251	2. n = 346	2. n = 299			2. n = 195	2. n = 532		
	3. −0.11 [−2.38 2.16]	3. 2.81 [1.44 4.17]	3. 2.53 [0.46 4.60]	3. 0.21 [−0.23 0.64]	3. 0.21 [−0.03 0.45]			3. −0.00 [−0.29 0.29]	3. 0.40 [0.10 0.70]		
	4. *p* = 0.93	4. *p* < 0.0001	4. *p* = 0.02	4. *p* = 0.35	4. *p* = 0.08			4. *p* = 0.99	4. *p* = 0.0085		
	5. 0.21%	5. 44%	5. 85%	5. 69%	5. 47%			5. 40%	5. 63%		
	6. Saragih (2023) [54]	6. Veerbeek (2017) [32]	6. Veerbeek (2017) [32]	6. Veerbeek (2017) [32]	6. Veerbeek (2017) [32]			6. Veerbeek (2017) [32]	6. Mehrholz (2018) [29]		
Stroke > 3 months	1. FMA-UE	1. FMA-SEC score	1. FMA-WH score	1. MI (Arm subscale), MPS, MRC	1. MAS	-	-	1. AMAT, ARAT, BBT, WMFT	1. ABILHAND, BI, FIM, Frenchay Arm Test, MBI, SIS 2.0, SIS 3.0 (motor function, social participation)	-	-
	2. n = 901	2. n = 118	2. n = 192	2. n = 148	2. n = 276			2. n = 487	2. n = 425		
	3. 0.68 [0.15 1.21]	3. 2.17 [0.09 4.25]	3. −0.19 [−1.65 1.27]	3. −0.04 [−0.37 0.29]	3. −1.89 [−3.33 −0.44]			3. 0.05 [−0.13 0.23]	3. 0.19 [−0.13 0.50]		
	4. *p* = 0.01	4. *p* = 0.04	4. *p* = 0.80	4. *p* = 0.82	4. *p* = 0.01			4. *p* = 0.58	4. *p* = 0.24		
	5. 90%	5. 23%	5. 27%	5. 0%	5. 94%			5. 0%	5. 54%		
	6. Yang (2023) [20]	6. Veerbeek (2017) [32]	6. Veerbeek (2017) [32]	6. Veerbeek (2017) [32]	6. Yang (2023) [20]			6. Veerbeek (2017) [32]	6. Mehrholz (2018) [29]		
Stroke < 6 months	1. FMA-UE	-	-	1. Grip strength, maximum resistive force with WAM control program, MI, MMT, motor power range, MPS, MRC	1. MAS	1. pROM with the assistance of WAM or therapist for the elbow, total pROM	1. CMSA Pain Inventory Scale range 1–7, Pain Scale of FM, VAS	-	1. FIM	1. SIS	-
	2. n = 518			2. n = unknown	2. n = 135	2. n = 53	2. n = 19		2. n = 237	2. n = 149	
	3. 0.17 [−0.08 0.42]			3. 0.0 [−0.9 1.0]	3. −0.04 [−0.38 0.30]	3. 0.2 [−0.4 0.7]	3. 0.3 [−0.6 1.2]		3. 0.26 [0.05 0.47]	3. 0.03 [−0.30 0.36]	
	4. *p* = 0.177			4. *p* = 0.967	4. *p* = 0.81	4. *p* = 0.491	4. *p* = 0.565		4. *p* = 0.7	4. *p* = 0.86	
	5. 53.2%			5. 27.02%	5. 0%	5. -%	5. -%		5. 81.3%	5. 0%	
	6. Wu (2021) [25]			6. Ferreira (2018) [28]	6. Chien (2020) [27]	6. Ferreira (2018) [28]	6. Ferreira (2018) [28]		6. Bertani (2017) [30]	6. Chien (2020) [27]	
Stroke > 6 months	1. FMA-UE	-	-	1. Grip strength, maximum resistive force with WAM control program, MI, MMT, motor power range, MPS, MRC	1. MAS	1. pROM with the assistance of WAM or therapist for the elbow, total pROM	1. CMSA Pain Inventory Scale range 1–7, Pain Scale of FM, VAS	-	1. FIM	-	-
	2. n = 826			2. n = unknown	2. n = unknown	2. n = unknown	2. n = unknown		2. n = 339		
	3. 0.26 1 [0.12 0.41]			3. 1.2 [−0.0 2.3]	3. −0.8 [−2.5 0.9]	3. −0.4 [−0.9 0.2]	3. −0.2 [−0.6 0.2]		3. 0.26 [0.05 0.47]		
	4. *p* < 0.001			4. *p* = 0.051	4. *p* = 0.36	4. *p* = 0.212	4. *p* = 0.264		4. *p* = 0.01		
	5. 2.3%			5. 16.51%	5. 0%	5. 0%	5. 0%		5. 0%		
	6. Wu (2021) [25]			6. Ferreira (2018) [28]	6. Ferreira (2018) [28]	6. Ferreira (2018) [28]	6. Ferreira (2018) [28]		6. Bertani (2017) [30]		
Neurological disease	-	-	-	-	-	-	-	-	-	1. Life Habits, SIS	-
										2. n = 492	
										3. −0.60 [−1.10 −0.10]	
										4. *p* = 0.022	
										5. 6.99%	
										6. Ferreira (2021) [75]	

1. Outcome measure, 2. number of participants, 3. effect size [95% confidence interval], 4. *p* value, 5. heterogeneity; I^2^, 6. systematic review. The color coding in the table pertains to the following: Deep green = Suggestive; Light green = Weak; Red = Nonsignificant. Abbreviations: ABILHAND, A Measure of Manual Ability for People with Upper Limb Impairment; AMAT, Arm Motor Ability Test; ARAT, Action Research Arm Test; AS, Ashworth Scale; BBT, Box and Block Test; BI, Barthel Index; CAHAI, Chedoke Arm and Hand Activity Inventory; CMSA, Chedoke–McMaster Stroke Assessment; FIM, Functional Independence Measure; FM, Fugl–Meyer; FMA-SEC, Fugl–Meyer Assessment-Shoulder/Elbow and Coordination; FMA-UE, Fugl–Meyer Assessment-Upper Extremity; FMA-WH, Fugl–Meyer Assessment-Wrist and Hand; MAS, Modified Ashworth Scale; MBI, Modified Barthel Index; MI, Motricity Index; MMT, Manual Muscle Testing; MPS, Motor Power Scale; MRC, Medical Research Council; NHPT, Nine-Hole Peg Test; pROM, passive range of motion; SF-36, Short-Form 36; SIS, Stroke Impact Scale; VAS, Visual Analog Scale; WMFT, Wolf Motor Function Test.

**Table 4:** Effectiveness of robot-assisted gait training with lower-limb devices.

	Body Functions					Activities and Participation							Other Measures
	Motor Control	Muscle Strength	Muscle Tone	Pain	Fatigue	Walking Independence	Walking Speed	Walking Capacity	Gait Index	Balance Capacity	Activities of Daily Living	Comprehensive Measures	
	Control of Voluntary Movement Functions [b760]	Muscle Power Functions [b730]	Muscle Tone Functions [b735]	Sensation of Pain [b280]	Fatiguability [b4552]	Walking [d450]				Changing and Maintaining Body Position [d410–d429]	Washing Oneself [d510], Toileting [d520], Dressing [d540], Eating [d550]		
Stroke no specific phase	1. FMA-B	-	-	-	-	1. FAC, FIM, RMI	1. Walking speed	1. 6MWT	1. Cadence	1. BBS	-	-	1. Death
	2. n = 180					2. n = 1567	2. n = 1600	2. n = 983	2. n = 349	2. n = 929			2. n = 2440
	3. 3.57 [2.81 4.34]					3. 2.14 [1.57 2.92]	3. 0.06 [0.02 0.10]	3. 10.86 [−5.72 27.44]	3. 1.44 [−2.34 5.22]	3. 4.64 [3.22 6.06]			3. 0.00 [−0.01 0.01]
	4. *p* < 0.00001					4. *p* < 0.00001	4. *p* = 0.004	4. *p* = 0.20	4. *p* = 0.46	4. *p* < 0.00001			4. *p* = 0.82
	5. 0%					5. 6%	5. 60%	5. 42%	5. 92%	5. >50%			5. 0%
	6. Zheng (2019) [47]					6. Mehrholz (2020) [44]	6. Mehrholz (2020) [44]	6. Mehrholz (2020) [44]	6. Nedergard (2021) [40]	6. Zheng (2019) [47]			6. Mehrholz (2020) [44]
Stroke < 3 months	-	-	-	-	-	1. FAC, FIM, RMI	1. 10MWT	1. 6MWT	1. Cadence	-	-	-	-
						2. n = 1243	2. n = 142	2. n = 88	2. n = 93				
						3. 1.96 [1.47 2.62]	3. 0.10 [−0.00 0.21]	3. 35.46 [−12.98 83.91]	3. −6.47 [−10.18 −2.76]				
						4. *p* < 0.00001	4. *p* =0.058	4. *p* = 0.1514	4. *p* = 0.0006				
						5. 0%	5. 0.01%	5. 0.1%	5. 63%				
						6. Mehrholz (2020) [44]	6. Ada (2010) [53]	6. Ada (2010) [53]	6. Zhu (2023) [37]				
Stroke > 3 months	-	-	-	-	-	1. FAC, FIM, RMI	-	-	-	-	-	-	-
						2. n = 461							
						3. 1.20 [0.40 3.65]							
						4. *p* = 0.74							
						5. 29%							
						6. Mehrholz (2020) [44]							
Stroke < 6 months	1. CMSR, FMA-LE, MI	-	-	-	-	-	1. 10MWT, 2MWT, 5MWT	1. 2MWT, 6MWT, 5-Minute Walking Test, peak VO_2_	-	1. BBS, Brunel Balance Assessment, PASS, TUG	1. ADL-IADL, FAI, FIM, SAS, SIS	-	-
	2. n = 389						2. n = 487	2. n = 471		2. n = 223	2. n = 586		
	3. 0.15 [−0.09 0.40]						3. 0.01 [−0.08 0.09]	3. −0.04 [−0.36 0.28]		3. 0.20 [−0.40 0.80]	3. 0.14 [−0.13 0.42]		
	4. *p* = 0.22						4. *p* = 0.89	4. *p* = 0.82		4. *p* = 0.51	4. *p* = 0.30		
	5. 27%						5. 66%	5. 66%		5. 79%	5. 60%		
	6. Hsu (2020) [42]						6. Hsu (2020) [42]	6. Hsu (2020) [42]		6. Hsu (2020) [42]	6. Hsu (2020) [42]		
Stroke > 6 months	-	-	-	-	-	-	1. 10MWT, 5MWT, 6MWT, TUG	-	1. Cadence	1. BBS	-	-	-
							2. n = 130		2. n = 37	2. n = 129			
							3. −0.05 [−0.44 0.34]		3. 2.97 [1.35 4.59]	3. 1.61 [−0.02 3.35]			
							4. *p* > 0.05		4. *p* = 0.0004	4. *p* = 0.05			
							5. -%		5. 0%	5. 0%			
							6. Bruni (2018) [48]		6. Zhu (2023) [37]	6. Wang (2021) [41]			
Spinal cord injury	-	1. LEMS	1. MAS	1. VAS	-	1. WISCI, WISCI-II	1. 10MWT	1. 6MWT	-	1. TUG	1. FIM-Locomotion, WISCI-II	-	-
		2. n = 408	2. n = 110	2. n = 93		2. n = 122	2. n = 357	2. n = 236		2. n = 120	2. n = 250		
		3. 0.81 [0.14 1.48]	3. 0.51 [−0.00 1.02]	3. −0.890 [−3.086 1.306]		3. −3.73 [−4.92 −2.53]	3. 0.02 [−0.02 0.06]	3. 16.05 [−15.73 47.83]		3. 9.25 [2.76 15.73]	3. 0.40 [0.02. 0.78]		
		4. *p* < 0.0000	4. *p* = 0.05	4. *p* = 0.427		4. *p* < 0.00001	4. *p* = 0.25	4. *p* = 0.32		4. *p* = 0.005	4. *p* = 0.04		
		5. 84.5%	5. 62%	5. 75%		5. 38%	5. 40%	5. 69%		5. 74%	5. 47%		
		6. Wan (2024) [57]	6. Li (2023) [58]	6. Fang (2020) [59]		6. Cheung (2017) [63]	6. Nam (2017) [60]	6. Nam (2017) [60]		6. Nam (2017) ^a^ [60]	6. Nam (2017) [60]		
Multiple sclerosis	-	-	1. MAS, VAS	1. Bodily pain on SF-36, VAS	1. Fatigue severity scale, Modified fatigue imapact scale, Würzburger Erschöpfungsinventar bei Multipler Sklerose scale	1. FAC, TUG	1. 10MWT, 20MWT, T25FW, gait speed	1. 6MWT	1. Stride strength	1. BBS, Tinetti. test	1. FIM, MBI	1. MSQOL-54,36-item short-form health survey, RAND-36	1. EDSS
			2. n = 92	2. n = 165	2. n = 307	2. n = 203	2. n = 342	2. n = 414	2. n = 67	2. n = 325	2. n = 139	2. n = 234	2. n = 144
			3. 0.70 [0.08 1.33]	3. 0.10 [−0.21 0.40]	3. −0.27 [−0.49 −0.04]	3. 0.15 [−0.37 0.67]	3. 0.38 [0.15 0.60]	3. 0.26 [0.04 0.48]	3. 0.30 [−0.18 0.79]	3. 0.26 [0.04 0.48]	3. −0.02 [−0.36 0.32]	3. 0.25 [−0.01 0.51]	3. −0.25 [−0.58 0.08]
			4. *p* = 0.03	4. *p* = 0.53	4. *p* = 0.02	4. *p* = 0.58	4. *p* = 0.0010	4. *p* = 0.02	4. *p* = 0.22	4. *p* = 0.02	4. *p* = 0.91	4. *p* = 0.06	4. *p* = 0.14
			5. 53%	5. 0%	5. 0%	5. 68%	5. 6%	5. 18%	5. 0%	5. 0%	5. 0%	5. 0%	5. 0%
			6. Yeh (2020) [66]	6. Yeh (2020) [66]	6. Yang (2023) [64]	6. Yeh (2020) [66]	6. Yang (2023) [64]	6. Yang (2023) [64]	6. Yeh (2020) [66]	6. Yang (2023) [64]	6. Yang (2023) [64]	6. Yang (2023) [64]	6. Yang (2023) [64]
Cerebral palsy	-	-	-	-	-	-	1. 10MWT	1. 6MWT	1. Step length	-	1. FAQ-WL, WeeFIM	-	1. GMFM-total
							2. n = 123	2. n =149	2. n = 81		2. n = 42		2. n = 154
							3. −0.1 [−0.47 0.29]	3. 0.35 [−0.51 1.2]	3. 0.1 [−0.41 0.6]		3. 0.14 [−0.46 0.75]		3. 0.18 [−0.2 0.56]
							4. *p* = 0.63	4. *p* = 0.43	4. *p* = 0.71		4. *p* = 0.64		4. *p* = 0.36
							5. 0%	5. 0%	5. 0%		5. 0%		5. 0%
							6. Cortes-Perez (2022) [69]	6. Cortes-Perez (2022) [69]	6. Cortes-Perez (2022) [69]		6. Cortes-Perez (2022) [69]		6. Cortes-Perez (2022) [69]
Parkinson’s disease	-	-	-	-	-	-	1. 10MWT	1. 6MWT	1. Cadence	1. TUG	-	-	1. UPDRS-III
							2. n = 309	2. n = 252	2. n = 176	2. n = 404			2. n = 474
							3. 0.06 [0.03 0.10]	3. 42.83 [22.05 63.62]	3. 4.52 [0.94 8.10]	3. −0.56 [−1.12 0.00]			3. −2.16 [−2.48 −1.83]
							4. *p* = 0.0009	4. *p* < 0.00001	4. *p* = 0.01	4. *p* = 0.05			4. *p* < 0.00001
							5. 5%	5. 97%	5. 12%	5. 33%			5. 27%
							6. Jiang (2024) [72]	6. Xue (2023) [73]	6. Jiang (2024) [72]	6. Jiang (2024) [72]			6. Jiang (2024) [72]
Neurological disease	-	-	1. MAS	-	-	-	-	-	-	-	-	-	-
			2. n = 140										
			3. −0.29 [−0.49 −0.08]										
			4. *p* = 0.45										
			5. 0%										
			6. Garlet (2024) [77]										

1. Outcome measure, 2. number of participants, 3. effect size [95% confidence interval], 4. *p* value, 5. heterogeneity; I^2^, 6. systematic review. The color coding in the table pertains to the following: Deep green = Suggestive; Light green = Weak; Red = Nonsignificant. ^a^ The results of the meta-analysis were reported on the chronic phase of spinal cord injury. Abbreviations: 10MWT, 10-Meter Walk Test; 20MWT, 20-Meter Walk Test; 2MWT, 2-Minute Walk Test; 5MWT, 5-Meter Walk Test; 6MWT, 6-Minute Walk Test; ADL, Activities of Daily Living; BBS, Berg Balance Scale; CMSR, Chedoke–McMaster Stages of Recovery; EDSS, Expanded Disability Status Scale; FAC, Functional Ambulation Scale; FAI, Frenchay Activities Index; FAQ-WL, Functional Assessment Questionnaire Walking Scale; FIM, Functional Independence Measure; FMA-B, Fugl–Meyer Assessment-Balance; FMA-LE, Fugl–Meyer Assessment-Lower Extremity; GMFM, Gross Motor Function Measure; IADL, Instrumental Activities of Daily living; LEMS, Lower Extremity Motor Score; MAS, Modified Ashworth Scale; MBI, Modified Barthel Index; MI, Motricity Index; MSQOL-54, Multiple Sclerosis Quality of Life-54; PASS, Postural Assessment Scale for Stroke; RAND-36, RAND 36-Item Health Survey; RMI, Rivermead Mobility Index; SAS, Stroke Activity Scale; SF-36, Short-Form 36; SIS, Stroke Impact Scale; T25FW, Timed 25-Foot Walk; TUG, Timed Up and Go; UPDRS, Unified Parkinson’s Disease Rating Scale; VAS, Visual Analog Scale; WISCI, Walking Index for Spinal Cord Injury.

## Data Availability

The datasets used and/or analyzed during the current study are available from the corresponding author on reasonable request. Requests to access these datasets should be directed to Y.O., otaka119@mac.com.

## Supplementary Materials

The following supporting information can be downloaded at: https://www.mdpi.com/article/10.3390/jcm13216616/s1, Table S1: Characteristics of the included studies; Table S2: Excluded studies and the reasons for exclusion (n = 79); Table S3: RCTs included in the SRs of upper-limb devices; Table S4: RCTs included in the SRs of lower-limb devices; Table S5: AMSTAR 2 quality assessment of the included reviews; Table S6: The list of upper-limb devices included in the RCTs; Table S7: The list of lower-limb devices included in the RCTs; Table S8: Outcome measures used in the included studies; Table S9: Summary of evidence grading and statistics used in meta-analyses of upper-limb devices; Table S10: Summary of evidence grading and statistics used in meta-analyses of lower-limb devices; Table S11: PRIOR Checklist.
