# Supplementary material for: Effectiveness of Robotic Devices for Medical Rehabilitation: An Umbrella Review
Source: J Clin Med. 2024 Nov 4;13(21):6616. doi: 10.3390/jcm13216616 (PMC11546060; doi:10.3390/jcm13216616)
Supplement: Supplementary file 1 [file jcm-13-06616-s001.zip › Table S1.pdf]

**Table S1** Characteristics of the included studies

| Disease | Devices | Study | RCT[a] | Number of participants[a] | Population | Intervention group | Comparator group | Findings |
|---|---|---|---|---|---|---|---|---|
| Stroke | Upper-limb device | Carrillo (2023) [16] | 14 | 1141 | Patients with stroke aged > 18 years | RT | CT (motor relearning, functional electrical stimulation, therapeutic exercise, proprioceptive neuromuscular facilitation, and constraint-induced movement therapy) | RT was shown to accelerate the recovery of the arm in the first two weeks after the intervention. |
| | | Doumen (2023) [17] | 8 | 304 | Patients with stroke acute or early subacute and severe upper limb impairments or disability | RT | Dose different from RT, CT, non-intervention | RT showed significant improvements in upper limb impairments compared with dose-matched control programs. |
| | | Gnasso (2023) [18] | 3 | 174 | Patients with stroke and shoulder pain | RT | CT (kinesiotherapy involving passive, assistive, and active exercises, as well as stretching exercises, and conventional physical therapy techniques such as hot pack application, electrical stimulation, Kinesio taping, and non-robotic approaches) | RT exhibited significant improvements in reducing shoulder pain, enhancing joint mobility, and promoting overall motor recovery compared with conventional rehabilitation methods. |
| | | Lee (2023) [19] | 18 | 1559 | Patients with stroke | RT | CT | RT significantly improved upper limb function and hand function in patients with stroke. Upper limb function significantly improved when the RT duration was 30–60 min per session. |
| | | Yang (2023) [20] | 14 | 1275 | Patients with stroke aged > 18 years but < 80 years | RT | CT, no treatment | RT significantly improved the upper limb motor function and ADL in patients with stroke undergoing upper limb rehabilitation. |
| | | Moggio (2022) [21] | 5 | 149 | Adult patients with stroke within 1 year after onset | Hand finger robot-assisted therapy | Traditional rehabilitation | The Motricity Index significantly improved in the robotic intervention group, as compared to the control group. The QuickDASH score was significantly reduced in the exoskeleton group. The FMA-UE scores showed a significant improvement in the end-effector group. |
| | | Zhang (2022) [22] | 46 | 2533 | Adult patients with stroke for more than 3 months | RT | CT | RT had significant immediate beneficial effects on motor control and activity function of the hemiparetic upper limb in patients with stroke. However, there was no evidence of long-term effects. |
| | | Zhao (2022) [23] | 22 | 758 | Patients with stroke aged > 18 years | Robot-assisted distal training (wrist and hand) | Typical treatments such as therapist-assisted training and passive ROM exercises | RT had a significant effect on upper-extremity motor function compared to CT. |
| | | Rozevink (2021) [24] | 12 | 311 | Adult patients with stroke | Task-specific training using an assistive device | Task-specific training, in which usual care was provided without using an assistive device | Both the intervention and comparator groups exhibited improvements in upper-limb performance after stroke. During the sub-acute phase after stroke, task-specific training with assistive arm devices was more effective than task-specific usual care in reducing upper-limb impairment. During the chronic phase, both groups showed similar improvements. No differences between the two types of training were found at follow-up measurements. |
| | | Wu (2021) [25] | 41 | 1916 | Patients with stroke aged > 18 years | RT | Dose-matched conventional physical/occupational therapy | RT significantly improved the FMA-UE scores of patients with stroke, with a small effect size. |
| | | Chen (2020) [26] | 35 | 2241 | Patients with stroke and upper-limb motor dysfunction | RT | Therapist-mediated training | RT was slightly superior with respect to motor impairment recovery and was noninferior to therapist-mediated training in improving arm capacity, ADL, and social participation, which supported the use of RT in clinical practice. |
| | | Chien (2020) [27] | 11 | 493 | Patients with subacute stroke | RT | CT and physical therapy | RT produced similar benefits but was not significantly superior to usual care in improving functioning and disability in patients diagnosed with stroke within 6 months. |
| | | Ferreira (2018) [28] | 37[b] | 1154 | Patients with stroke and limited upper-limb function | RT | Other interventions, minimal intervention, placebo, or sham | RT had a small effect on motor control and a medium effect on strength in patients with limited upper-limb function. |
| | | Mehrholz (2018) [29] | 45 | 1619 | Patients with stroke aged >18 years | Electromechanical and robot-assisted arm training | Other interventions, placebo interventions, or no treatment | RT improved the ADL in patients after stroke, as well as the function and muscle strength of the affected arm. Adverse events, such as injuries and pain, were seldom described; thus, these devices could be applied for rehabilitation. |
| | | Bertani (2017) [30] | 14 | 576 | Patients with stroke | RT | Any other type of nonrobotic intervention (CT, usual care, etc.) | RT was more effective than CT in improving upper-limb motor function recovery, especially in patients with chronic stroke. No significant improvements in the reduction of muscle tone or ADL were observed. |
| | | Kim (2017) [31] | 6 | 278 | Adult patients aged > 20 years with early-stage (acute or subacute) stroke within 3 months after onset | RT | Traditional rehabilitation, self-guided therapy, individual arm therapy | RT with three-dimensional movement and high degree of freedom had a positive effect on upper-extremity motor function recovery in patients with early-stage stroke. |
| | | Veerbeek (2017) [32] | 44 | 1362 | Patients with stroke | RT | Nonrobotic treatment | Meta-analyses showed significant but small improvements in the motor control and muscle strength of the paretic arm, a negative effect on muscle tone, and no effects on upper-limb capacity and basic ADL. Shoulder/elbow robotics produced a small but significant effect on motor control and muscle strength, whereas elbow/wrist |

| | | | | | | | |
|---|---|---|---|---|---|---|---|
| | | | | | | | robotics had a small but significant effect on motor control. Effects on motor control were small and specific to the joints targeted by RT, whereas no generalization was found for the improvements in upper-limb capacity. |
| | Zhang (2017) [33] | 16 | 496 | Patients with stroke aged > 18 years | RT | CT (standard/usual care or intensive training without robotic support) | Upper-limb function significantly improved when RT was used with CT, as compared with CT alone. A significant difference was found in motor recovery between the CT-only and RT groups (RT only or RT combined with CT) during the chronic stages of stroke, but not during the acute or subacute stages. |
| | Norouzi (2012) [34] | 11 | 413 | Adult patients with stroke | RT | CT (either standard/usual care or intensive training) | After matching the duration/intensity of CT with that of RT, no differences in motor recovery, ADL, strength, and motor control were detected between the intensive CT and RT groups. However, depending on the stage of recovery, extra sessions of RT in addition to regular CT were more beneficial than regular CT alone for the motor recovery of the hemiparetic shoulders and elbows in patients with stroke; gains were similar to those observed in intensive CT. |
| Lower-limb device | Leow (2023) [35] | 20 | 785 | Adult patients with stroke aged > 18 years acute, subacute, or chronic | (1) RT<br>(2) RT with CT | Placebo, no intervention or any CT | RT or RT with CT improved walking ability and increased walking speed, with a small effect size among patients with stroke post-intervention. A significant improvement was also observed in walking ability among patients with stroke at follow-up. |
| | Yang (2023) [36] | 10 | 323 | Adult patients with stroke aged > 18 years for more than 6 months | RT | Overground gait training, treadmill gait training and conventional rehabilitation programs (Bobath approach, postural control training, manual-assisted walking training, strengthening exercises, etc.) | RT improved gait function, balance and endurance in patients with chronic stroke, with no serious adverse events. To achieve a better effect, the total training time should be more than 5 hours when RT is used as a therapeutic intervention. |
| | Zhu (2023) [37] | 17 | 449 | Adult patients aged > 18 years with stroke | RT plus physical therapy | Conventional rehabilitation treatment or therapist-assisted gait training | RT with CT significantly improved balance function compared with the control group. In the subgroup of patients with an onset of greater than 6mos, RT was more effective in improving the cadence score because of the lack of heterogeneity and the result was steady. There were no significant differences in lower limb motor function, walking speed, and endurance between the treatment and control groups. |
| | Calafiore (2022) [38] | 14 | 576 | Patients with subacute stroke within 6 months after onset | RT | Conventional rehabilitation (strengthening exercises, Bobath approach, postural control training, parallel walking training, and manual walking training-assisted over-ground therapy) | The use of RT associated with conventional rehabilitation for gait recovery was effective in patients with subacute stroke, albeit not superior to only conventional rehabilitation. |
| | Baronchelli (2021) [39] | 13 | 445 | Adult patients with stroke | RT using Lokomat | Conventional physical therapy, treadmill training | Most studies indicated the beneficial effects of RT using Lokomat on balance recovery in stroke survivors, which were at least comparable to those of conventional physical therapy. However, owing to the limited number of studies and their high heterogeneity, further research should be performed to draw more solid and definitive conclusions. |
| | Nedergard (2021) [40] | 13 | 412 | Adult patients aged > 18 years with acute, subacute, or chronic stroke | RT | Nonrobotic gait rehabilitation | The few relevant studies and review syntheses revealed a very low certainty in current evidence for employing RT to improve gait biomechanics after stroke. |
| | Wang (2021) [41] | 19 | 517 | Patients with cerebral vascular accident | RT aimed at recovering balance function | CT (e.g., regular physical therapy, manually assisted BWST) | RT was effective in improving the balance function of stroke survivors. |
| | Hsu (2020) [42] | 14 | 821 | Patients with stroke within 6 months after onset | Technology-assisted gait training with body weight support | Conventional over-ground training | No significant differences across all outcome categories were found between technology-assisted gait training with body weight support and conventional over-ground training in the robotics subgroup, body weight-supported treadmill subgroup, or both subgroups combined in both the short and long terms. Further subgroup analyses also revealed nonsignificant differences across all outcomes in the initially ambulatory subgroup, non-ambulatory subgroup, or subgroup with stroke duration of < 3 months. |
| | Maranesi (2020) [43] | 9 | 517 | Adult patients with stroke aged > 60 years | RT | CT | RT was more effective in patients with subacute stroke with lower Function Ambulation Assessment scores, showing significant changes in independent walking ability. |
| | Mehrholz (2020) [44] | 62 | 2440 | Patients with stroke aged > 18 years | RT plus physical therapy | Physical therapy | The combination of RT and physical therapy increased the odds of participants in becoming independent in walking and increased the mean walking velocity; however, it did not improve the mean walking capacity. |
| | Moucheboeuf (2020) [45] | 54 | 1466 | Patients with stroke | Comparison of the following four types:<br>(1) RT with CT and BWST<br>(2) RT with BWST<br>(3) RT with BWST<br>(4) RT with CT, BWST, and FES | (1) CT<br>(2) CT<br>(3) BWST<br>(4) RT with CT and BWST | RT combined with physical therapy and BWST appeared to be an efficient intervention for gait recovery after stroke. |
| | Postol (2019) [46] | 3 | 322 | Adult patients aged > 18 years with acquired brain injury | RT with/ without body weight support | CT, alternative robotics, sham, or no intervention | No differences in the 6-Minute Walk Test, Timed Up and Go Test, or 10-Meter Walk Test were observed between the RT and control groups. The Berg Balance Scale outcomes were significantly better in controls. No severe adverse events occurred. |
| | Zheng (2019) [47] | 31 | 1249 | Patients with stroke | RT with/without other treatments | Treatment and care with routine methods, including physical therapy | RT might have a significant effect on improving the balance function among patients with stroke, as compared with those not using these devices, as indicated by increases in the Berg Balance Scale score and Fugl-Meyer |

| | | | | | | | | |
|---|---|---|---|---|---|---|---|---|
| | | | | | | | and other common rehabilitation approaches | Assessment-Balance score, as well as across subgroups of different types of robotic devices, RT combined with another intervention or not, and differences in duration and intensity of intervention. |
| | | Bruni (2018) [48] | 13 | 673 | Patients with stroke | Comparison of the following three types:<br>(1) RT<br>(2) RT with and without FES<br>(3) RT (exoskeleton robots) | (1) Conventional physical therapy<br>(2) Conventional physical therapy<br>(3) RT (end-effector robots) | The findings of this systematic review further support the use of RT for improving the motor function in patients with stroke, but when this is coupled with conventional physical therapy. The earlier the training starts, the better the gait recovery. |
| | | Cho (2018) [49] | 7 | 220 | Adult patients aged > 18 years with acute or subacute stroke within 3 months after onset | RT with physical therapy or usual care | CT | RT was effective in improving the walking ability of patients with subacute stroke. Significant improvements in gait speed, Functional Ambulatory Category score, and Rivermead Mobility Index score were observed with RT, as compared with conventional rehabilitation therapy. Aggressive weight support and gait training with a robotic device at an early stage were helpful, and RT should be applied according to patients' functional level and stroke onset time. |
| | | Mehrholz (2017a) [50] | 36 | 1472 | Patients with stroke aged > 18 years | RT with physical therapy or usual care | Physical therapy or usual care | The use of RT in combination with physical therapy increased the chance of regaining independent walking ability in patients after stroke. |
| | | Hesse (2013) [51] | 9 | 564 | Patients with central nervous system lesion | RT plus physical therapy with/ without FES or tDCS | Physical therapy | RT was more effective in improving walking independence in patients with stroke. |
| | | Mehrholz (2012) [52] | 18 | 885 | Patients with stroke aged > 18 years | Comparison of the following two types:<br>(1) RT (exoskeleton)<br>(2) RT (end-effector) | (1) Physical therapy or usual care<br>(2) Physical therapy or usual care | The end-effector subgroup exhibited improvements in walking independence; in contrast, the exoskeleton subgroup did not show any significant effect. The rate of independent walking was significantly higher in the end-effector subgroup than in the exoskeleton subgroup. |
| | | Ada (2010) [53] | 4 | 549 | Adult non-ambulatory patients with subacute stroke | Any type of mechanically assisted walking (RT, treadmill, etc.) with body weight support | Over-ground walking (including any type of assistance from therapists or aids, such as orthoses and sticks) | Mechanically assisted walking with body weight support was more effective than over-ground walking for increasing independent walking in non-ambulatory patients early after stroke. Furthermore, it was not detrimental to walking speed or capacity. |
| | Upper-/Lower-limb device | Saragih (2023) [54] | 52 | 2774 | Patients with stroke | RT | Usual care | The quality of movement and balance in patients with stroke improved more with RT than with CT. No significant differences were found for other outcomes, except that pain reduction was better with RT than the CT. |
| | | Lo (2017) [55] | 51 | 1798 | Patients with stroke aged > 18 years | RT | CT | RT was just as effective as conventional training for upper-limb motor movement, lower-limb walking mobility, and ADL. RT led to better outcomes than conventional training in patients with severe lower-limb impairment. |
| Spinal cord injury | Lower-limb device | Huang (2024) [56] | 3 | 202 | Patients with SCI | BWST including RT | CT | BWST improved lower extremity motor score in patients with SCI. No significant difference was observed among the three BWST methods, but RT may produce the best effect. |
| | | Wan (2024) [57] | 11 | 408 | Patients with SCI | RT | CT | RT had a positive improvement on lower extremity strength and cardiopulmonary function in patients with SCI. however, the effect on static pulmonary function is relatively limited. |
| | | Li (2023) [58] | 19 | 770 | Patients aged > 15 years with incomplete SCI | RT | CT | RT had a positive effect on cardiopulmonary fitness and exercise capacity in patients with incomplete SCI. |
| | | Fang (2020) [59] | 7 | 222 | Patients with SCI | RT | Other training modalities or no training | RT could improve spasticity and walking ability in patients with SCI. The probable reason for the absence of significant change in pain after RT was the floor effect. RT was beneficial for normalizing the muscle tone and improving the lower-extremity function in patients with SCI without causing additional pain. |
| | | Nam (2017) [60] | 10 | 502 | Patients aged > 16 years with incomplete, traumatic or nontraumatic, nonprogressive SCI, as defined by ASIA Impairment Scale grade B, C, or D | Comparison of the following four types:<br>(1) RT<br>(2) RT<br>(3) RT<br>(4) RT | (1) Conventional over-ground training<br>(2) BWST<br>(3) Non-gait-specific training<br>(4) No treatment | Compared to conventional over-ground training, RT improved the mobility-related outcomes to a greater degree in patients with incomplete SCI, particularly during the acute stage. RT is a promising treatment for restoring functional walking and improving the locomotor ability, which might enable patients with SCI to maintain a healthy lifestyle and increase their physical activity levels. |
| | | Mehrholz (2017b) [61] | 5 | 586 | Patients with traumatic SCI | RT or BWST | Over-ground gait training and/or other forms of physical therapy | RT and BWST did not increase the walking speed more than over-ground gait training and other forms of physical therapy, and their effects on walking distance were unclear. |
| | | Fisahn (2016) [62] | 11 | 633 | Patients with SCI and gait disorder aged > 18 years but < 75 years | Comparison of the following three types:<br>(1) RT (exoskeleton)<br>(2) RT (exoskeleton)<br>(3) RT (exoskeleton) | (1) Knee-ankle-foot orthoses<br>(2) Other rehabilitation strategies<br>(3) Knee-ankle-foot orthoses for assistance or other rehabilitation strategies | No consistent benefit from rehabilitation using RT versus conventional methods was observed in patients with chronic SCI. |
| | Upper-/lower-limb device | Cheung (2017) [63] | 11 | 443 | Patients with complete or incomplete SCI | RT for upper- or lower-limb | Other treatment approaches or no treatment | RT could serve as an adjunct therapy for physical and functional recovery in patients with SCI. RT was more effective than other treatment approaches or no treatment in improving walking independence; however, RT did not lead to a significant improvement in walking speed. |

| | | | | | | | | |
|---|---|---|---|---|---|---|---|---|
| Multiple sclerosis | Lower-limb device | Yang (2023) [64] | 16 | 536 | Patients with MS | RT | Conventional overground gait training or other gait training protocol | RT exhibited significant improvement with low heterogeneity with respect to primary outcomes (i.e., walking velocity and walking endurance) and some secondary outcomes (i.e., mobility, balance, and fatigue). |
| | | Bowman (2021) [65] | 12 | 248 | Adult patients with MS | RT for balance/gait rehabilitation | CT, over-ground walking training, usual care, no treatment | RT improved the balance and gait outcomes in a clinically meaningful manner. Considering its several advantages in terms of safety, motor assistance, and intensity of training provided, RT should be promoted for patients with MS and severe disability in a multimodal rehabilitation context as an opportunity to maximize recovery. |
| | | Yeh (2020) [66] | 10 | 343 | Patients with MS | RT | Conventional walking therapy | The meta-analysis indicated comparable effectiveness between RT and conventional walking therapy with respect to walking performance, quality of life, pain, and ADL. RT was even statistically superior to conventional walking therapy in improving perceived fatigue, spasticity, and global mobility after the intervention. |
| | | Sattelmayer (2019) [67] | 9 | 369 | Adult patients with MS | RT | Non-robot-assisted gait graining | RT was not significantly more effective than conventional over-ground walking in training walking in patients in MS. |
| Cerebral palsy | Lower-limb device | Conner (2022) [68] | 8 | 188 | Patients with CP | RT | Traditional gait training, functional exercises | RT did not provide greater benefits than the standard of care in improving mobility in individuals with CP. |
| | | Cortes-Perez (2022) [69] | 15 | 413 | Children with CP | RT | CT or treadmill therapy | RT was more effective than CT in improving gait speed, walking distance, and walking-running and jumping abilities, just at the end of the therapy (post-intervention). RT was only superior to treadmill therapy in improving step length at the 2-month follow-up. |
| | | Llamas-Ramos (2022) [70] | 7 | 206 | Patients with CP aged 4–14 years | RT with CT | CT | No consensus exists regarding the effectiveness of RT. However, it seems clear that it has presented a good complement to conventional physical therapy, although not a therapy as itself. More studies are required to prove and evaluate the extent to which RT can be applied in the treatment of children with CP. |
| | | Lefmann (2017) [71] | 3 | 486 | Pediatric patients aged 5–17 years with gait disorders of any etiology (neurological, orthopedic, or developmental) | RT | Alternate form of feedback, individual physical therapy only, alternative exercises or combined with FES | Evidence regarding the use of RT for children with gait disorders is weak and inconsistent. If clinicians (and their clients) choose to use RT, they should closely monitor individual progress with appropriate outcome measures, as well as the adverse events. |
| Parkinson's disease | Lower-limb device | Jiang (2024) [72] | 17 | 670 | Patients with PD and motor dysfunction | RT with/without CT | CT | RT improved balance, walking, and gait performance and significantly improved lower extremity function in patients with PD. |
| | | Xue (2023) [73] | 14 | 572 | Patients with PD aged > 18 years | RT | Regular lower extremity rehabilitation or treadmill therapy | RT was improvement of motor function and balance function in patients with PD after 4–12 weeks of intervention. |
| | | Alwardat (2018) [74] | 7 | 286 | Patients with PD | RT | Conventional exercise and/or treadmill gait training | RT significantly improved the UPDRS-III score after the intervention and after 1-month follow-up. RT also improved the stride length and walking speed. Balance, as measured by the Berg Balance Scale, showed significant improvements after the intervention and at 1-month follow-up. |
| Neurological disease | Upper-limb device | Ferreira (2021) [75] | 12 | 845 | Patients with limited upper-limb function caused by any health condition | RT | Minimal intervention (no interventions, sham, placebo, and waiting list) or other interventions | For patients with upper-limb neurological dysfunction, low-quality evidence supports RT over minimal intervention in terms of improving individual participation in the short term. The existing low- to very low-quality evidence does not support RT over other interventions in either the short- or medium-term follow-up periods with respect to community participation. |
| | | Dixit (2019) [76] | 21 | 741 | Patients with loss of upper-limb function | RT with standard care | Standard physical therapy | Studies on stroke showed a clear definiteness in the improvement of upper-extremity function. On the contrary, there are not enough quality trials on CP and MS to establish the efficacy of RT. |
| | Lower-limb device | Garlet (2024) [77] | 9 | 347 | Patients with SCI , stroke, persistent vegetative state, or brain injury | RT | CT or placebo | RT was safe for patients with neurological injury and reduced spasticity in patients with stroke. The effects on muscle strength, functionality, gait/balance, and level of consciousness remained uncertain. |

[a] The number of RCT/participants presented here is derived from studies focused on robotic devices.
[b] One study (Volpe, 1999), which was a follow-up study of the study conducted by Aisen (1997), was excluded.
Abbreviations: ADL, activities of daily living; ASIA, American Spinal Injury Association; BWST, body-weight support training; CP, cerebral palsy; CT, conventional therapy; FES, functional electrical stimulation; FMA-UE, Fugl-Meyer Assessment for Upper Extremity; MS, multiple sclerosis; PD, Parkinson's disease; QuickDASH, Quick version of the Disabilities of the Arm, Shoulder, and Hand questionnaire; RCT, randomized controlled trial; ROM, range of motion; RT, robot-assisted training; SCI, spinal cord injury; tDCS, transcranial Direct Current Stimulation; UPDRS-III, Unified Parkinson's Disease Rating Scale-III
