# Supplementary material for: Effectiveness of Robotic Devices for Medical Rehabilitation: An Umbrella Review
Source: J Clin Med. 2024 Nov 4;13(21):6616. doi: 10.3390/jcm13216616 (PMC11546060; doi:10.3390/jcm13216616)
Supplement: Supplementary file 1 [file jcm-13-06616-s001.zip › Table S10.pdf]

Table S10 Summary of evidence grading and statistics used in meta-analyses of lower-limb devices

Disease	Study	Outcome categories	Outcomes	Subgroups	Details of subgroups	Number of participants	Number of RCTs	Effect model	Effect size	Effect size (95% CI)	*P*	I^2	Grading	Classification of dose matching										
														A	B	C	D	E	F	G	H	I	J	Unknown
Stroke	Leow (2023) [35]	Walking independence	FAC	-	-	371	11	Random	SMD	0.21 [0.01 0.42]	0.04	0	Weak	6	3	-	-	-	-	-	-	-	2	-
		Walking speed	5MWT, 10MWT, gait speed (measured using GAITRite or WalkWay or motioncapture system)	-	-	469	15	Random	SMD	0.23 [0.01 0.46]	0.04	33	Weak	5	7	-	-	-	-	-	-	-	2	1
		Walking capacity	6MWT	-	-	506	13	Random	SMD	0.04 [-0.14 0.21]	0.66	0	Nonsignificant	6	6	-	-	-	-	-	-	-	1	-
	Zhu (2023) [37]	Walking independence	FAC	-	-	170	5	Fixed	MD	0.00 [-0.09 0.10]	0.94	36	Nonsignificant	4	-	-	-	-	1	-	-	-	-	-
		Balance capacity	BBS	-	-	281	9	Random	MD	1.39 [-0.87 3.65]	0.23	84	Nonsignificant	4	1	-	2	-	1	-	-	-	1	-
		Balance capacity	BBS	Detail of intervention	robot only	157	4	Random	MD	-0.61 [-1.07 -0.14]	0.010	0	Weak	1	-	-	2	-	-	-	-	-	1	-
		Balance capacity	BBS	Detail of intervention	robot + conventional therapy	124	5	Random	MD	3.57 [0.89 6.26]	0.009	59	Weak	3	1	-	-	-	1	-	-	-	-	-
		Walking capacity	6MWT	-	-	222	7	Random	SMD	-0.69 [-1.57 0.20]	0.13	89	Nonsignificant	3	1	-	2	-	-	-	-	-	1	-
		Walking capacity	6MWT	Sensitivity analysis	-	159	6	Random	SMD	-0.29 [-0.63 0.04]	0.09	8	Nonsignificant	3	1	-	2	-	-	-	-	-	1	-
		Walking speed	10MWT	-	-	126	5	Random	MD	0.02 [-0.03 0.08]	0.41	78	Nonsignificant	3	2	-	-	-	-	-	-	-	-	-
		Gait index	Cadence	-	-	130	4	Random	SMD	-2.02 [-9.04 5.00]	0.57	94	Nonsignificant	3	1	-	-	-	-	-	-	-	-	-
		Gait index	Cadence	Period of onset	< 6 months	93	2	Random	MD	-6.47 [-10.18 -2.76]	0.0006	63	Weak	2	-	-	-	-	-	-	-	-	-	-
		Gait index	Cadence	Period of onset	> 6 months	37	2	Random	SMD	2.97 [1.35 4.59]	0.0003	0	Weak	1	1	-	-	-	-	-	-	-	-	-
	Calafiore (2022) [38]	Walking independence	FAC	-	-	246	6	Random	MD	-0.09 [-0.22 0.03]	0.15	29	Nonsignificant	5	1	-	-	-	-	-	-	-	-	-
	Baronchelli (2021) [39]	Balance capacity	BBS	-	-	284	9	Fixed	MD	0.17 [-0.26 0.60]	0.44	89	Nonsignificant	4	1	-	2	-	-	-	-	-	2	-
		Balance capacity	TUG	-	-	125	5	Fixed	MD	-3.40 [-4.35 -2.44]	< 0.00001	92	Weak	2	1	-	-	-	-	-	-	-	2	-
		Walking capacity	RMI	-	-	184	4	Fixed	MD	0.40 [0.26 0.55]	< 0.00001	0	Weak	2	1	-	-	-	-	-	-	-	1	-
	Nedergard (2021) [40]	Walking speed	Walking speed	-	Overall	375	12	Random	MD	0.00 [-0.05 0.05]	0.97	93	Nonsignificant	3	6	-	2	1	-	-	-	-	-	-
		Walking speed	Walking speed	Velocity during assessment	Self-selected velocity	190	7	Random	MD	-0.04 [-0.07 -0.00]	0.03	53	Weak	1	4	-	1	1	-	-	-	-	-	-
		Walking speed	Walking speed	Velocity during assessment	Fasted velocity	185	5	Random	MD	0.03 [-0.03 0.10]	0.33	86	Nonsignificant	2	2	-	1	-	-	-	-	-	-	-
		Gait index	Cadence	-	Overall	349	9	Random	MD	1.44 [-2.34 5.22]	0.46	92	Nonsignificant	4	4	-	-	-	-	-	-	-	1	-
		Gait index	Cadence	Velocity during assessment	Self-selected velocity	198	5	Random	MD	-1.93 [-6.71 2.86]	0.43	87	Nonsignificant	2	3	-	-	-	-	-	-	-	-	-
		Gait index	Cadence	Velocity during assessment	Fasted velocity	151	4	Random	MD	5.77 [-0.08 11.62]	0.05	92	Nonsignificant	2	1	-	-	-	-	-	-	-	1	-
		Gait index	Step length	-	Overall	332	9	Random	MD	1.22 [-0.10 2.54]	0.07	89	Nonsignificant	4	5	-	-	-	-	-	-	-	-	-
		Gait index	Step length	Affected side or not	Affected side	175	5	Random	MD	2.67 [1.55 3.80]	< 0.00001	65	Weak	2	3	-	-	-	-	-	-	-	-	-
		Gait index	Step length	Affected side or not	Non-affected side	157	4	Random	MD	0.21 [-0.66 1.07]	0.64	41	Nonsignificant	2	2	-	-	-	-	-	-	-	-	-
		Gait index	Stride length	-	-	161	5	Random	MD	2.86 [0.46 5.25]	0.02	66	Weak	1	3	-	-	1	-	-	-	-	-	-
		Gait index	Temporal symmetry	-	-	196	5	Random	MD	0.09 [0.04 0.15]	0.001	90	Weak	2	3	-	-	-	-	-	-	-	-	-
		Gait index	Spatial symmetry	-	-	279	7	Random	MD	-0.01 [-0.07 0.04]	0.67	79	Nonsignificant	1	4	-	2	-	-	-	-	-	-	-
	Wang (2021) [41]	Balance capacity	BBS	-	-	517	13	Random	MD	3.58 [1.89 5.28]	< 0.0001	41	Weak	6	1	-	2	-	-	1	-	-	1	2
		Balance capacity	BBS	Period of onset	< 6 months	354	7	Random	MD	5.40 [3.94 6.86]	< 0.00001	0	Weak	3	-	-	-	-	-	1	-	-	1	2
		Balance capacity	BBS	Period of onset	> 6 months	129	5	Random	MD	1.61 [-0.02 3.25]	0.05	0	Nonsignificant	2	1	-	2	-	-	-	-	-	-	-
		Balance capacity	BBS	Type of device	End-effector	128	3	Random	MD	3.08 [-1.47 7.63]	0.18	34	Nonsignificant	2	-	-	-	-	-	-	-	-	1	-
		Balance capacity	BBS	Type of device	Exoskeleton	389	10	Random	MD	3.73 [1.83 5.63]	0.0001	45	Weak	4	1	-	2	-	-	1	-	-	-	2
		Balance capacity	BBS	Training intensity	< 10 hours	228	6	Random	MD	2.31 [-0.62 5.24]	0.12	49	Nonsignificant	3	1	-	1	-	-	1	-	-	-	-
		Balance capacity	BBS	Training intensity	> 10 hours	289	7	Random	MD	4.53 [2.31 6.75]	< 0.0001	37	Weak	3	-	-	1	-	-	1	-	-	-	2
	Hsu (2020) [42]	Motor impairment	CMSR, FMA-LE, MI	-	-	389	7	Random	SMD	0.15 [-0.09 0.40]	0.22	27	Nonsignificant	-	6	-	-	-	-	-	-	-	1	1
		Motor impairment	CMSR, FMA-LE, MI	Follow-up duration	Follow-up	232	4	Random	SMD	0.28 [-0.00 0.57]	0.05	11	Nonsignificant	-	-	-	-	-	-	-	-	-	-	4
		Walking capacity	EU walking, EMS, FAC, Motor Assessment Scale, MeEAP, MMAS, RMA, RMI	-	-	619	10	Random	SMD	0.19 [-0.10 0.49]	0.20	67	Nonsignificant	7	1	-	-	-	-	-	-	-	2	1
		Walking capacity	EU walking, EMS, FAC, Motor Assessment Scale, MeEAP, MMAS, RMA, RMI	Follow-up duration	Follow-up	416	6	Random	SMD	0.05 [-0.60 0.69]	0.89	89	Nonsignificant	-	-	-	-	-	-	-	-	-	-	6
		Walking speed	10MWT, 2MWT, 5MWT	-	-	487	8	Random	MD	0.01 [-0.08 0.09]	0.89	66	Nonsignificant	7	-	-	-	-	-	-	-	-	1	1
		Walking speed	10MWT, 2MWT, 5MWT	Follow-up duration	Follow-up	393	6	Random	MD	0.08 [-0.05 0.22]	0.23	83	Nonsignificant	-	-	-	-	-	-	-	-	-	-	6
		Walking capacity	2MWT, 6MWT, 5MWT, peak VO2, VO2 during the 5MWT	-	-	471	7	Random	SMD	-0.04 [-0.36 0.28]	0.82	66	Nonsignificant	8	-	-	-	-	-	-	-	-	-	1
		Walking capacity	2MWT, 6MWT, 5MWT, peak VO2, VO2 during the 5MWT	Follow-up duration	Follow-up	367	5	Random	SMD	0.03 [-0.28 0.35]	0.84	53	Nonsignificant	-	-	-	-	-	-	-	-	-	-	5
		Balance capacity	BBS, Brunel Balance Assessment, PASS, TUG	-	-	223	5	Random	SMD	0.20 [-0.40 0.80]	0.51	79	Nonsignificant	4	-	-	-	-	-	-	-	-	1	-
		Balance capacity	BBS, Brunel Balance Assessment, PASS, TUG	Follow-up duration	Follow-up	375	3	Random	SMD	-0.03 [-0.23 0.17]	0.78	0	Nonsignificant	-	-	-	-	-	-	-	-	-	-	3
		ADL	ADL-IADL, FAI, FIM, SAS, SIS	-	-	586	9	Random	SMD	0.14 [-0.13 0.42]	0.30	60	Nonsignificant	8	-	-	-	-	-	-	-	-	1	1
		ADL	ADL-IADL, FAI, FIM, SAS, SIS	Follow-up duration	Follow-up	415	6	Random	SMD	0.19 [-0.50 0.88]	0.59	90	Nonsignificant	-	-	-	-	-	-	-	-	-	-	6
	Mehrholz (2020) [44]	Walking independence	FAC, FIM, RMI	-	-	1567	38	Random	OR	2.14 [1.57 2.92]	< 0.00001	6	Suggestive	13	13	-	2	-	-	1	-	1	4	4
		Walking independence	FAC, FIM, RMI	Follow-up duration	Follow-up	496	6	Random	OR	1.93 [0.72 5.13]	0.19	79	Nonsignificant	3	-	-	1	-	-	-	-	-	2	-
		Walking speed	Walking speed	-	-	1600	42	Random	MD	0.06 [0.02 0.10]	0.004	60	Weak	23	12	-	2	-	-	-	-	-	3	2
		Walking speed	Walking speed	Follow-up duration	Follow-up	727	13	Random	MD	0.07 [-0.03 0.17]	0.18	74	Nonsignificant	5	5	-	1	-	-	-	-	-	1	1
		Walking capacity	6MWT	-	-	983	24	Random	MD	10.86 [-5.72 27.44]	0.20	42	Nonsignificant	11	8	-	1	-	-	-	-	1	1	2
		Walking capacity	6MWT	Follow-up duration	Follow-up	612	11	Random	MD	7.76 [-21.47 36.99]	0.60	60	Nonsignificant	5	4	-	1	-	-	-	-	-	-	1
		Other measures	Lost to study, dropout	-	Overall	2440	62	Random	RD	-0.02 [-0.04 0.00]	0.08	3	Nonsignificant	33	17	-	2	-	-	1	-	1	4	4
		Other measures	Lost to study, dropout	Type of device	End-effector	716	14	Random	RD	-0.02 [-0.05 0.02]	0.37	0	Nonsignificant	9	1	-	-	-	-	-	-	1	3	-
		Other measures	Lost to study, dropout	Type of device	Exoskeleton	1496	41	Random	RD	-0.03 [-0.05 0.00]	0.09	24	Nonsignificant	23	10	-	2	-	-	1	-	-	1	4
		Other measures	Lost to study, dropout	Type of device	Mobile device	146	4	Random	RD	0.00 [-0.05 0.06]	0.93	0	Nonsignificant	1	3	-	-	-	-	-	-	-	-	-
		Other measures	Lost to study, dropout	Type of device	Ankle device	82	3	Random	RD	0.01 [-0.09 0.11]	0.89	0	Nonsignificant	-	3	-	-	-	-	-	-	-	-	-
		Other measures	Death	-	-	2440	62	Random	RD	0.00 [-0.01 0.01]	0.82	0	Nonsignificant	33	17	-	2	-	-	1	-	1	4	4
		Walking independence	FAC, FIM, RMI	Trial methods	Adequate sequence generation process	1049	22	Random	OR	1.93 [1.26 2.96]	0.003	25	Weak	9	5	-	2	-	-	-	-	1	3	2
		Walking independence	FAC, FIM, RMI	Trial methods	Adequate concealed allocation	905	18	Random	OR	1.90 [1.23 2.95]	0.004	29	Weak	7	4	-	2	-	-	-	-	1	3	1
		Walking independence	FAC, FIM, RMI	Trial methods	Blinded assessors for primary outcome	837	17	Random	OR	1.86 [1.22 2.84]	0.004	23	Weak	8	4	-	1	-	-	-	-	1	1	2
		Walking independence	FAC, FIM, RMI	Trial methods	Without incomplete outcome data	590	14	Random	OR	2.23 [1.16 4.29]	0.02	29	Weak	7	4	-	1	-	-	-	-	1	-	1
		Walking independence	FAC, FIM, RMI	Trial methods	Excluding the largest study	1417	37	Random	OR	1.74 [1.26 2.39]	0.0007	0	Suggestive	16	11	-	2	-	-	1	-	1	3	3
		Walking independence	FAC, FIM, RMI	Duration after stroke	≤ 3 months	1243	22	Random	OR	1.96 [1.47 2.62]	< 0.00001	0	Suggestive	13	4	-	-	-	-	1	-	-	2	2
		Walking independence	FAC, FIM, RMI	Duration after stroke	> 3 months	461	15	Random	OR	1.20 [0.40 3.65]	0.74	29	Nonsignificant	4	7	-	2	-	-	-	-	-	1	1
		Walking independence	FAC, FIM, RMI	Ambulatory status at the start of the study	Included independent walkers	500	15	Random	OR	1.38 [0.45 4.20]	0.57	-	Nonsignificant	3	7	-	2	-	-	-	-	1	-	2
		Walking independence	FAC, FIM, RMI	Ambulatory status at the start of the study	Included dependent and independent walkers	340	9	Random	OR	1.90 [1.11 3.25]	0.02	0	Weak	5	1	-	-	-	-	-	-	-	2	1
		Walking independence	FAC, FIM, RMI	Ambulatory status at the start of the study	Included dependent walkers	732	14	Random	OR	2.02 [1.27 3.22]	0.003	30	Weak	9	3	-	-	-	-	1	-	-	1	-
		Walking speed	Walking speed	Ambulatory status at the start of the study	Included independent walkers	715	22	Random	MD	0.02 [-0.04 0.09]	0.49	56	Nonsignificant	11	7	-	2	-	-	-	-	-	1	1
		Walking speed	Walking speed	Ambulatory status at the start of the study	Included dependent and independent walkers	226	7	Random	MD	0.04 [-0.04 0.11]	0.34	0	Nonsignificant	4	1	-	-	-	-	-	-	-	1	1
		Walking speed	Walking speed	Ambulatory status at the start of the study	Included dependent walkers	591	11	Random	MD	0.09 [0.02 0.15]	0.008	67	Weak	7	3	-	-	-	-	-	-	-	1	-
		Walking independence	FAC, FIM, RMI	Type of electromechanical device	End-effector	598	11	Random	OR	1.90 [0.99 3.63]	0.05	50	Nonsignificant	6	1	-	-	-	-	-	-	1	3	-
		Walking independence	FAC, FIM, RMI	Type of electromechanical device	Exoskeleton	685	18	Random	OR	2.11 [1.36 3.29]	0.0010	0	Weak	9	4	-	1	-	-	1	-	-	-	3
		Walking independence	FAC, FIM, RMI	Type of electromechanical device	Mobile device	106	3	Random	OR	Not estimatable	-	-	Nonsignificant	-	3	-	-	-	-	-	-	-	-	-
		Walking independence	FAC, FIM, RMI	Type of electromechanical device	Ankle device	63	2	Random	OR	Not estimatable	-	-	Nonsignificant	-	2	-	-	-	-	-	-	-	-	-
		Walking speed	Walking speed	Type of electromechanical device	End-effector	665	13	Random	MD	0.12 [0.05 0.19]	0.001	69	Weak	10	1	-	-	-	-	-	-	-	2	-
		Walking speed	Walking speed	Type of electromechanical device	Exoskeleton	742	23	Random	MD	-0.00 [-0.05 0.04]	0.87	33	Nonsignificant	12	6	-	2	-	-	-	-	-	1	2
		Walking speed	Walking speed	Type of electromechanical device	Mobile device	146	4	Random	MD	0.12 [-0.07 0.30]	0.21	67	Nonsignificant	1	3	-	-	-	-	-	-	-	-	-
		Walking speed	Walking speed	Type of electromechanical device	Ankle device	58	2	Random	MD	0.11 [-0.10 0.31]	0.30	62	Nonsignificant	-	2	-	-	-	-	-	-	-	-	-
		Walking capacity	6MWT	Type of electromechanical device	End-effector	416	7	Random	MD	31.22 [10.35 52.08]	0.003	0	Weak	5	-	-	-	-	-	-	-	1	1	-
		Walking capacity	6MWT	Type of electromechanical device	Exoskeleton	468	13	Random	MD	-8.32 [-27.73 11.08]	0.40	30	Nonsignificant	6	4	-	1	-	-	-	-	-	-	2
		Walking capacity	6MWT	Type of electromechanical device	Mobile device	56	2	Random	MD	20.06 [-39.52 79.63]	0.51	0	Nonsignificant	-	2	-	-	-	-	-	-	-	-	-
		Walking capacity	6MWT	Type of electromechanical device	Ankle device	43	2	Random	MD	49.23 [-17.09 115.55]	0.15	29	Nonsignificant	-	2	-	-	-	-	-	-	-	-	-
	Moucheboeuf (2020) [45]	Walking speed	Gait speed (self-selected gait speed)	Detail of intervention	CT + BWST + RAGT + add vs CT + add	699	11	Random	MD	0.09 [0.03 0.15]	0.002	79	Weak	6	4	-	-	-	-	-	-	-	-	1
		Walking capacity	6MWT	Detail of intervention	CT + BWST + RAGT + add vs CT + add	213	3	Random	MD	22.73 [-16.90 62.35]	0.261	35	Nonsignificant	2	-	-	-	-	-	-	-	-	1	-
		Walking independence	FAC	Detail of intervention	CT + BWST + RAGT + add vs CT + add	639	12	Random	MD	0.51 [0.07 0.95]	0.022	71	Weak	9	-	-	-	-	-	-	-	-	2	1
		Balance capacity	BBS	Detail of intervention	CT + BWST + RAGT + add vs CT + add	230	6	Random	MD	4.16 [2.60 5.71]	0.000	3	Weak	4	-	-	-	-	-	-	-	-	2	-
		Balance capacity	TUG	Detail of intervention	CT + BWST + RAGT + add vs CT + add	77	3	Random	MD	3.20 [-3.20 8.98]	0.277	92	Nonsignificant	1	-	-	-	-	-	-	-	-	2	-
		Walking speed	Gait speed	Detail of intervention, bias	CT + BWST + RAGT + add vs CT + add, low risk of attrition bias	306	6	Random	MD	0.12 [0.09 0.16]	0.000	0	Weak	3	-	-	-	-	-	-	-	-	3	-
		Walking independence	FAC	Detail of intervention, bias	CT + BWST + RAGT + add vs CT + add, low risk of attrition bias	176	5	Random	MD	1.13 [0.73 1.54]	< 0.000	19	Weak	3	-	-	-	-	-	-	-	-	2	-
		Walking speed	Gait speed	Detail of intervention	BWST + RAGT + add vs CT + add	184	7	Random	MD	-0.03 [-0.12 0.04]	0.331	83	Nonsignificant	2	3	-	-	-	-	-	-	-	1	1
		Walking capacity	Gait endurance	Detail of intervention	BWST + RAGT + add vs CT + add	182	6	Random	MD	-23.39 [-65.56 18.77]	0.277	93	Nonsignificant	2	2	-	-	-	-	-	-	-	1	1
		Walking independence	FAC	Detail of intervention	BWST + RAGT + add vs CT + add	117	3	Random	MD	0.00 [-0.10 0.10]	0.954	0	Nonsignificant	2	1	-	-	-	-	-	-	-	-	-
		Balance capacity	BBS	Detail of intervention	BWST + RAGT + add vs CT + add	121	3	Random	MD	0.10 [-1.42 1.62]	0.9	48	Nonsignificant	1	2	-	-	-	-	-	-	-	-	-
		Walking speed	Gait speed	Detail of intervention	CT + BWST + RAGT vs CT + BWST + RAGT + FES	113	4	Random	MD	0.06 [-0.03 0.16]	0.232	0	Nonsignificant	1	-	-	3	-	-	-	-	-	-	-
		Balance capacity	BBS	Detail of intervention	CT + BWST + RAGT vs CT + BWST + RAGT + FES	83	3	Random	MD	0.55 [-3.88 4.98]	0.808	0	Nonsignificant	1	-	-	2	-	-	-	-	-	-	-
		Walking speed	Gait speed	Detail of intervention	BWST + add vs BWST + RAGT + add	55	3	Random	MD	0.04 [-0.13 0.22]	0.63	0	Nonsignificant	-	2	-	1	-	-	-	-	-	-	-
	Postol (2019) [46]	Walking capacity	6MWT	-	-	48	2	Fixed	MD	6.52 [-18.01 31.04]	0.6	0	Nonsignificant	-	2	-	-	-	-	-	-	-	-	-
		Walking speed	10MWT	-	-	35	2	Random	SMD	0.73 [0.03 1.43]	0.04	0	Weak	-	2	-	-	-	-	-	-	-	-	-
		Balance capacity	TUG	-	-	61	3	Random	MD	-1.94 [-13.27 9.38]	0.74	70	Nonsignificant	-	3	-	-	-	-	-	-	-	-	-
		Balance capacity	BBS	-	-	58	3	Fixed	MD	2.74 [1.12 4.36]	0.0009	0	Weak	-	3	-	-	-	-	-	-	-	-	-
	Zheng (2019) [47]	Balance capacity	BBS	-	-	929	23	Random	MD	4.64 [3.22 6.06]	< 0.00001	> 50	Weak	3	5	-	1	-	-	-	-	-	2	12
		Balance capacity	BBS	Different robotic devices	Lokomat	320	8	Random	MD	4.25 [3.11 5.39]	< 0.00001	> 50	Weak	2	-	-	1	-	-	-	-	-	-	5
		Balance capacity	BBS	Different robotic devices	LokoHelp	291	5	Random	MD	7.95 [3.53 12.37]	0.0004	> 50	Weak	-	-	-	-	-	-	-	-	-	-	5
		Balance capacity	BBS	Different robotic devices	Gait Trainer	73	2	Random	MD	5.10 [-4.66 14.87]	0.31	35	Nonsignificant	-	-	-	-	-	-	-	-	-	2	-
		Balance capacity	BBS	Different robotic devices	Other devices	245	8	Random	MD	3.19 [0.25 6.13]	0.03	> 50	Weak	1	4	-	-	-	-	-	-	-	-	3
		Balance capacity	BBS	Robot-assisted therapy combined with another intervention or not	Only robot-assisted therapy	149	5	Random	MD	4.78 [-1.93 11.49]	0.16	> 50	Nonsignificant	1	3	-	-	-	-	-	-	-	-	1
		Balance capacity	BBS	Robot-assisted therapy combined with another intervention or not	RT combined with another intervention	780	18	Random	MD	4.49 [3.41 5.57]	< 0.00001	> 50	Weak	2	2	-	1	-	-	-	-	-	2	11
		Balance capacity	BBS	Differences in the duration of intervention	≤ 4 weeks	294	9	Random	MD	4.24 [1.56 6.93]	0.002	> 50	Weak	2	4	-	1	-	-	-	-	-	1	1
		Balance capacity	BBS	Differences in the duration of intervention	> 4 weeks and < 8 weeks	268	5	Random	MD	4.71 [3.49 5.94]	< 0.00001	40	Weak	-	3	-	-	-	-	-	-	-	2	-
		Balance capacity	BBS	Differences in the duration of intervention	≥ 8 weeks	333	8	Random	MD	4.65 [0.96 8.34]	0.01	> 50	Weak	1	-	-	-	-	-	-	-	-	-	7
		Balance capacity	BBS	Differences in the intensity of intervention	≤ 120 minutes per week	265	6	Random	MD	4.31 [2.70 5.92]	< 0.00001	33	Weak	1	-	-	-	-	-	-	-	-	2	3

		Balance capacity	BBS	Differences in the intensity of intervention	> 120, < 240 minutes per week	558	13	Random	MD	4.71 [2.63 6.79]	< 0.00001	> 50	Weak	2	2	-	1	-	-	-	-	-	-	8
		Balance capacity	BBS	Differences in the intensity of intervention	≥ 240 minutes per week	106	4	Random	MD	4.92 [1.85 8.00]	0.002	> 50	Weak	-	3	-	-	-	-	-	-	-	-	1
		Motor control	FMA-B	-	-	180	3	Fixed	MD	3.57 [2.81 4.34]	< 0.00001	0	Weak	-	-	-	-	-	-	-	-	-	-	3
		Balance capacity	TUG	-	-	68	3	Fixed	MD	-5.60 [-13.69 2.49]	0.18	16	Nonsignificant	1	2	-	-	-	-	-	-	-	-	-
	Bruni (2018) [48]	Walking speed	10MWT, 5MWT, 6MWT, TUG	Type of electromechanical device	End-effector	469	7	Fixed	SMD	0.38 [0.21 0.55]	< 0.05	-	Weak	2	1	-	-	-	-	-	-	-	4	-
		Walking speed	10MWT, 5MWT, 6MWT, TUG	Type of electromechanical device	Exoskeleton	218	6	Fixed	SMD	-0.12 [-0.38 0.14]	0.05 <	-	Nonsignificant	4	1	-	1	-	-	-	-	-	-	-
		Walking speed	10MWT, 5MWT, 6MWT, TUG	Type of electromechanical device, elapsed time from stroke	End-effector, subacute	339	5	Fixed	SMD	0.48 [0.23 0.71]	< 0.05	-	Weak	2	-	-	-	-	-	-	-	-	3	-
		Walking speed	10MWT, 5MWT, 6MWT, TUG	Type of electromechanical device, elapsed time from stroke	Exoskeleton, subacute	181	4	Fixed	SMD	0.12 [-0.18 0.42]	0.05 <	-	Nonsignificant	4	-	-	-	-	-	-	-	-	-	-
		Walking speed	10MWT, 5MWT, 6MWT, TUG	Type of electromechanical device, elapsed time from stroke	End-effector, chronic	130	2	Fixed	SMD	-0.05 [-0.44 0.34]	0.05 <	-	Nonsignificant	-	1	-	-	-	-	-	-	-	1	-
		Walking speed	10MWT, 5MWT, 6MWT, TUG	Type of electromechanical device, elapsed time from stroke	Exoskeleton, chronic	37	2	Fixed	SMD	-0.13 [-0.74 0.48]	0.05 <	-	Nonsignificant	-	1	-	1	-	-	-	-	-	-	-
	Mehrholz (2017a) [50]	Walking independence	FAC, FIM, RMI	-	-	1472	36	Random	OR	1.94 [1.39 2.71]	0.000096	8	Suggestive	12	12	-	2	-	-	1	-	1	4	4
		Walking independence	FAC, FIM, RMI	Follow-up duration	Follow-up	496	6	Random	OR	1.93 [0.72 5.13]	0.19	79	Nonsignificant	3	-	-	1	-	-	-	-	-	2	-
		Walking speed	Walking speed	-	-	985	24	Random	MD	0.04 [0.00 0.09]	0.077	65	Nonsignificant	10	7	-	2	-	-	-	-	-	3	2
		Walking speed	Walking speed	Follow-up duration	Follow-up	578	9	Random	MD	0.07 [-0.05 0.19]	0.26	80	Nonsignificant	3	3	-	1	-	-	-	-	-	1	1
		Walking capacity	6MWT	-	-	594	12	Random	MD	5.84 [-16.73 28.40]	0.61	53	Nonsignificant	3	3	-	2	-	-	-	-	1	1	2
		Walking capacity	6MWT	Follow-up duration	Follow-up	463	7	Random	MD	-0.82 [-32.17 30.53]	0.96	58	Nonsignificant	3	2	-	1	-	-	-	-	-	-	1
		Other measures	Dropout	-	-	1472	36	Random	OR	0.67 [0.43 1.05]	0.081	24	Nonsignificant	13	11	-	2	-	-	-	-	1	5	4
		Other measures	Death	-	-	1472	36	Random	OR	0.00 [-0.01 0.02]	0.77	0	Nonsignificant	13	11	-	2	-	-	-	-	1	5	4
		Walking independence	FAC, FIM, RMI	Trial methods	Adequate sequence generation process	949	20	Random	OR	1.80 [1.06 3.08]	0.031	38	Weak	7	5	-	1	-	-	-	-	1	4	2
		Walking independence	FAC, FIM, RMI	Trial methods	Adequate concealed allocation	831	17	Random	OR	1.87 [1.12 3.12]	0.016	37	Weak	7	2	-	2	-	-	-	-	1	4	1
		Walking independence	FAC, FIM, RMI	Trial methods	Blinded assessors for primary outcome	762	16	Random	OR	1.81 [1.10 2.98]	0.019	31	Weak	9	3	-	-	-	-	-	-	1	1	2
		Walking independence	FAC, FIM, RMI	Trial methods	Without incomplete outcome data	590	14	Random	OR	2.23 [1.16 4.29]	0.017	29	Weak	6	4	-	1	-	-	-	-	1	1	1
		Walking independence	FAC, FIM, RMI	Trial methods	Excluding the largest study	1317	35	Random	OR	1.65 [1.17 2.34]	0.0047	0	Weak	12	11	-	2	-	-	1	-	1	4	4
		Walking independence	FAC, FIM, RMI	Duration after stroke	≤ 3 months	1143	22	Random	OR	1.90 [1.38 2.63]	0.000090	5	Suggestive	13	3	-	-	-	-	1	-	-	2	3
		Walking independence	FAC, FIM, RMI	Duration after stroke	> 3 months	461	16	Random	OR	1.20 [0.40 3.65]	0.74	29	Nonsignificant	1	9	-	2	-	-	-	-	1	2	1
		Walking independence	FAC, FIM, RMI	Ambulatory status at the start of the study	Included independet walkers	500	15	Random	OR	1.38 [0.45 4.20]	0.57	-	Nonsignificant	1	8	-	2	-	-	-	-	1	1	2
		Walking independence	FAC, FIM, RMI	Ambulatory status at the start of the study	Included dependent and independent walkers	340	9	Random	OR	1.90 [1.11 3.25]	0.019	0	Weak	4	1	-	-	-	-	-	-	-	2	2
		Walking independence	FAC, FIM, RMI	Ambulatory status at the start of the study	Included dependent walkers	632	12	Random	OR	1.90 [1.04 3.48]	0.038	45	Weak	7	3	-	-	-	-	1	-	-	1	-
		Walking speed	Walking speed	Ambulatory status at the start of the study	Included independet walkers	317	10	Random	MD	-0.02 [-0.10 0.06]	0.66	59	Nonsignificant	1	5	-	2	-	-	-	-	-	1	1
		Walking speed	Walking speed	Ambulatory status at the start of the study	Included dependent and independent walkers	146	5	Random	MD	0.03 [-0.05 0.11]	0.44	0	Nonsignificant	2	1	-	-	-	-	-	-	-	1	1
		Walking speed	Walking speed	Ambulatory status at the start of the study	Included dependent walkers	522	9	Random	MD	0.10 [0.03 0.17]	0.0062	56	Weak	6	2	-	-	-	-	-	-	-	1	-
		Walking independence	FAC, FIM, RMI	Type of device	End-effector	598	11	Random	OR	1.90 [0.99 3.63]	0.054	50	Nonsignificant	4	2	-	-	-	-	-	-	1	4	-
		Walking independence	FAC, FIM, RMI	Type of device	Exoskeleton	585	16	Random	OR	2.05 [1.21 3.50]	0.0080	0	Weak	7	2	-	2	-	-	1	-	-	-	4
		Walking independence	FAC, FIM, RMI	Type of device	Mobile device	106	3	Random	-	Not applicable	-	-	Nonsignificant	-	3	-	-	-	-	-	-	-	-	-
		Walking independence	FAC, FIM, RMI	Type of device	Ankle device	63	2	Random	-	Not applicable	-	-	Nonsignificant	-	2	-	-	-	-	-	-	-	-	-
		Walking speed	Walking speed	Type of device	End-effector	519	9	Random	MD	0.11 [0.04 0.18]	0.0033	73	Weak	5	1	-	-	-	-	-	-	-	3	-
		Walking speed	Walking speed	Type of device	Exoskeleton	340	12	Random	MD	-0.02 [-0.08 0.04]	0.60	44	Nonsignificant	5	3	-	2	-	-	-	-	-	-	2
		Walking speed	Walking speed	Type of device	Mobile device	106	3	Random	MD	0.02 [-0.11 0.15]	0.78	0	Nonsignificant	-	3	-	-	-	-	-	-	-	-	-
		Walking speed	Walking speed	Type of device	Ankle device	39	1	Random	MD	0.04 [0.01 0.07]	0.022	-	Weak	-	1	-	-	-	-	-	-	-	-	-
		Walking capacity	6MWT	Type of device	End-effector	328	4	Random	MD	27.50 [3.64 51.36]	0.024	4	Weak	2	-	-	-	-	-	-	-	1	1	-
		Walking capacity	6MWT	Type of device	Exoskeleton	186	5	Random	MD	-15.64 [-46.34 15.05]	0.32	51	Nonsignificant	1	-	-	2	-	-	-	-	-	-	2
		Walking capacity	6MWT	Type of device	Mobile device	56	2	Random	MD	20.06 [-39.52 79.63]	0.51	0	Nonsignificant	-	2	-	-	-	-	-	-	-	-	-
		Walking capacity	6MWT	Type of device	Ankle device	24	1	Random	MD	8.00 [-83.03 99.03]	0.86	-	Nonsignificant	-	1	-	-	-	-	-	-	-	-	-
		Walking capacity	6MWT	-	-	594	12	Random	MD	5.84 [-16.73 28.40]	0.61	53	Nonsignificant	3	3	-	2	-	-	-	-	1	1	2
	Hesse (2013) [51]	Walking independence	FAC	-	-	565	9	Random	OR	2.77 [1.16 6.62]	0.02	64	Weak	3	4	-	-	-	-	-	-	-	1	1
	Mehrholz (2012) [52]	Walking independence	BI, FAC, FIM, RMI	Types of electromechanical devices	End-effector	428	7	Fixed	RD	0.09 [0.03 0.15]	0.003	70	Weak	3	1	-	-	-	-	-	-	-	3	-
		Walking independence	BI, FAC, FIM, RMI	Types of electromechanical devices	Exoskeleton	457	11	Fixed	RD	0.01 [-0.02 0.05]	0.41	0	Nonsignificant	4	2	-	2	-	-	-	-	-	-	3
	Ada (2010) [53]	Walking capacity	6MWT	-	4 weeks	88	2	Fixed	MD	35.46 [-12.98 83.91]	0.1514	0.1	Nonsignificant	-	2	-	-	-	-	-	-	-	-	-
	Saragih (2023) [54]	Balance capacity	BBS	-	-	456	15	Random	MD	3.37 [1.29 5.46]	0.02	49.49	Weak	5	3	-	3	-	2	-	-	-	2	-
	Lo (2017) [55]	Walking independence	BI, FAC, FIM	-	-	701	15	Random	SMD	0.17 [-0.15 0.48]	0.31	75	Nonsignificant	6	5	-	1	-	-	1	-	-	2	-
		Walking independence	BI, FAC, FIM	Different impairment levels	Severe	510	10	Random	SMD	0.41 [0.19 0.63]	0.0003	28	Weak	5	2	-	-	-	-	1	-	-	2	-
		Walking independence	BI, FAC, FIM	Detail of intervention	Therapy ratio = 0	207	6	Random	SMD	-0.08 [-0.74 0.58]	0.81	80	Nonsignificant	2	3	-	1	-	-	0	-	-	0	-
		Walking independence	BI, FAC, FIM	Follow-up duration	Follow-up in less than three months	259	5	Random	SMD	-0.33 [-1.31 0.65]	0.51	92	Nonsignificant	2	2	-	-	-	-	1	-	-	-	-
		Walking independence	BI, FAC, FIM	Follow-up duration	Follow-up in more than three months	408	6	Random	SMD	0.30 [-0.05 0.65]	0.1	63	Nonsignificant	2	2	-	-	-	-	3	-	-	-	-
Spinal cord injury	Huang (2024) [56]	Muscle strength	LEMS	Detail of intervention	RAGT	202	3	Random	MD	7.79 [1.59 13.99]	0.01	98	Weak	-	-	-	-	-	-	-	1	-	-	2
	Wan (2024) [57]	Muscle strength	LEMS	-	-	408	11	Random	SMD	0.81 [0.14 1.48]	0.0000	84.5	Weak	1	5	-	-	-	-	1	1	-	1	2
		Muscle strength	LEMS	Type of device	Lokomat	-	10	Random	SMD	0.88 [0.16 1.60]	0.0000	85.5	-	1	4	-	-	-	-	1	1	-	1	2
		Muscle strength	LEMS	Type of device	Exoskeleton assisted walking	-	1	Random	SMD	0.07 [-0.85 0.99]	-	-	-	-	1	-	-	-	-	-	-	-	-	-
		Muscle strength	LEMS	Training intensity	< 6 weeks	-	8	Random	SMD	1.07 [0.23 1.91]	0.0000	88	-	1	3	-	-	-	-	1	1	-	1	2
		Muscle strength	LEMS	Training intensity	> 6 weeks	-	3	Random	SMD	0.08 [-0.51 0.68]	0.969	0.0	-	-	2	-	-	-	-	-	-	-	-	1
		Muscle strength	LEMS	-	OGT	-	4	Random	SMD	0.46 [-0.43 1.35]	0.004	77.8	-	1	1	-	-	-	-	-	-	-	1	1
		Muscle strength	LEMS	-	CPT	-	6	Random	SMD	1.21 [0.09 2.33]	0.0000	88.0	-	-	3	-	-	-	-	1	1	-	-	1
		Muscle strength	LEMS	-	AT	-	1	Random	SMD	0.04 [-0.65 0.72]	-	-	-	-	1	-	-	-	-	-	-	-	-	-
		Muscle strength	LEMS	-	Paraplegia	-	4	Random	SMD	1.37 [-0.11 2.84]	0.0000	91.4	-	1	1	-	-	-	-	-	1	-	1	-
		Muscle strength	LEMS	-	Tetraplegia	-	7	Random	SMD	0.52 [-0.19 1.23]	0.0000	77.7	-	-	4	-	-	-	-	1	-	-	-	2
	Li (2023) [58]	Walking speed	10MWT	-	-	181	6	Fixed	MD	0.01 [-0.06 0.07]	0.77	34	Nonsignificant	4	-	-	-	1	-	-	-	-	1	-
		Walking speed	10MWT	Time since injury	Chronic	51	3	Fixed	MD	-0.06 [-0.15 0.03]	0.23	23	Nonsignificant	1	-	-	-	1	-	-	-	-	1	-
		Walking speed	10MWT	Time since injury	Acure	130	3	Fixed	MD	0.08 [-0.01 0.17]	0.10	0	Nonsignificant	3	-	-	-	-	-	-	-	-	-	-
		Walking capacity	6MWT	-	-	142	5	Fixed	MD	48.55 [29.16 67.94]	< 0.00001	48	Weak	4	-	-	-	1	-	-	-	-	-	-
		Walking capacity	6MWT	Time since injury	Chronic	20	2	Fixed	MD	-53.62 [-145.41 38.17]	0.25	18	Nonsignificant	1	-	-	-	1	-	-	-	-	-	-
		Walking capacity	6MWT	Time since injury	Acure	122	3	Fixed	MD	53.32 [33.49 73.15]	< 0.00001	0	Weak	3	-	-	-	-	-	-	-	-	-	-
		Walking independence	SCIM III	-	-	46	2	Fixed	MD	0.75 [-8.48 9.98]	0.87	0	Nonsignificant	-	-	-	-	-	-	1	1	-	-	-
		Walking independence	SCIM III	Time since injury	Chronic Incomplete SCI	16	1	Fixed	MD	-9.30 [-31.28 12.68]	0.41	-	Nonsignificant	-	-	-	-	-	-	1	-	-	-	-
		Walking independence	SCIM III	Time since injury	Others	30	1	Fixed	MD	2.90 [-7.27 13.07]	0.58	-	Nonsignificant	-	-	-	-	-	-	-	1	-	-	-
		Functional level of mobility	WISCI II	-	-	239	6	Random	MD	1.37 [-0.69 3.42]	0.19	74	Nonsignificant	3	-	-	-	-	-	1	1	-	1	-
		Functional level of mobility	WISCI II	Time since injury	Chronic Incomplete SCI	68	2	Random	MD	-0.71 [-2,77 1.34]	0.50	0	Nonsignificant	-	-	-	-	-	-	1	-	-	1	-
		Functional level of mobility	WISCI II	Time since injury	Acute Incomplete SCI	141	3	Random	MD	3.18 [1.34 5.02]	0.0007	46	Weak	3	-	-	-	-	-	-	-	-	-	-
		Functional level of mobility	WISCI II	Time since injury	Others	30	1	Random	MD	0.10 [-2.81 3.01]	0.95	-	Nonsignificant	-	-	-	-	-	-	-	1	-	-	-
		Muscle strength	LEMS	-	-	230	3	Fixed	MD	4.50 [3.03 5.97]	< 0.00001	25.0	Weak	-	2	-	-	-	-	1	-	-	-	-
		Muscle strength	LEMS	Time since injury	Chronic Incomplete SCI	66	3	Fixed	MD	-1.28 [-5.78 3.21]	0.58	0	Nonsignificant	3	-	-	-	-	-	-	-	-	-	-
		Muscle strength	LEMS	Time since injury	Acute Incomplete SCI	134	1	Fixed	MD	5.22 [3.63 6.80]	< 0.00001	0	Weak	-	-	-	-	-	-	-	1	-	-	-
		Muscle strength	LEMS	Time since injury	Others	30	7	Fixed	MD	4.50 [3.03 5.97]	0.29	-	Nonsignificant	3	2	-	-	-	-	1	1	-	-	-
		Muscle tone	MAS	-	-	110	2	Random	MD	0.51 [-0.00 1.02]	0.05	62	Nonsignificant	1	-	-	-	-	-	-	-	-	1	-
		Muscle tone	MAS	Time since injury	Chronic Incomplete SCI	52	1	Random	MD	0.22 [-0.28 0.72]	0.38	-	Nonsignificant	-	-	-	-	-	-	-	-	-	1	-
		Muscle tone	MAS	Time since injury	Acute Incomplete SCI	58	1	Random	MD	0.75 [0.36 1.14]	0.0002	-	Weak	1	-	-	-	-	-	-	-	-	-	-
	Fang (2020) [59]	Pain	Pain (VAS)	-	-	93	2	Random	MD	-0.890 [-3.086 1.306]	0.427	75.089	Nonsignificant	1	1	-	-	-	-	-	-	-	-	-
		Muscle strength	LEMS	-	-	123	3	Fixed	MD	1.938 [1.143 2.732]	0.000	0	Weak	1	1	-	-	-	-	-	-	-	1	-
		Walking capacity	6MWT	-	-	150	4	Random	MD	55.511 [4.394 106.628]	0.033	0	Weak	1	-	-	-	1	-	-	-	1	1	-
		Walking speed	10MWT	-	-	129	4	Fixed	MD	0.013 [-0.035 0.060]	0.597	0	Nonsignificant	1	1	1	-	1	-	-	-	-	-	-
		Walking independence	WISCI	-	-	127	3	Random	MD	3.383 [-2.567 9.332]	0.265	96.066	Nonsignificant	1	1	-	-	-	-	-	-	-	1	-
		ADL	FIM-Locomotor	-	-	105	2	Random	MD	1.853 [-0.467 4.203]	0.122	94.135	Nonsignificant	1	-	-	-	-	-	-	-	-	1	-
	Nam (2017) [60]	Walking speed	10MWT	Time since injury	Acute (< 6 months)	130	2	Random	WMD	0.08 [-0.00 0.15]	0.05	0	Nonsignificant	2	-	-	-	-	-	-	-	-	-	-
		Walking speed	10MWT	Time since injury	Chronic (>12 months)	197	5	Random	WMD	0.02 [-0.05 0.09]	0.62	56	Nonsignificant	-	-	-	1	-	-	-	-	2	2	-
		Walking speed	10MWT	Time since injury	Unknown	30	1	Random	WMD	0.00 [-0.04 0.04]	1	-	Nonsignificant	-	1	-	-	-	-	-	-	-	-	-
		Walking speed	10MWT	Time since injury	Overall	357	8	Random	WMD	0.02 [-0.02 0.06]	0.25	44	Nonsignificant	2	1	-	1	-	-	-	-	2	2	-
		Walking speed	10MWT	Type of intervention	OGT	130	2	Random	WMD	0.08 [-0.00 0.15]	0.05	0	Nonsignificant	2	-	-	-	-	-	-	-	-	-	-
		Walking speed	10MWT	Type of intervention	Strength	39	2	Random	WMD	-0.00 [-0.04 0.04]	0.98	0	Nonsignificant	-	1	-	1	-	-	-	-	-	-	-
		Walking speed	10MWT	Type of intervention	BWS	64	1	Random	WMD	-0.05 [-0.11 0.01]	0.1	-	Nonsignificant	-	-	-	-	-	-	-	-	-	1	-
		Walking speed	10MWT	Type of intervention	No intervention	124	3	Random	WMD	0.07 [0.01 0.12]	0.01	0	Weak	-	-	-	-	-	-	-	-	2	1	-
		Walking speed	10MWT	Type of intervention	Overall	357	8	Random	WMD	0.02 [-0.02 0.06]	0.25	44	Nonsignificant	2	1	-	1	-	-	-	-	2	2	-
		Walking capacity	2MWT, 6MWT	Time since injury, type of intervention	OGT, acute (< 6 months)	122	2	Random	WMD	45.05 [13.81 76.29]	0.005	0	Weak	2	-	-	-	-	-	-	-	-	-	-
		Walking capacity	2MWT, 6MWT	Time since injury, type of intervention	BWS, chronic (> 12 months)	64	1	Random	WMD	-4.87 [-11.96 2.22]	0.18	-	Nonsignificant	-	-	-	-	-	-	-	-	-	1	-
		Walking capacity	2MWT, 6MWT	Time since injury	No intervention, chronic (> 12 months)	50	1	Random	WMD	-8.30 [-65.16 48.56]	0.77	-	Nonsignificant	-	-	-	-	-	-	-	-	-	1	-
		Walking capacity	2MWT, 6MWT	Time since injury	Overall	236	4	Random	WMD	16.05 [-15.73 47.83]	0.32	69	Nonsignificant	2	-	-	-	-	-	-	-	-	2	-
		Muscle strength	LEMS	Time since injury	Acute (< 6 months)	241	4	Random	WMD	1.47 [-0.15 3.08]	0.08	0	Nonsignificant	3	-	-	-	-	-	-	-	-	1	-
		Muscle strength	LEMS	Time since injury	Chronic (> 12 months)	73	2	Random	WMD	-2.18 [-4.90 0.54]	0.12	0	Nonsignificant	-	1	-	-	-	-	-	-	-	1	-
		Muscle strength	LEMS	Time since injury	Overall	314	6	Random	WMD	0.59 [-1.22 2.40]	0.52	29	Nonsignificant	3	1	-	-	-	-	-	-	-	2	-
		Muscle strength	LEMS	Type of intervention	BWS	94	2	Random	WMD	-0.79 [-3.81 2.23]	0.61	64	Nonsignificant	-	-	-	-	-	-	-	-	-	2	-
		Muscle strength	LEMS	Type of intervention	OGT	211	3	Random	WMD	2.54 [0.11 4.96]	0.04	0	Weak	3	-	-	-	-	-	-	-	-	-	-
		Muscle strength	LEMS	Type of intervention	Strength	9	1	Random	WMD	-0.30 [-7.45 6.85]	0.93	-	Nonsignificant	-	1	-	-	-	-	-	-	-	-	-
		Muscle strength	LEMS	Type of intervention	Overall	314	6	Random	WMD	0.59 [-1.22 2.40]	0.52	29	Nonsignificant	3	1	-	-	-	-	-	-	-	2	-
		ADL, walking independence	FIM-Locomotion, WISCI-II	Time since injury, type of intervention	OGT, acute (< 6 months)	211	3	Random	WMD	0.50 [0.02 0.98]	0.04	67	Weak	3	-	-	-	-	-	-	-	-	-	-
		ADL, walking independence	FIM-Locomotion, WISCI-II	Time since injury, type of intervention	BWS, chronic (> 12 months)	30	1	Random	WMD	0.00 [-0.76 0.76]	1	-	Nonsignificant	-	-	-	-	-	-	-	-	-	1	-

		ADL, walking independence	FIM-Locomotion, WISCI-II	Time since injury, type of intervention	Strength, chronic (> 12 months)	9	1	Random	WMD	0.16 [-1.15 1.48]	0.81	-	Nonsignificant	-	1	-	-	-	-	-	-	-	-	-
		ADL, walking independence	FIM-Locomotion, WISCI-II	Time since injury, type of intervention	Total, overall	250	5	Random	WMD	0.40 [0.02 0.78]	0.04	47	Weak	3	1	-	-	-	-	-	-	-	1	-
		Balance capacity	TUG	Time since injury, type of intervention	No intervention, chronic (> 12 months)	120	3	Random	WMD	9.25 [2.76 15.73]	0.005	74	Weak	-	-	-	-	-	-	-	-	2	1	-
		Muscle tone	MAS	-	-	105	2	Random	WMD	0.48 [-0.50 1.46]	0.34	84	Nonsignificant	1	-	-	-	-	-	-	-	-	1	-
	Mehrholz (2017b) [61]	Walking speed	10MWT, 15MWT	-	-	141	3	Random	WMD	-0.04 [-0.21 0.13]	0.66	57	Nonsignificant	1	-	-	-	-	-	-	-	-	1	1
		Walking capacity	2MWT, 6MWT	-	-	141	3	Random	WMD	-6.14 [-85.92 73.63]	0.88	68	Nonsignificant	1	-	-	-	-	-	-	-	-	1	1
	Cheung (2017) [63]	Walking speed	10MWT	-	-	130	3	Fixed	WMD	-0.08 [-0.17 0.01]	0.100	0	Nonsignificant	3	-	-	-	-	-	-	-	-	-	-
		Walking capacity	6MWT	-	-	122	3	Fixed	WMD	-53.32 [-73.15 -33.48]	< 0.00001	0	Weak	3	-	-	-	-	-	-	-	-	-	-
		Walking independence	WISCI, WISCI-II	-	-	122	3	Fixed	WMD	-3.73 [-4.92 -2.53]	< 0.00001	0	Weak	3	-	-	-	-	-	-	-	-	-	-
		Muscle strength	LEMS	-	-	148	4	Fixed	WMD	-5.00 [-6.56 -3.44]	< 0.00001	0	Weak	3	1	-	-	-	-	-	-	-	-	-
		Muscle tone	MAS, intrinsic stiffness, reflex stiffness	-	-	104	4	Fixed	WMD	-0.01 [-0.49 0.47]	0.960	95	Nonsignificant	3	-	-	-	-	-	-	-	1	-	-
Multiple sclerosis	Yang (2023) [64]	Walking speed	10MWT, 20MWT, T25FW, gait speed (measured using temporal and spatial parameters on a walkway equipped with a camera motion capture system)	Detail of intervention	Without suspension system	32	1	Random	SMD	0.04 [-0.66 0.74]	0.92	-	Nonsignificant	1	-	-	-	-	-	-	-	-	-	-
		Walking speed	10MWT, 20MWT, T25FW, gait speed (measured using temporal and spatial parameters on a walkway equipped with a camera motion capture system)	Detail of intervention	With suspension system	310	9	Random	SMD	0.42 [0.18 0.65]	0.0006	6	Weak	1	5	-	-	2	-	-	1	-	-	-
		Walking speed	10MWT, 20MWT, T25FW, gait speed (measured using temporal and spatial parameters on a walkway equipped with a camera motion capture system)	-	-	342	10	Random	SMD	0.38 [0.15 0.60]	0.0010	6	Weak	2	5	-	-	2	-	-	1	-	-	-
		Walking capacity	6MWT	Detail of intervention	Without suspension system	10	1	Random	SMD	0.16 [-1.11 1.43]	0.8	-	Nonsignificant	-	1	-	-	-	-	-	-	-	-	-
		Walking capacity	6MWT	Detail of intervention	With suspension system	404	11	Random	SMD	0.27 [0.03 0.50]	0.03	25	Weak	1	7	-	1	-	-	-	2	-	-	-
		Walking capacity	6MWT	-	-	414	12	Random	SMD	0.26 [0.04 0.48]	0.02	18	Weak	1	8	-	1	-	-	-	-	-	-	-
		Balance capacity	TUG,RMI	Detail of intervention	Without suspension system	42	2	Random	SMD	-0.20 [-0.82 0.41]	0.52	0	Nonsignificant	1	1	-	-	-	-	-	-	-	-	-
		Balance capacity	TUG,RMI	Detail of intervention	With suspension system	266	6	Random	SMD	-0.40 [-0.64 -0.15]	0.002	0	Weak	1	5	-	-	-	-	-	-	-	-	-
		Balance capacity	TUG,RMI	-	-	308	8	Random	SMD	-0.37 [-0.60 -0.14]	0.002	0	Weak	2	6	-	-	-	-	-	-	-	-	-
		Balance capacity	BBS, Tinetti test	Detail of intervention	With suspension system	325	8	Random	SMD	0.26 [0.04 0.48]	0.02	0	Weak	1	4	-	1	-	-	-	2	-	-	-
		Fatigue	Fatigue severity scale, Modified fatigue imapact scale, Würzburger Erschöpfungsinventar bei Multipler Sklerose scale	Detail of intervention	Without suspension system	32	1	Random	SMD	0.05 [-0.65 0.75]	0.90	-	Nonsignificant	1	-	-	-	-	-	-	-	-	-	-
		Fatigue	Fatigue severity scale, Modified fatigue imapact scale, Würzburger Erschöpfungsinventar bei Multipler Sklerose scale	Detail of intervention	With suspension system	175	6	Random	SMD	-0.30 [-0.54 -0.06]	0.01	0	Weak	-	4	-	1	-	-	-	1	-	-	-
		Fatigue	Fatigue severity scale, Modified fatigue imapact scale, Würzburger Erschöpfungsinventar bei Multipler Sklerose scale	-	-	307	7	Random	SMD	-0.27 [-0.49 -0.04]	0.02	0	Weak	1	4	-	1	-	-	-	1	-	-	-
		Walking speed	10MWT, 20MWT, T25FW, gait speed (measured using temporal and spatial parameters on a walkway equipped with a camera motion capture system)	Follow up period, device type	At the end of the intervention, wearable exoskeltons	-	-	Random	SMD	0.04 [-0.66 0.74]	0.92	-	Nonsignificant	-	-	-	-	-	-	-	-	-	-	-
		Walking speed	10MWT, 20MWT, T25FW, gait speed (measured using temporal and spatial parameters on a walkway equipped with a camera motion capture system)	Follow up period, device type	At the end of the intervention, grounded exoskeltons	-	-	Random	SMD	0.42 [0.18 0.65]	0.0006	6	-	-	-	-	-	-	-	-	-	-	-	-
		Walking speed	10MWT, 20MWT, T25FW, gait speed (measured using temporal and spatial parameters on a walkway equipped with a camera motion capture system)	Follow up period, device type	Total	-	-	Random	SMD	0.38 [0.15 0.60]	0.001	1.1	-	-	-	-	-	-	-	-	-	-	-	-
		Walking speed	10MWT, 20MWT, T25FW, gait speed (measured using temporal and spatial parameters on a walkway equipped with a camera motion capture system)	Follow up period, device type	At follow-up, grounded exoskeletons	194	5	Random	SMD	0.18 [-0.10 0.47]	0.2	0	Nonsignificant	-	4	-	-	-	-	-	-	-	-	1
		Walking capacity	6MWT	Follow up period, device type	At the end of the intervention, wearable exoskeltons	-	-	Random	SMD	0.16 [-1.11 1.43]	0.8	-	Nonsignificant	-	-	-	-	-	-	-	-	-	-	-
		Walking capacity	6MWT	Follow up period, device type	At the end of the intervention, grounded exoskeltons	-	-	Random	SMD	0.27 [0.03 0.50]	0.03	25	-	-	-	-	-	-	-	-	-	-	-	-
		Walking capacity	6MWT	Follow up period, device type	Total	-	-	Random	SMD	0.26 [0.04 0.48]	0.87	18	Nonsignificant	-	-	-	-	-	-	-	-	-	-	-
		Walking capacity	6MWT	Follow up period, device type	At follow-up, grounded exoskeletons	209	5	Random	SMD	0.21 [-0.07 0.48]	0.15	2	Nonsignificant	-	4	-	-	-	-	-	-	-	-	1
		Balance capacity	TUG,RMI	Follow up period, device type	At the end of the intervention, wearable exoskeltons	-	-	Random	SMD	-0.20 [-0.82 0.41]	0.52	0	Nonsignificant	-	-	-	-	-	-	-	-	-	-	-
		Balance capacity	TUG,RMI	Follow up period, device type	At the end of the intervention, grounded exoskeltons	-	-	Random	SMD	-0.40 [-0.64 -0.15]	0.002	0	-	-	-	-	-	-	-	-	-	-	-	-
		Balance capacity	TUG,RMI	Follow up period, device type	Total	-	-	Random	SMD	-0.37 [-0.60 -0.14]	0.002	0	-	-	-	-	-	-	-	-	-	-	-	-
		Balance capacity	TUG,RMI	Follow up period, device type	At follow-up, grounded exoskeletons	260	5	Random	SMD	-0.09 [-0.55 0.36]	0.68	60	Nonsignificant	1	4	-	-	-	-	-	-	-	-	-
		Balance capacity	BBS, Tinetti test	Follow up period, device type	At the end of the intervention, grounded exoskeltons	-	-	Random	SMD	0.26 [0.04 0.48]	0.02	0	Weak	-	-	-	-	-	-	-	-	-	-	-
		Balance capacity	BBS, Tinetti test	Follow up period, device type	At follow-up, grounded exoskeletons	217	6	Random	SMD	-0.16 [-0.56 0.23]	0.42	58	Nonsignificant	1	3	-	-	-	-	-	1	-	-	1
		Fatigue	Fatigue severity scale, Modified fatigue imapact scale, Würzburger Erschöpfungsinventar bei Multipler Sklerose scale	Follow up period, device type	At the end of the intervention, wearable exoskeltons	-	-	Random	SMD	0.05 [-0.65 0.75]	0.9	-	Nonsignificant	-	-	-	-	-	-	-	-	-	-	-
		Fatigue	Fatigue severity scale, Modified fatigue imapact scale, Würzburger Erschöpfungsinventar bei Multipler Sklerose scale	Follow up period, device type	At the end of the intervention, grounded exoskeltons	-	-	Random	SMD	-0.30 [-0.54 -0.06]	0.01	0	-	-	-	-	-	-	-	-	-	-	-	-
		Fatigue	Fatigue severity scale, Modified fatigue imapact scale, Würzburger Erschöpfungsinventar bei Multipler Sklerose scale	Follow up period, device type	Total	-	-	Random	SMD	-0.27 [-0.49 -0.04]	0.02	0	-	-	-	-	-	-	-	-	-	-	-	-
		Fatigue	Fatigue severity scale, Modified fatigue imapact scale, Würzburger Erschöpfungsinventar bei Multipler Sklerose scale	Follow up period, device type	At follow-up, grounded exoskeletons	183	4	Random	SMD	0.02 [-0.27 0.31]	0.87	0	Nonsignificant	-	2	-	-	-	-	-	1	-	-	1
		Comprehensive measures	Multiple Sclerosis Quality of Life-54,36-item short-form health survey, RAND-36	Follow up period, device type	At the end of the intervention, grounded exoskeltons	234	6	Random	SMD	-0.07 [-0.33 0.18]	0.57	0	Nonsignificant	1	2	-	-	-	-	-	2	-	-	1
		Comprehensive measures	Multiple Sclerosis Quality of Life-54,36-item short-form health survey, RAND-36	Follow up period, device type	At follow-up, grounded exoskeltons	215	5	Random	SMD	0.13 [-0.14 0.40]	0.34	0	Nonsignificant	-	3	-	1	-	-	-	-	-	-	1
		Comprehensive measures	Multiple Sclerosis Quality of Life-54,36-item short-form health survey, RAND-36	Follow up period, device type	At the end of the intervention, grounded exoskeltons	234	6	Random	SMD	0.25 [-0.01 0.51]	0.06	0	Nonsignificant	1	2	-	-	-	-	-	2	-	-	1
		Comprehensive measures	Multiple Sclerosis Quality of Life-54,36-item short-form health survey, RAND-36	Follow up period, device type	At follow-up, grounded exoskeltons	215	5	Random	SMD	-0.08 [-0.45 0.29]	0.67	43	Nonsignificant	-	3	-	1	-	-	-	1	-	-	-
		Other measures	EDSS	Follow up period, device type	At the end of the intervention, grounded exoskeltons	144	5	Random	SMD	-0.25 [-0.58 0.08]	0.14	0	Nonsignificant	-	2	-	2	-	-	-	1	-	-	-
		Other measures	EDSS	Follow up period, device type	At follow-up, grounded exoskeltons	114	3	Random	SMD	-0.98 [-2.37 0.41]	0.17	91	Nonsignificant	1	1	-	-	-	-	-	1	-	-	-
		Pain	VAS, Medical Outcomes Study Pain Effects Scale	Follow up period, device type	At the end of the intervention, grounded exoskeltons	92	2	Random	SMD	-0.25 [-0.66 0.17]	0.24	0	Nonsignificant	-	2	-	-	-	-	-	-	-	-	-
		ADL	FIM, mBI	Follow up period, device type	At the end of the intervention, grounded exoskeltons	139	4	Random	SMD	-0.02 [-0.36 0.32]	0.91	0	Nonsignificant	1	2	-	-	-	-	-	1	-	-	-
		ADL	FIM, mBI	Follow up period, device type	At follow-up, grounded exoskeletons	77	2	Random	SMD	0.14 [-0.40 0.68]	0.61	25	Nonsignificant	1	1	-	-	-	-	-	-	-	-	-
		Muscle tone	MAS	Follow up period, device type	At the end of the intervention, grounded exoskeltons	68	2	Random	SMD	-0.31 [-0.79 0.17]	0.2	0	Nonsignificant	-	1	-	-	-	-	-	1	-	-	-
	Yeh (2020) [66]	Walking speed	10MWT, 20MWT, T25FW	Changes from baseline to specific timepoint	Post-treatment	269	8	Random	SMD	-0.02 [-0.36 0.33]	0.93	45	Nonsignificant	1	5	-	2	-	-	-	-	-	-	-
		Walking speed	10MWT, 20MWT, T25FW	Changes from baseline to specific timepoint	Post-treatment at 3 months	152	4	Random	SMD	0.10 [-0.22 0.42]	0.53	0	Nonsignificant	-	4	-	-	-	-	-	-	-	-	-
		Walking speed	10MWT, 20MWT, T25FW	Changes from baseline to specific timepoint, disease condition	Post-treatment, severe	169	4	Random	SMD	0.07 [-0.38 0.52]	0.76	51	Nonsignificant	1	3	-	-	-	-	-	-	-	-	-
		Walking speed	10MWT, 20MWT, T25FW	Changes from baseline to specific timepoint, disease condition	Post-treatment, mild-moderate	100	4	Random	SMD	-0.13 [-0.71 0.45]	0.66	46	Nonsignificant	-	2	-	2	-	-	-	-	-	-	-
		Walking capacity	2MWT, 6MWT	Changes from baseline to specific timepoint	Post-treatment	241	7	Random	SMD	0.26 [-0.08 0.61]	0.13	40	Nonsignificant	1	5	-	1	-	-	-	-	-	-	-
		Walking capacity	2MWT, 6MWT	Changes from baseline to specific timepoint	Post-treatment at 3 months	152	4	Random	SMD	0.17 [-0.25 0.58]	0.43	32	Nonsignificant	-	4	-	-	-	-	-	-	-	-	-
		Walking capacity	2MWT, 6MWT	Changes from baseline to specific timepoint, disease condition	Post-treatment, severe	212	5	Random	SMD	0.24 [-0.11 0.59]	0.19	38	Nonsignificant	1	4	-	-	-	-	-	-	-	-	-
		Walking capacity	2MWT, 6MWT	Changes from baseline to specific timepoint, disease condition	Post-treatment, mild-moderate	29	2	Random	SMD	0.41 [-1.00 1.82]	0.57	70	Nonsignificant	-	1	-	1	-	-	-	-	-	-	-
		Balance capacity	BBS	Changes from baseline to specific timepoint	Post-treatment	215	5	Random	MD	0.05 [-1.81 1.91]	0.96	37	Nonsignificant	-	4	-	1	-	-	-	-	-	-	-
		Balance capacity	BBS	Changes from baseline to specific timepoint	Post-treatment at 3 months	136	3	Random	MD	0.75 [-1.89 3.39]	0.58	0	Nonsignificant	-	3	-	-	-	-	-	-	-	-	-
		Balance capacity	BBS	Changes from baseline to specific timepoint, disease condition	Post-treatment, severe	144	3	Random	MD	0.31 [-2.81 3.42]	0.85	50	Nonsignificant	-	3	-	-	-	-	-	-	-	-	-
		Balance capacity	BBS	Changes from baseline to specific timepoint, disease condition	Post-treatment, mild-moderate	50	2	Random	MD	-0.37 [-4.56 3.82]	0.86	60	Nonsignificant	-	1	-	1	-	-	-	-	-	-	-
		Walking independence, balance capacity	FAC, TUG	Changes from baseline to specific timepoint	Post-treatment	203	5	Random	SMD	0.15 [-0.37 0.67]	0.58	68	Nonsignificant	-	5	-	-	-	-	-	-	-	-	-
		Walking independence, balance capacity	FAC, TUG	Changes from baseline to specific timepoint	Post-treatment at 3 months	152	4	Random	SMD	-0.08 [-0.57 0.41]	0.74	51	Nonsignificant	-	4	-	-	-	-	-	-	-	-	-
		Walking independence, balance capacity	FAC, TUG	Changes from baseline to specific timepoint, disease condition	Post-treatment, severe	187	4	Random	SMD	0.03 [-0.51 0.58]	0.91	70	Nonsignificant	-	4	-	-	-	-	-	-	-	-	-
		Fatigue	Cognitive and physical fatigue score, Fatigue Severity Scale	Changes from baseline to specific timepoint	Post-treatment	242	6	Random	SMD	0.34 [0.02 0.67]	0.04	34	Weak	-	5	-	1	-	-	-	-	-	-	-
		Fatigue	Cognitive and physical fatigue score, Fatigue Severity Scale	Changes from baseline to specific timepoint	Post-treatment at 3 months	128	3	Random	SMD	0.05 [-0.30 0.39]	0.79	0	Nonsignificant	-	3	-	-	-	-	-	-	-	-	-
		Fatigue	Cognitive and physical fatigue score, Fatigue Severity Scale	Changes from baseline to specific timepoint, disease condition	Post-treatment, severe	155	3	Random	SMD	0.44 [-0.06 0.94]	0.09	58	Nonsignificant	-	3	-	-	-	-	-	-	-	-	-
		Fatigue	Cognitive and physical fatigue score, Fatigue Severity Scale	Changes from baseline to specific timepoint, disease condition	Post-treatment, mild-moderate	87	3	Random	SMD	0.22 [-0.26 0.70]	0.37	17	Nonsignificant	-	2	-	1	-	-	-	-	-	-	-
		Muscle tone	MAS, VAS	Changes from baseline to specific timepoint, disease condition	Post-treatment, severe	92	2	Random	SMD	0.70 [0.08 1.33]	0.03	53	Weak	-	2	-	-	-	-	-	-	-	-	-
		Comprehensive measures	Physical Component Summary from the MSQOL-54, RAND-36, SF-36	Changes from baseline to specific timepoint	Post-treatment	166	4	Random	SMD	-0.24 [-0.54 0.07]	0.13	0	Nonsignificant	-	3	-	1	-	-	-	-	-	-	-
		Comprehensive measures	Physical Component Summary from the MSQOL-54, RAND-36, SF-36	Changes from baseline to specific timepoint	Post-treatment at 3 months	136	3	Random	SMD	-0.04 [-0.41 0.33]	0.84	14	Nonsignificant	-	3	-	-	-	-	-	-	-	-	-
		Comprehensive measures	Physical Component Summary from the MSQOL-54, RAND-36, SF-36	Changes from baseline to specific timepoint, disease condition	Post-treatment, severe	144	3	Random	SMD	-0.17 [-0.50 0.16]	0.31	0	Nonsignificant	-	3	-	-	-	-	-	-	-	-	-
		Comprehensive measures	Mental Component Summary from the MSQOL-54, RAND-36, SF-36	Changes from baseline to specific timepoint	Post-treatment	166	4	Random	SMD	0.11 [-0.57 0.79]	0.75	77	Nonsignificant	-	3	-	1	-	-	-	-	-	-	-
		Comprehensive measures	Mental Component Summary from the MSQOL-54, RAND-36, SF-36	Changes from baseline to specific timepoint	Post-treatment at 3 months	142	3	Random	SMD	-0.12 [-0.45 0.21]	0.49	0	Nonsignificant	-	3	-	-	-	-	-	-	-	-	-
		Comprehensive measures	Mental Component Summary from the MSQOL-54, RAND-36, SF-36	Changes from baseline to specific timepoint, disease condition	Post-treatment, severe	144	3	Random	SMD	0.20 [-0.64 1.04]	0.64	83	Nonsignificant	-	3	-	-	-	-	-	-	-	-	-
		Gait index	Stride strength	Changes from baseline to specific timepoint, disease condition	Post-treatment, mild-moderate	67	3	Random	SMD	0.30 [-0.18 0.79]	0.22	0	Nonsignificant	1	1	-	1	-	-	-	-	-	-	-
		Gait index	Double support time	Changes from baseline to specific timepoint, disease condition	Post-treatment, mild-moderate	51	3	Random	SMD	-0.56 [-1.48 0.36]	0.24	59	Nonsignificant	-	1	-	2	-	-	-	-	-	-	-
		Gait index	Cadence	Changes from baseline to specific timepoint, disease condition	Post-treatment, mild-moderate	38	2	Random	SMD	0.15 [-0.51 0.80]	0.66	3	Nonsignificant	-	1	-	1	-	-	-	-	-	-	-
		Walking capacity	RMI	Changes from baseline to specific timepoint, disease condition	Post-treatment, severe	92	2	Random	SMD	0.41 [-0.00 0.83]	0.05	0	Nonsignificant	-	2	-	-	-	-	-	-	-	-	-
		Pain	Bodily pain on SF-36, VAS	Changes from baseline to specific timepoint	Post-treatment	165	3	Random	SMD	0.10 [-0.21 0.40]	0.53	0	Nonsignificant	-	3	-	-	-	-	-	-	-	-	-
		Pain	Bodily pain on SF-36, VAS	Changes from baseline to specific timepoint	Post-treatment at 3 months	112	2	Random	SMD	0.06 [-0.31 0.44]	0.73	0	Nonsignificant	-	2	-	-	-	-	-	-	-	-	-
		Pain	Bodily pain on SF-36, VAS	Changes from baseline to specific timepoint, disease condition	Post-treatment, severe	116	2	Random	SMD	0.10 [-0.27 0.46]	0.61	0	Nonsignificant	-	2	-	-	-	-	-	-	-	-	-
		ADL	BI, FIM	Changes from baseline to specific timepoint, disease condition	Post-treatment, severe	71	2	Random	SMD	0.01 [-0.60 0.62]	0.98	39	Nonsignificant	-	2	-	-	-	-	-	-	-	-	-
		Other measures	EDSS	Changes from baseline to specific timepoint, disease condition	Post-treatment, severe	71	2	Random	SMD	0.22 [-0.25 0.69]	0.37	0	Nonsignificant	-	2	-	-	-	-	-	-	-	-	-
		Other measures	Treatment acceptance: VAS	-	-	68	2	Random	SMD	0.37 [-0.11 0.85]	0.13	0	Nonsignificant	-	2	-	-	-	-	-	-	-	-	-
		Walking speed	10MWT, 20MWT, T25FW	Changes from baseline to specific timepoint, used device	Post-treatment, Lokomat	247	7	Random	SMD	-0.04 [-0.43 0.34]	0.82	51	Nonsignificant	1	5	-	1	-	-	-	-	-	-	-
		Walking speed	10MWT, 20MWT, T25FW	Changes from baseline to specific timepoint, used device	Post-treatment at 3 months, Lokomat	152	4	Random	SMD	0.10 [-0.22 0.42]	0.53	0	Nonsignificant	-	4	-	-	-	-	-	-	-	-	-
		Walking speed	10MWT, 20MWT, T25FW	Changes from baseline to specific timepoint, disease condition, used device	Post-treatment, severe, Lokomat	169	4	Random	SMD	0.07 [-0.38 0.52]	0.76	51	Nonsignificant	1	3	-	-	-	-	-	-	-	-	-
		Walking speed	10MWT, 20MWT, T25FW	Changes from baseline to specific timepoint, disease condition, used device	Post-treatment, mild-moderate, Lokomat	78	3	Random	SMD	-0.24 [-1.01 0.53]	0.54	55	Nonsignificant	-	2	-	1	-	-	-	-	-	-	-
		Walking capacity	2MWT, 6MWT	Changes from baseline to specific timepoint, used device	Post-treatment, Lokomat	198	6	Random	SMD	0.25 [-0.18 0.67]	0.26	49	Nonsignificant	1	4	-	1	-	-	-	-	-	-	-
		Walking capacity	2MWT, 6MWT	Changes from baseline to specific timepoint, used device	Post-treatment at 3 months, Lokomat	152	4	Random	SMD	0.17 [-0.25 0.58]	0.43	32	Nonsignificant	-	4	-	-	-	-	-	-	-	-	-
		Walking capacity	2MWT, 6MWT	Changes from baseline to specific timepoint, disease condition, used device	Post-treatment, severe, Lokomat	169	4	Random	SMD	0.20 [-0.25 0.66]	0.39	52	Nonsignificant	1	3	-	-	-	-	-	-	-	-	-
		Walking capacity	2MWT, 6MWT	Changes from baseline to specific timepoint, disease condition, used device	Post-treatment, mild-moderate, Lokomat	29	2	Random	SMD	0.41 [-1.00 1.82]	0.57	70	Nonsignificant	-	1	-	1	-	-	-	-	-	-	-
		Balance capacity	BBS	Changes from baseline to specific timepoint, used device	Post-treatment, Lokomat	193	4	Random	MD	-0.28 [-2.36 1.81]	0.8	42	Nonsignificant	-	4	-	-	-	-	-	-	-	-	-
		Balance capacity	BBS	Changes from baseline to specific timepoint, used device	Post-treatment at 3 months, Lokomat	136	3	Random	MD	0.75 [-1.89 3.39]	0.58	0	Nonsignificant	-	3	-	-	-	-	-	-	-	-	-
		Balance capacity	BBS	Changes from baseline to specific timepoint, disease condition, used device	Post-treatment, severe, Lokomat	144	3	Random	MD	0.31 [-2.81 3.42]	0.85	50	Nonsignificant	-	3	-	-	-	-	-	-	-	-	-
		Balance capacity	BBS	Changes from baseline to specific timepoint, disease condition, used device	Post-treatment, mild-moderate, Lokomat	28	1	Random	MD	-2.40 [-5.58 1.08]	0.18	-	Nonsignificant	-	1	-	-	-	-	-	-	-	-	-
		Walking independence, balance capacity	FAC, TUG	Changes from baseline to specific timepoint, used device	Post-treatment, Lokomat	160	4	Random	SMD	-0.02 [-0.54 0.51]	0.95	60	Nonsignificant	-	4	-	-	-	-	-	-	-	-	-
		Walking independence, balance capacity	FAC, TUG	Changes from baseline to specific timepoint, used device	Post-treatment at 3 months, Lokomat	152	4	Random	SMD	-0.08 [-0.57 0.41]	0.74	51	Nonsignificant	-	4	-	-	-	-	-	-	-	-	-
		Walking independence, balance capacity	FAC, TUG	Changes from baseline to specific timepoint, disease condition, used device	Post-treatment, severe, Lokomat	144	3	Random	SMD	-0.20 [-0.66 0.26]	0.39	45	Nonsignificant	-	3	-	-	-	-	-	-	-	-	-
		Fatigue	Cognitive and physical fatigue score, Fatigue Severity Scale	Changes from baseline to specific timepoint, used device	Post-treatment, Lokomat	177	4	Random	SMD	0.28 [-0.02 0.58]	0.06	0	Nonsignificant	-	4	-	-	-	-	-	-	-	-	-
		Fatigue	Cognitive and physical fatigue score, Fatigue Severity Scale	Changes from baseline to specific timepoint, used device	Post-treatment at 3 months, Lokomat	128	3	Random	SMD	0.05 [-0.30 0.39]	0.79	0	Nonsignificant	-	3	-	-	-	-	-	-	-	-	-
		Fatigue	Cognitive and physical fatigue score, Fatigue Severity Scale	Changes from baseline to specific timepoint, disease condition, used device	Post-treatment, severe, Lokomat	112	2	Random	SMD	0.19 [-0.18 0.57]	0.3	0	Nonsignificant	-	2	-	-	-	-	-	-	-	-	-
		Fatigue	Cognitive and physical fatigue score, Fatigue Severity Scale	Changes from baseline to specific timepoint, disease condition, used device	Post-treatment, mild-moderate, Lokomat	65	2	Random	SMD	0.44 [-0.06 0.93]	0.08	0	Nonsignificant	-	2	-	-	-	-	-	-	-	-	-
		Muscle tone	MAS, VAS	Changes from baseline to specific timepoint, disease condition, used device	Post-treatment, severe, Lokomat	49	1	Random	MD	0.08 [-0.03 0.19]	0.15	-	Nonsignificant	-	1	-	-	-	-	-	-	-	-	-
		Comprehensive measures	Physical Component Summary from the MSQOL-54, RAND-36, SF-36	Changes from baseline to specific timepoint, used device	Post-treatment, Lokomat	144	3	Random	SMD	-0.17 [-0.50 0.16]	0.31	0	Nonsignificant	-	3	-	-	-	-	-	-	-	-	-
		Comprehensive measures	Physical Component Summary from the MSQOL-54, RAND-36, SF-36	Changes from baseline to specific timepoint, used device	Post-treatment at 3 months, Lokomat	136	3	Random	SMD	-0.04 [-0.41 0.33]	0.84	14	Nonsignificant	-	3	-	-	-	-	-	-	-	-	-
		Comprehensive measures	Physical Component Summary from the MSQOL-54, RAND-36, SF-36	Changes from baseline to specific timepoint, disease condition, used device	Post-treatment, severe, Lokomat	144	3	Random	SMD	-0.17 [-0.50 0.16]	0.31	0	Nonsignificant	-	3	-	-	-	-	-	-	-	-	-
		Comprehensive measures	Mental Component Summary from the MSQOL-54, RAND-36, SF-36	Changes from baseline to specific timepoint, used device	Post-treatment, Lokomat	144	3	Random	SMD	0.20 [-0.64 1.04]	0.64	83	Nonsignificant	-	3	-	-	-	-	-	-	-	-	-

		Comprehensive measures	Mental Component Summary from the MSQOL-54, RAND-36, SF-36	Changes from baseline to specific timepoint, used device	Post-treatment at 3 months, Lokomat	142	3	Random	SMD	-0.12 [-0.45 0.21]	0.49	0	Nonsignificant	-	3	-	-	-	-	-	-	-	-	-
		Comprehensive measures	Mental Component Summary from the MSQOL-54, RAND-36, SF-36	Changes from baseline to specific timepoint, disease condition, used device	Post-treatment, severe, Lokomat	144	3	Random	SMD	0.20 [-0.64 1.04]	0.64	83	Nonsignificant	-	3	-	-	-	-	-	-	-	-	-
		Gait index	Stride strength	Changes from baseline to specific timepoint, used device	Post-treatment, Lokomat	45	2	Random	MD	3.40 [-0.46 7.26]	0.08	0	Nonsignificant	1	1	-	-	-	-	-	-	-	-	-
		Gait index	Double support time	Changes from baseline to specific timepoint, disease condition, used device	Post-treatment, mild-moderate, Lokomat	29	2	Random	MD	-2.00 [-10.24 6.24]	0.63	66	Nonsignificant	-	1	-	1	-	-	-	-	-	-	-
		Gait index	Cadence	Changes from baseline to specific timepoint, disease condition, used device	Post-treatment, mild-moderate, Lokomat	16	1	Random	MD	6.63 [-4.69 17.95]	0.25	-	Nonsignificant	-	1	-	-	-	-	-	-	-	-	-
		Walking capacity	RMI	Changes from baseline to specific timepoint, disease condition, used device	Post-treatment, severe, Lokomat	49	1	Random	MD	0.42 [-0.07 0.91]	0.1	-	Nonsignificant	-	1	-	-	-	-	-	-	-	-	-
		Pain	Bodily pain on SF-36, VAS	Changes from baseline to specific timepoint, used device	Post-treatment, Lokomat	165	3	Random	SMD	0.10 [-0.21 0.40]	0.53	0	Nonsignificant	-	3	-	-	-	-	-	-	-	-	-
		Pain	Bodily pain on SF-36, VAS	Changes from baseline to specific timepoint, used device	Post-treatment at 3 months, Lokomat	112	2	Random	SMD	0.06 [-0.31 0.44]	0.73	0	Nonsignificant	-	2	-	-	-	-	-	-	-	-	-
		Pain	Bodily pain on SF-36, VAS	Changes from baseline to specific timepoint, disease condition, used device	Post-treatment, severe, Lokomat	116	2	Random	SMD	0.10 [-0.27 0.46]	0.61	0	Nonsignificant	-	2	-	-	-	-	-	-	-	-	-
		ADL	BI, FIM	Changes from baseline to specific timepoint, disease condition, used device	Post-treatment, severe, Lokomat	28	1	Random	MD	-0.50 [-1.50 0.50]	0.33	-	Nonsignificant	-	1	-	-	-	-	-	-	-	-	-
		Other measures	EDSS	Changes from baseline to specific timepoint, disease condition, used device	Post-treatment, severe, Lokomat	28	1	Random	MD	-0.02 [-0.29 0.25]	0.88	-	Nonsignificant	-	1	-	-	-	-	-	-	-	-	-
		Other measures	Treatment acceptance: VAS	Used device	Lokomat	68	2	Random	MD	0.65 [-0.18 1.49]	0.13	0	Nonsignificant	-	2	-	-	-	-	-	-	-	-	-
	Sattelmayer (2019) [67]	Walking speed	10MWT, 20MWT, laboratory measures for walking speed evaluation, T25FW	-	-	259	7	Random	SMD	-0.08 [-0.51 0.35]	0.72	57	Nonsignificant	1	4	-	1	-	-	-	-	-	-	1
		Walking capacity	2MWT, 3MWT, 6MWT	-	-	309	8	Random	SMD	-0.24 [-0.67 0.19]	0.27	65	Nonsignificant	1	5	-	1	-	-	-	-	-	-	1
Cerebral palsy	Conner (2022) [68]	Walking capacity	6MWD	-	-	77	4	Random	SMD	0.28 [-0.17 0.73]	0.220	0	Nonsignificant	1	2	-	-	-	-	-	-	-	1	-
		Walking speed	10MWT, free walking speed	-	-	120	5	Random	SMD	0.20 [-0.18 0.57]	0.30	0	Nonsignificant	2	3	-	-	-	-	-	-	-	-	-
		Other measures	GMFM-D	-	-	135	5	Random	SMD	0.05 [-0.29 0.39]	0.77	0	Nonsignificant	-	5	-	-	-	-	-	-	-	-	-
		Other measures	GMFM-E	-	-	135	5	Random	SMD	0.23 [-0.11 0.57]	0.19	0	Nonsignificant	-	4	-	-	-	-	-	-	-	1	-
		Other measures	GMFM-D	Used device	Lokomat	114	4	Random	SMD	0.12 [-0.25 0.49]	0.52	0	Nonsignificant	-	3	-	-	-	-	-	-	-	1	-
		Other measures	GMFM-E	Used device	Lokomat	114	4	Random	SMD	0.28 [-0.10 0.65]	0.15	0	Nonsignificant	-	3	-	-	-	-	-	-	-	1	-
	Cortes-Perez (2022) [69]	Walking speed	10MWT	Detail of intervention	RT vs treadmill training	121	4	Random	SMD	0.25 [-0.15 0.64]	0.22	51.3	Nonsignificant	1	1	-	-	-	-	-	-	-	2	-
		Walking speed	10MWT	Detail of intervention	RT vs CT	58	3	Random	SMD	0.56 [0.03 1.1]	0.04	20.6	Weak	-	3	-	-	-	-	-	-	-	-	-
		Walking speed	10MWT	Detail of intervention	RT + CT vs CT	123	5	Random	SMD	-0.1 [-0.47 0.29]	0.63	0	Nonsignificant	2	1	-	-	-	-	-	1	-	-	1
		Gait index	Step length: 3D gait analysis	Detail of intervention	RT vs treadmill training	81	3	Random	SMD	0.1 [-0.41 0.6]	0.71	0	Nonsignificant	-	1	-	-	-	-	-	-	-	2	-
		Gait index	Step length: 3D gait analysis	Detail of intervention	RT + CT vs CT	79	3	Random	SMD	0.2 [-0.28 0.67]	0.43	0	Nonsignificant	2	-	-	-	-	-	-	-	-	-	1
		Gait index	Step width: 3D gait analysis	Detail of intervention	RT + CT vs CT	61	2	Random	SMD	-0.28 [-0.83 0.28]	0.33	0	Nonsignificant	1	-	-	-	-	-	-	-	-	-	1
		Gait index	Stride length: 3D gait analysis	Detail of intervention	RT vs treadmill training	58	2	Random	SMD	0.17 [-0.46 0.8]	0.6	0	Nonsignificant	-	-	-	-	-	-	-	-	-	2	-
		Walking capacity	6MWT	Detail of intervention	RT vs treadmill training	81	3	Random	SMD	0.1 [-1 1.2]	0.86	0	Nonsignificant	-	1	-	-	-	-	-	-	-	2	-
		Walking capacity	6MWT	Detail of intervention	RT vs CT	60	2	Random	SMD	2.0 [0.36 3.65]	0.017	32.3	Weak	-	1	-	-	-	-	-	-	-	1	-
		Walking capacity	6MWT	Detail of intervention	RT + CT vs CT	149	5	Random	SMD	0.35 [-0.51 1.2]	0.43	0	Nonsignificant	2	-	-	-	-	-	-	1	-	2	-
		Gait index	Cadence: 3D gait analysis	Detail of intervention	RT vs treadmill training	58	2	Random	SMD	0.09 [-0.54 0.72]	0.79	0	Nonsignificant	-	-	-	-	-	-	-	-	-	2	-
		Gait index	Cadence: 3D gait analysis	Detail of intervention	RT vs CT	42	2	Random	SMD	0.21 [-0.4 0.82]	0.5	0	Nonsignificant	-	2	-	-	-	-	-	-	-	-	-
		Gait index	Cadence: 3D gait analysis	Detail of intervention	RT + CT vs CT	44	2	Random	SMD	0.3 [-0.31 0.92]	0.33	0	Nonsignificant	1	-	-	-	-	-	-	-	-	-	1
		Other measures	GMFM-D	Detail of intervention	RT vs treadmill training	81	3	Random	SMD	-0.01 [-0.52 0.5]	0.96	0	Nonsignificant	-	1	-	-	-	-	-	-	-	2	-
		Other measures	GMFM-D	Detail of intervention	RT vs CT	90	3	Random	SMD	-0.12 [-0.61 0.366]	0.62	52	Nonsignificant	-	2	-	-	-	-	-	-	-	1	-
		Other measures	GMFM-D	Detail of intervention	RT + CT vs CT	131	5	Random	SMD	0.22 [-0.13 0.56]	0.21	0	Nonsignificant	1	1	-	-	-	-	-	1	-	2	-
		Other measures	GMFM-E	Detail of intervention	RT vs treadmill training	81	3	Random	SMD	0.11 [-0.39 0.61]	0.66	0	Nonsignificant	-	1	-	-	-	-	-	-	-	2	-
		Other measures	GMFM-E	Detail of intervention	RT vs CT	90	3	Random	SMD	0.63 [0.12 1.13]	0.015	47	Weak	-	2	-	-	-	-	-	-	-	1	-
		Other measures	GMFM-E	Detail of intervention	RT + CT vs CT	131	5	Random	SMD	0.13 [-0.21 0.47]	0.45	0	Nonsignificant	1	1	-	-	-	-	-	1	-	2	-
		Other measures	GMFM-total	Detail of intervention	RT vs treadmill training	63	2	Random	SMD	0.15 [-0.36 0.65]	0.57	0	Nonsignificant	1	1	-	-	-	-	-	-	-	-	-
		Other measures	GMFM-total	Detail of intervention	RT + CT vs CT	154	4	Random	SMD	0.18 [-0.2 0.56]	0.36	0	Nonsignificant	1	1	-	-	-	-	-	-	-	2	-
		ADL	FAQ-WL, WeeFIM	Detail of intervention	RT + CT vs CT	42	2	Random	SMD	0.14 [-0.46 0.75]	0.64	0	Nonsignificant	1	-	-	-	-	-	-	1	-	-	-
	Lefmann (2017) [71]	Walking speed	10MWT, 3D gait	-	-	53	2	Fixed	SMD	0.11 [-0.48 0.70]	0.72	21	Nonsignificant	2	-	-	-	-	-	-	-	-	-	-
Parkinson's disease	Jiang (2024) [72]	Balance capacity	BBS	-	-	267	10	Fixed	MD	2.80 [2.11 3.49]	< 0.00001	39	Weak	1	3	-	1	-	-	-	-	-	1	4
		Balance capacity	ABC	-	-	137	3	Fixed	MD	7.30 [5.08 9.52]	< 0.00001	31	Weak	-	1	-	1	-	-	-	-	-	-	1
		Walking speed	10MWT	-	-	309	8	Fixed	MD	0.06 [0.03 0.10]	0.0009	5	Weak	1	6	-	-	-	-	-	-	-	1	-
		Walking speed	Gait speed	-	-	227	7	Fixed	MD	3.67 [2.58 4.76]	< 0.00001	0	Weak	-	3	-	-	-	-	-	-	-	-	4
		Gait index	Stride length	-	-	166	5	Fixed	MD	5.53 [3.64 7.62]	< 0.00001	0	Weak	-	2	-	-	-	-	-	-	-	1	2
		Gait index	Cadence	-	-	176	5	Fixed	MD	4.52 [0.94 8.10]	0.01	12	Weak	-	3	-	-	-	-	-	-	-	1	1
		Other measures	UPDRS III	-	-	474	11	Fixed	MD	-2.16 [-2.48 -1.83]	< 0.00001	27	Weak	1	5	-	1	-	-	-	-	-	1	3
		Balance capacity	TUG	-	-	404	9	Fixed	MD	-0.56 [-1.12 0.00]	0.05	33	Nonsignificant	-	4	-	1	-	-	-	-	-	-	4
		Walking capacity	6MWT	-	-	204	4	Fixed	MD	13.87 [11.92 15.82]	< 0.00001	21	Weak	-	2	-	-	-	-	-	-	-	1	1
	Xue (2023) [73]	Walking speed	10MWT	-	-	253	6	Random	MD	0.08 [0.01 0.14]	0.03	53	Weak	1	4	-	-	-	-	-	-	-	1	-
		Walking capacity	6MWT	-	-	252	5	Random	MD	42.83 [22.05 63.62]	< 0.00001	97	Weak	-	2	-	-	-	-	-	-	-	1	2
		Balance capacity	BBS	-	-	270	10	Random	MD	3.33 [2.76 3.89]	< 0.00001	77	Weak	1	3	-	1	-	-	-	-	-	1	4
		Balance capacity	TUG	-	-	349	7	Fixed	MD	-1.81 [-2.55 -1.08]	< 0.00001	19	Weak	-	3	-	1	-	-	-	-	-	-	3
		Other measures	UPDRS III	-	-	341	7	Fixed	MD	-3.82 [-4.27 -3.37]	< 0.00001	38	Weak	-	3	-	1	-	-	-	-	-	1	2
		Walking speed	Walking speed	-	-	117	4	Fixed	MD	0.07 [0.04 0.10]	< 0.00001	0	Weak	-	3	-	-	-	-	-	-	-	-	1
		Gait index	Stride length	-	-	96	3	Fixed	MD	9.92 [7.58 12.26]	< 0.00001	58	Weak	-	1	-	1	-	-	-	-	-	1	-
		Gait index	Step length	-	-	107	4	Random	MD	6.01 [1.86 10.17]	0.005	90	Weak	-	2	-	-	-	-	-	-	-	-	2
		Gait index	Cadence	-	-	96	3	Random	MD	6.49 [1.04 11.94]	0.02	38	Weak	-	1	-	1	-	-	-	-	-	1	-
	Alwardat (2018) [74]	Other measures	UPDRS-III	Follow-up duration	Pre-post	286	7	Random	WMD	3.292 [1.378 5.207]	0.000	26.608	Weak	1	4	-	1	-	-	-	-	-	1	-
		Other measures	UPDRS-III	Follow-up duration	1-month follow-up	138	3	Random	WMD	5.512 [2.396 8.629]	0.001	0	Weak	-	2	-	1	-	-	-	-	-	-	-
		Other measures	UPDRS-III	Follow-up duration	3-month follow-up	128	3	Random	WMD	0.522 [-3.535 4.580]	0.801	39.6	Nonsignificant	1	1	-	-	-	-	-	-	-	1	-
		Walking speed	10MWT	Follow-up duration	Pre-post	162	4	Random	WMD	0.166 [0.090 0.243]	0.000	0	Weak	-	3	-	-	-	-	-	-	-	1	-
		Walking speed	10MWT	Follow-up duration	1-month follow-up	72	2	Random	WMD	0.140 [-0.030 0.309]	0.106	0	Nonsignificant	-	2	-	-	-	-	-	-	-	-	-
		Walking speed	10MWT	Follow-up duration	3-month follow-up	90	2	Random	WMD	0.617 [-0.662 1.896]	0.344	39.6	Nonsignificant	-	1	-	-	-	-	-	-	-	1	-
		Balance capacity	ABC	Follow-up duration	Pre-post	100	2	Random	WMD	5.114 [-1.066 11.293]	0.105	0	Nonsignificant	-	1	-	1	-	-	-	-	-	-	-
		Balance capacity	ABC	Follow-up duration	1-month follow-up	100	2	Random	WMD	6.124 [-0.170 12.418]	0.057	0	Nonsignificant	-	1	-	1	-	-	-	-	-	-	-
		Balance capacity	BBS	Follow-up duration	Pre-post	160	3	Random	WMD	3.554 [0.374 6.735]	0.029	56.6	Weak	-	1	-	1	-	-	-	-	-	1	-
		Balance capacity	BBS	Follow-up duration	1-month follow-up	100	2	Random	WMD	3.868 [1.324 6.413]	0.003	85.13	Weak	-	1	-	1	-	-	-	-	-	-	-
		Balance capacity	TUG	Follow-up duration	Pre-post	130	3	Random	WMD	0.522 [-0.913 1.957]	0.476	0	Nonsignificant	-	2	-	1	-	-	-	-	-	-	-
		Balance capacity	TUG	Follow-up duration	1-month follow-up	100	2	Random	WMD	0.467 [-1.513 2.447]	0.644	24.4	Nonsignificant	-	1	-	-	-	-	-	-	-	1	-
		Gait index	Cadence	-	-	118	3	Random	WMD	1.508 [-2.001 5.017]	0.400	0	Nonsignificant	-	2	-	-	-	-	-	-	-	1	-
		Gait index	Stride length	-	-	118	3	Random	WMD	9.283 [7.153 11.414]	0.000	0	Weak	-	2	-	-	-	-	-	-	-	1	-
		Gait index	Stride time	-	-	98	2	Random	WMD	-0.212 [-3.083 2.658]	0.885	0	Nonsignificant	-	1	-	-	-	-	-	-	-	1	-
Neurological disease	Garet (2024) [77]	Muscle tone	MAS	-	-	140	2	Fixed	MD	-0.29 [-0.49 -0.08]	0.45	0	Nonsignificant	1	1	-	-	-	-	-	-	-	-	-

Abbreviations: 10MWT, 10-Meter Walk Test; 15MWT, 15-Meter Walk Test; 20MWT, 20-Meter Walk Test; 2MWT, 2-Minute Walk Test; 3D, 3-dimensional; 3MWT, 3-Minute Walk Test; 5MWT, 5-Meter Walk Test; 6MWT, 6-Minute Walk Test; ADL activities of daily living; AS, Ashworth Scale; BBS, Berg Balance Scale; BI, Barthel Index; BWS, body weight-supported; BWST, body-weight support training; CI, confidence interval; CMSR, Chedoke-McMaster Stages of Recovery; CT, conventional training; EDSS, Expanded Disability Status Scale; EMS, Elderly Mobility Scale; FAC, Functional Ambulation Scale; FAI, Frenchay Activities Index; FAQ-WL, Functional Assessment Questionnaire Walking Scale; FIM, Functional Independence Measure; FMA-B, Fugl-Meyer Assessment-Balance; FMA-LE, Fugl-Meyer Assessment-Lower Extremity; GMFM, Gross Motor Function Measure; IADL, instrumental activities of daily living; LEMS, Lower Extremity Motor Score; MAS, Modified Ashworth Scale; MD, mean difference; MeEAP, Measure of Experiential Aspects of Participation; MI, Motricity Index; MMAS, Modified Motor Assessment Scale; MSQOL-54, Multiple Sclerosis Quality of Life-54; OGT, overground walking training; OR, odds ratio; PASS, Postural Assessment Scale for Stroke; RAGT, robot-assisted gait training; RAND-36, Rand-36 Item Health Survey; RCT, randomized control trial; RD, risk difference; RMA, Rivermead Motor Assessment; RMI, Rivermead Mobility Index; SAS, Stroke Activity Scale; SCIM III, spinal cord independence measure III; SF-36, Short-Form 36; SIS, Stroke Impact Scale; SMD, standardised mean difference; T25FW, Timed 25-Foot Walk; TUG, Timed Up and Go; VAS, Visual Analog Scale; UPDRS, Unified Parkinson's Disease Rating Scale; WISCI, Walking Index for Spinal Cord Injury; WMD, weighted mean difference

Classification of dose matching:

A: Robot-assisted training in addition to conventional therapy was compared with dose-matched conventional therapy.

B: Robot-assisted training was compared with dose-matched conventional therapy.

C: Robot-assisted training was compared with dose matched training consisting of robot-assisted training and conventional therapy.

D: Robot-assisted training was compared with dose-matched other intervention (e.g. body weight support training or elgometer).

E: Robot-assisted training was compared with dose-matched other robot-assisted training.

F: Robot-assisted training was compared with dose-matched training using the same robot on other training mode.

G: Robot-assisted training with other interventions was compared with dose-matched conventional therapy.

H: Robot-assisted training in addition to conventional therapy was compared with conventional therapy. Conventional therapy in both groups was the same amount.

I: Robot-assisted training was compared with minimal or no conventional therapy including sham.

J: Comparisons between more than three groups that included the robot assisted training.
