# Supplementary material for: Effectiveness of Robotic Devices for Medical Rehabilitation: An Umbrella Review
Source: J Clin Med. 2024 Nov 4;13(21):6616. doi: 10.3390/jcm13216616 (PMC11546060; doi:10.3390/jcm13216616)
Supplement: Supplementary file 1 [file jcm-13-06616-s001.zip › Table S2.pdf]

Table S2 Excluded studies and the reasons for exclusion (n = 79)

Study	Reason for exclusion
Alashram AR, Annino G, Padua E. Robot-assisted gait training in individuals with spinal cord injury: A systematic review for the clinical effectiveness of Lokomat. J Clin Neurosci. 2021;91:260–9.	No meta-analysis was conducted for RCTs, while both RCTs and non-RCTs were included.
Alnajjar F, Zaier R, Khalid S, Gochoo M. Trends and Technologies in Rehabilitation of Foot Drop: A Systematic Review. Expert Rev Med Devices. 2021;18(1):31–46.	No meta-analysis was conducted for RCTs, while both RCTs and non-RCTs were included.
Bazan R, Fonseca BHS, Miranda JMA, Nunes HRC, Bazan SGZ, Luvizutto GJ. Effect of Robot-Assisted Training on Unilateral Spatial Neglect After Stroke: Systematic Review and Meta-Analysis of Randomized Controlled Trials. Neurorehabil Neural Repair. 2022;36(8):545-56.	Not focused on physical function.
Bunge LR, Davidson AJ, Helmore BR, Mavrandonis AD, Page TD, Schuster-Bayly TR, et al. Effectiveness of powered exoskeleton use on gait in individuals with cerebral palsy: A systematic review. PLoS One. 2021;16(5):e0252193.	No RCTs were included.
Calabro RS, Cassio A, Mazzoli D, Andrenelli E, Bizzarini E, Campanini I, et al. What does evidence tell us about the use of gait robotic devices in patients with multiple sclerosis? A comprehensive systematic review on functional outcomes and clinical recommendations. Eur J Phys Rehabil Med. 2021;57(5):841–9.	No meta-analysis was conducted for RCTs, while both RCTs and non-RCTs were included.
Campagnini S, Liuzzi P, Mannini A, Riener R, Carrozza MC. Effects of control strategies on gait in robot-assisted post-stroke lower limb rehabilitation: a systematic review. J Neuroeng Rehabil. 2022;19(1):52.	No meta-analysis was conducted for RCTs, while both RCTs and non-RCTs were included.
Carmignano SM, Fundarò C, Bonaiuti D, Calabrò RS, Cassio A, Mazzoli D, et al. Robot-assisted gait training in patients with Parkinson's disease: Implications for clinical practice. A systematic review. NeuroRehabilitation. 2022;51(4):649–63.	No meta-analysis was conducted for RCTs, while both RCTs and non-RCTs were included.
Carpino G, Pezzola A, Urbano M, Guglielmelli E. Assessing Effectiveness and Costs in Robot-Mediated Lower Limbs Rehabilitation: A Meta-Analysis and State of the Art. J Healthc Eng. 2018;2018:7492024.	Focused on cost-benefit analysis.
Carvalho I, Pinto SM, Chagas DDV, Praxedes Dos Santos JL, de Sousa Oliveira T, Batista LA. Robotic Gait Training for Individuals With Cerebral Palsy: A Systematic Review and Meta-Analysis. Arch Phys Med Rehabil. 2017;98(11):2332–44.	Meta-analyses were conducted with a mixture of both RCTs and non-RCTs.
Castelli E, Beretta E, De Tanti A, Arduini F, Biffi E, Colazza A, et al. Robot-assisted rehabilitation for children with neurological disabilities: Results of the Italian consensus conference CICERONE. NeuroRehabilitation. 2022;51(4):665–79.	No RCTs were included.
Chen YP, Howard AM. Effects of robotic therapy on upper-extremity function in children with cerebral palsy: A systematic review. Dev Neurorehabil. 2016;19(1):64–71.	No meta-analysis was conducted for RCTs, while both RCTs and non-RCTs were included.
Chuang YC, Tsai YL, Lin TT, Ou-Yang LJ, Lee YC, Cheng YY, et al. Effects of soft robotic exosuit on ambulation ability in stroke patients: a systematic review. Biomed Eng Online. 2023;22(1):88.	No RCTs were included.
Comino-Suarez N, Moreno JC, Gomez-Soriano J, Megia-Garcia A, Serrano-Munoz D, Taylor J, et al. Transcranial direct current stimulation combined with robotic therapy for upper and lower limb function after stroke: a systematic review and meta-analysis of randomized control trials. J Neuroeng Rehabil. 2021;18(1):148.	Focused on the combination of robotics with transcranial direct current stimulation.
Contreras-Vidal JL, N AB, Brantley J, Cruz-Garza JG, He Y, Manley Q, et al. Powered exoskeletons for bipedal locomotion after spinal cord injury. J Neural Eng. 2016;13(3):031001.	No RCTs were included.
Cumplido C, Delgado E, Ramos J, Puyuelo G, Garces E, Destarac MA, et al. Gait-assisted exoskeletons for children with cerebral palsy or spinal muscular atrophy: A systematic review. NeuroRehabilitation. 2021;49(3):333–48.	No meta-analysis was conducted for RCTs, while both RCTs and non-RCTs were included.
D'Anci KE, Uhl S, Oristaglio J, Sullivan N, Tsou AY. Treatments for Poststroke Motor Deficits and Mood Disorders: A Systematic Review for the 2019 U.S. Department of Veterans Affairs and U.S. Department of Defense Guidelines for Stroke Rehabilitation. Ann Intern Med. 2019;171(12):906–15.	The study design was a review of systematic reviews.
De Miguel-Rubio A, Munoz-Perez L, Alba-Rueda A, Arias-Avila M, Rodrigues-de-Souza DP. A Therapeutic Approach Using the Combined Application of Virtual Reality with Robotics for the Treatment of Patients with Spinal Cord Injury: A Systematic Review. Int J Environ Res Public Health. 2022;19(14)	No RCTs were included.
Dijkers MP, Akers KG, Dieffenbach S, Galen SS. Systematic Reviews of Clinical Benefits of Exoskeleton Use for Gait and Mobility in Neurologic Disorders: A Tertiary Study. Arch Phys Med Rehabil. 2019; doi:10.1016/j.apmr.2019.01.025.	The study design was a review of systematic reviews.
Dominguez-Romero JG, Molina-Aroca A, Moral-Munoz JA, Luque-Moreno C, Lucena-Anton D. Effectiveness of Mechanical Horse-Riding Simulators on Postural Balance in Neurological Rehabilitation: Systematic Review and Meta-Analysis. Int J Environ Res Public Health. 2019;17(1).	The intervention did not include robotic devices.
Dorsch S, Carling C, Cao Z, Fanayan E, Graham PL, McCluskey A, et al. Bobath therapy is inferior to task-specific training and not superior to other interventions in improving arm activity and arm strength outcomes after stroke: a systematic review. Journal of Physiotherapy. 2023;69(1):15–22.	Focused on the efficacy of facilitation techniques.
Duddy D, Doherty R, Connolly J, McNally S, Loughrey J, Faulkner M. The Effects of Powered Exoskeleton Gait Training on Cardiovascular Function and Gait Performance: A Systematic Review. Sensors (Basel). 2021;21(9)	No meta-analysis was conducted for RCTs, while both RCTs and non-RCTs were included.
Duerinck S, Swinnen E, Beyl P, Hagman F, Jonkers I, Vaes P, et al. The added value of an actuated ankle-foot orthosis to restore normal gait function in patients with spinal cord injury: a systematic review. J Rehabil Med. 2012;44(4):299–309.	No RCTs were included.
Federici S, Meloni F, Bracalenti M, De Filippis ML. The effectiveness of powered, active lower limb exoskeletons in neurorehabilitation: A systematic review. NeuroRehabilitation. 2015;37(3):321-40.	No RCTs were included.
Gonzalez A, Garcia L, Kilby J, McNair P. Robotic devices for paediatric rehabilitation: a review of design features. Biomed Eng Online. 2021;20(1):89.	The study design was a review of systematic reviews.
Grampurohit N, Bell A, Duff SV, Mulcahey MJ, Thielen CC, Kaplan G, et al. Highlighting gaps in spinal cord injury research in activity-based interventions for the upper extremity: A scoping review. NeuroRehabilitation. 2021;49(1):23–38.	No meta-analysis was conducted for RCTs, while both RCTs and non-RCTs were included.
Haarman JA, Reenalda J, Buurke JH, van der Kooij H, Rietman JS. The effect of 'device-in-charge' versus 'patient-in-charge' support during robotic gait training on walking ability and balance in chronic stroke survivors: A systematic review. J Rehabil Assist Technol Eng. 2016;3:2055668316676785.	Meta-analyses were conducted with a mixture of both RCTs and non-RCTs.
Hayes SC, James Wilcox CR, Forbes White HS, Vanicek N. The effects of robot assisted gait training on temporal-spatial characteristics of people with spinal cord injuries: A systematic review. J Spinal Cord Med. 2018;41(5):529–43.	No meta-analysis was conducted for RCTs, while both RCTs and non-RCTs were included.
Hayward KS, Kramer SF, Dalton EJ, Hughes GR, Brodtmann A, Churilov L, et al. Timing and Dose of Upper Limb Motor Intervention After Stroke: A Systematic Review. Stroke. 2021;52(11):3706–17.	The intervention did not include robotic devices.
Hill D, Holloway CS, Morgado Ramirez DZ, Smitham P, Pappas Y. What Are User Perspectives of Exoskeleton Technology? A Literature Review. Int J Technol Assess Health Care. 2017;33(2):160–7.	No RCTs were included.
Hohler C, Trigili E, Astarita D, Hermsdorfer J, Jahn K, Krewer C. The efficacy of hybrid neuroprostheses in the rehabilitation of upper limb impairment after stroke, a narrative and systematic review with a meta-analysis. Artif Organs. 2024;48(3):232–53.	Focused on the efficacy of functional electrical stimulation.
Holanda LJ, Silva PMM, Amorim TC, Lacerda MO, Simao CR, Morya E. Robotic assisted gait as a tool for rehabilitation of individuals with spinal cord injury: a systematic review. J Neuroeng Rehabil. 2017;14(1):126.	No RCTs were included.
Khalid S, Alnajjar F, Gochoo M, Renawi A, Shimoda S. Robotic assistive and rehabilitation devices leading to motor recovery in upper limb: a systematic review. Disabil Rehabil Assist Technol. 2021;18(5):658-72.	No RCTs were included.
Kyriakatis GM, Besios T, Lykou PM. The effect of therapeutic exercise on depressive symptoms in people with multiple sclerosis - A systematic review. Mult Scler Relat Disord. 2022;68:104407.	Not focused on physical function.
Lajeunesse V, Vincent C, Routhier F, Careau E, Michaud F. Exoskeletons' design and usefulness evidence according to a systematic review of lower limb exoskeletons used for functional mobility by people with spinal cord injury. Disabil Rehabil Assist Technol. 2016;11(7):535–47.	No RCTs were included.
Lefeber N, Swinnen E, Kerckhofs E. The immediate effects of robot-assistance on energy consumption and cardiorespiratory load during walking compared to walking without robot-assistance: a systematic review. Disabil Rehabil Assist Technol. 2017;12(7):657–71.	No RCTs were included.
Lennon O, Tonellato M, Del Felice A, Di Marco R, Fingleton C, Korik A, et al. A Systematic Review Establishing the Current State-of-the-Art, the Limitations, and the DESIRED Checklist in Studies of Direct Neural Interfacing With Robotic Gait Devices in Stroke Rehabilitation. Front Neurosci. 2020;14:578.	Focused on EMG/EEG utilization during robotic gait training.
Li B, Cunha AB, Lobo MA. Effectiveness and Users' Perceptions of Upper Extremity Exoskeletons and Robot-Assisted Devices in Children with Physical Disabilities: Systematic Review. Phys Occup Ther Pediatr. 2024;44(3):336–79.	No meta-analysis was conducted for RCTs, while both RCTs and non-RCTs were included.
Liu LY, Li Y, Lamontagne A. The effects of error-augmentation versus error-reduction paradigms in robotic therapy to enhance upper extremity performance and recovery post-stroke: a systematic review. J Neuroeng Rehabil. 2018;15(1):65.	No meta-analysis was conducted for RCTs, while both RCTs and non-RCTs were included.
Lo K, Stephenson M, Lockwood C. The economic cost of robotic rehabilitation for adult stroke patients: a systematic review. JBI Database System Rev Implement Rep. 2019;17(4):520–47.	Focused on cost-benefit analysis.

Louie DR, Eng JJ, Lam T, Spinal Cord Injury Research Evidence Research T. Gait speed using powered robotic exoskeletons after spinal cord injury: a systematic review and correlational study. J Neuroeng Rehabil. 2015;12:82.	No RCTs were included.
Louie DR, Eng JJ. Powered robotic exoskeletons in post-stroke rehabilitation of gait: a scoping review. J Neuroeng Rehabil. 2016;13(1):53.	No RCTs were included.
Maranesi E, Riccardi GR, Di Donna V, Di Rosa M, Fabbietti P, Luzi R, et al. Effectiveness of Intervention Based on End-effector Gait Trainer in Older Patients With Stroke: A Systematic Review. J Am Med Dir Assoc. 2020;21(8):1036–44.	No meta-analysis was conducted for RCTs, while both RCTs and non-RCTs were included.
Masiero S, Armani M, Rosati G. Upper-limb robot-assisted therapy in rehabilitation of acute stroke patients: focused review and results of new randomized controlled trial. J Rehabil Res Dev. 2011;48(4):355–66.	The study design was not a systematic review.
Mazzoleni S, Duret C, Grosmaire AG, Battini E. Combining Upper Limb Robotic Rehabilitation with Other Therapeutic Approaches after Stroke: Current Status, Rationale, and Challenges. Biomed Res Int. 2017;2017:8905637.	No RCTs were included.
Mazzucchelli M, Mazzoleni D, Campanini I, Merlo A, Mazzoli D, Melegari C, et al. Evidence-based improvement of gait in post-stroke patients following robot-assisted training: A systematic review. NeuroRehabilitation. 2022;51(4):595–608.	No RCTs were included.
Miguel Cruz A, Rios Rincon AM, Rodriguez Duenas WR, Quiroga Torres DA, Bohorquez-Heredia AF. What does the literature say about using robots on children with disabilities? Disabil Rehabil Assist Technol. 2017;12(5):429–40.	No meta-analysis was conducted for RCTs, while both RCTs and non-RCTs were included.
Morone G, de Sire A, Martino Cinnera A, Paci M, Perrero L, Invernizzi M, et al. Upper Limb Robotic Rehabilitation for Patients with Cervical Spinal Cord Injury: A Comprehensive Review. Brain Sci. 2021;11(12).	No meta-analysis was conducted for RCTs, while both RCTs and non-RCTs were included.
Olmos-Gomez R, Gomez-Conesa A, Calvo-Munoz I, Lopez-Lopez JA. Effects of Robotic-Assisted Gait Training in Children and Adolescents with Cerebral Palsy: A Network Meta-Analysis. J Clin Med. 2021;10(21).	Meta-analyses were conducted with a mixture of both RCTs and non-RCTs.
Onose G, Popescu N, Munteanu C, Ciobanu V, Sporea C, Mirea MD, et al. Mobile Mechatronic/Robotic Orthotic Devices to Assist-Rehabilitate Neuromotor Impairments in the Upper Limb: A Systematic and Synthetic Review. Front Neurosci. 2018;12:577.	No RCTs were included.
Ou CH, Shiue CC, Kuan YC, Liou TH, Chen HC, Kuo TJ. Neuromuscular Electrical Stimulation of Upper Limbs in Patients With Cerebral Palsy: A Systematic Review and Meta-analysis of Randomized Controlled Trials. Am J Phys Med Rehabil. 2023;102(2):151–8.	Focused on the efficancy of functional electrical stimulation.
Payedimarri AB, Ratti M, Rescinito R, Vanhaecht K, Panella M. Effectiveness of Platform-Based Robot-Assisted Rehabilitation for Musculoskeletal or Neurologic Injuries: A Systematic Review. Bioengineering (Basel). 2022;9(4).	No meta-analysis was conducted for RCTs, while both RCTs and non-RCTs were included.
Perez-de la Cruz S. Use of Robotic Devices for Gait Training in Patients Diagnosed with Multiple Sclerosis: Current State of the Art. Sensors (Basel). 2022;22(7).	No meta-analysis was conducted for RCTs, while both RCTs and non-RCTs were included.
Picelli A, Capecci M, Filippetti M, Varalta V, Fonte C, R DIC, et al. Effects of robot-assisted gait training on postural instability in Parkinson's disease: a systematic review. Eur J Phys Rehabil Med. 2021;57(3):472–7.	No meta-analysis was conducted for RCTs, while both RCTs and non-RCTs were included.
Reis SB, Bernardo WM, Oshiro CA, Krebs HI, Conforto AB. Effects of Robotic Therapy Associated With Noninvasive Brain Stimulation on Upper-Limb Rehabilitation After Stroke: Systematic Review and Meta-analysis of Randomized Clinical Trials. Neurorehabil Neural Repair. 2021;35(3):256–66.	Focused on the combination of robotics with transcranial direct current stimulation.
Rodriguez-Fernandez A, Lobo-Prat J, Font-Llagunes JM. Systematic review on wearable lower-limb exoskeletons for gait training in neuromuscular impairments. J Neuroeng Rehabil. 2021;18(1):22.	No meta-analysis was conducted for RCTs, while both RCTs and non-RCTs were included.
Shackleton C, Evans R, Shamley D, West S, Albertus Y. Effectiveness of over-ground robotic locomotor training in improving walking performance, cardiovascular demands, secondary complications and user-satisfaction in individuals with spinal cord injuries: A systematic review. J Rehabil Med. 2019;51(10):723–33.	No RCTs were included.
Shakti D, Mathew L, Kumar N, Kataria C. Effectiveness of robo-assisted lower limb rehabilitation for spastic patients: A systematic review. Biosens Bioelectron. 2018;117:403–15.	No meta-analysis was conducted for RCTs, while both RCTs and non-RCTs were included.
Sheng B, Zhang Y, Meng W, Deng C, Xie S. Bilateral robots for upper-limb stroke rehabilitation: State of the art and future prospects. Med Eng Phys. 2016;38(7):587–606.	No meta-analysis was conducted for RCTs, while both RCTs and non-RCTs were included.
Shi B, Chen X, Yue Z, Yin S, Weng Q, Zhang X, et al. Wearable Ankle Robots in Post-stroke Rehabilitation of Gait: A Systematic Review. Front Neurorobot. 2019;13:63.	No RCTs were included.
Simonetti D, Zollo L, Milighetti S, Miccinilli S, Bravi M, Ranieri F, et al. Literature Review on the Effects of tDCS Coupled with Robotic Therapy in Post Stroke Upper Limb Rehabilitation. Front Hum Neurosci. 2017;11:268.	Focused on the combination of robotics with transcranial direct current stimulation.
Singh H, Unger J, Zariffa J, Pakosh M, Jaglal S, Craven BC, et al. Robot-assisted upper extremity rehabilitation for cervical spinal cord injuries: a systematic scoping review. Disabil Rehabil Assist Technol. 2018;13(7):704–15.	No meta-analysis was conducted for RCTs, while both RCTs and non-RCTs were included.
Smania N, Picelli A, Geroin C, Munari D, Waldner A, Gandolfi M. Robot-assisted gait training in patients with Parkinson's disease. Neurodegenerative Disease Management. 2013;3(4):321–30.	No meta-analysis was conducted for RCTs, while both RCTs and non-RCTs were included.
Stampacchia G, Gazzotti V, Olivieri M, Andrenelli E, Bonaiuti D, Calabro RS, et al. Gait robot-assisted rehabilitation in persons with spinal cord injury: A scoping review. NeuroRehabilitation. 2022;51(4):609–47.	The study design was not a systematic review.
Sung US, Nisa BU, Yotsumoto K, Tanemura R. Effectiveness of robotic-assisted therapy for upper extremity function in children and adolescents with cerebral palsy: a systematic review protocol. BMJ Open. 2021;11(5):e045051.	No meta-analysis was conducted for RCTs, while both RCTs and non-RCTs were included.
Swinnen E, Beckwee D, Meeusen R, Baeyens JP, Kerckhofs E. Does robot-assisted gait rehabilitation improve balance in stroke patients? A systematic review. Top Stroke Rehabil. 2014;21(2):87–100.	No meta-analysis was conducted for RCTs, while both RCTs and non-RCTs were included.
Swinnen E, Duerinck S, Baeyens JP, Meeusen R, Kerckhofs E. Effectiveness of robot-assisted gait training in persons with spinal cord injury: a systematic review. J Rehabil Med. 2010;42(6):520–6.	No meta-analysis was conducted for RCTs, while both RCTs and non-RCTs were included.
Tamburella F, Lorusso M, Tramontano M, Fadlun S, Masciullo M, Scivoletto G. Overground robotic training effects on walking and secondary health conditions in individuals with spinal cord injury: systematic review. J Neuroeng Rehabil. 2022;19(1):27.	No meta-analysis was conducted for RCTs, while both RCTs and non-RCTs were included.
Tan K, Koyama S, Sakurai H, Teranishi T, Kanada Y, Tanabe S. Wearable robotic exoskeleton for gait reconstruction in patients with spinal cord injury: A literature review. J Orthop Translat. 2021;28:55–64.	No meta-analysis was conducted for RCTs, while both RCTs and non-RCTs were included.
Tavaszi I, Nagy AS, Szabo G, Fazekas G. Neglect syndrome in post-stroke conditions: assessment and treatment (scoping review). Int J Rehabil Res. 2021;44(1):3–14.	No meta-analysis was conducted for RCTs, while both RCTs and non-RCTs were included.
van Delden AL, Peper CL, Kwakkel G, Beek PJ. A systematic review of bilateral upper limb training devices for poststroke rehabilitation. Stroke Res Treat. 2012;2012:972069.	No RCTs were included.
Wall A, Borg J, Palmcrantz S. Clinical application of the Hybrid Assistive Limb (HAL) for gait training-a systematic review. Front Syst Neurosci. 2015;9:48.	No meta-analysis was conducted for RCTs, while both RCTs and non-RCTs were included.
Xiong H, Diao X. A review of cable-driven rehabilitation devices. Disabil Rehabil Assist Technol. 2019; doi:10.1080/17483107.2019.1629110.1–13.	No RCTs were included.
Yoo JI, Oh MK, Lee SU, Lee CH. Robot-assisted rehabilitation for total knee or hip replacement surgery patients: A systematic review and meta-analysis. Medicine (Baltimore). 2022;101(40):e30852.	No meta-analysis was conducted for RCTs, while both RCTs and non-RCTs were included.
Yozbatiran N, Francisco GE. Robot-assisted Therapy for the Upper Limb after Cervical Spinal Cord Injury. Phys Med Rehabil Clin N Am. 2019;30(2):367–84.	No meta-analysis was conducted for RCTs, while both RCTs and non-RCTs were included.
Zanatta F, Farhane-Medina NZ, Adorni R, Steca P, Giardini A, D'Addario M, et al. Combining robot-assisted therapy with virtual reality or using it alone? A systematic review on health-related quality of life in neurological patients. Health Qual Life Outcomes. 2023;21(1):18.	No RCTs were included.
Zeng X, Zhu G, Zhang M, Xie SQ. Reviewing Clinical Effectiveness of Active Training Strategies of Platform-Based Ankle Rehabilitation Robots. J Healthc Eng. 2018;2018:2858294.	No RCTs were included.
Zhang K, Chen X, Liu F, Tang H, Wang J, Wen W. System Framework of Robotics in Upper Limb Rehabilitation on Poststroke Motor Recovery. Behav Neurol. 2018;2018:6737056.	No RCTs were included.
Zhang L, Lin F, Sun L, Chen C. Comparison of Efficacy of Lokomat and Wearable Exoskeleton-Assisted Gait Training in People With Spinal Cord Injury: A Systematic Review and Network Meta-Analysis. Front Neurol. 2022;13:772660.	Meta-analyses were conducted with a mixture of both RCTs and non-RCTs.
Zhang M, Davies TC, Xie S. Effectiveness of robot-assisted therapy on ankle rehabilitation-a systematic review. J Neuroeng Rehabil. 2013;10:30.	No RCTs were included.

Abbreviations: EEG, electroencephalography; EMG, electromyography; RCT, randomized controlled trial
