# Supplementary material for: Effectiveness of Robotic Devices for Medical Rehabilitation: An Umbrella Review
Source: J Clin Med. 2024 Nov 4;13(21):6616. doi: 10.3390/jcm13216616 (PMC11546060; doi:10.3390/jcm13216616)
Supplement: Supplementary file 1 [file jcm-13-06616-s001.zip › Table S5.pdf]

Table S5 AMSTAR 2 quality assessment of the included reviews

Disease	Devices	Study	AMSTAR 2 questions Q1	Q2	Q3	Q4	Q5	Q6	Q7	Q8	Q9	Q10	Q11	Q12	Q13	Q14	Q15	Q16	Overall quality
Stroke	Upper-limb device	Carrillo (2023) [16]	Y	PY	Y	N	Y	Y	N	Y	Y	N	N/A	N/A	N	N	N/A	Y	Critically low
		Doumen (2023) [17]	Y	Y	Y	N	Y	Y	N	PY	Y	N	N/A	N/A	N	N	N/A	Y	Critically low
		Gnasso (2023) [18]	Y	Y	Y	N	Y	Y	Y	PY	Y	N	N/A	N/A	Y	N	N/A	Y	Low
		Lee (2023) [19]	Y	Y	Y	N	Y	Y	N	PY	Y	N	Y	Y	Y	Y	Y	Y	Critically low
		Yang (2023) [20]	Y	Y	Y	N	Y	Y	N	PY	Y	N	Y	N	N	N	Y	Y	Critically low
		Moggio (2022) [21]	Y	PY	Y	PY	Y	Y	N	PY	Y	N	N	N	N	N	Y	N	Critically low
		Zhang (2022) [22]	Y	Y	Y	PY	Y	Y	N	PY	Y	N	Y	N	N	N	Y	Y	Critically low
		Zhao (2022) [23]	Y	Y	Y	N	Y	Y	N	Y	Y	Y	Y	N	Y	Y	Y	Y	Critically low
		Rozevink (2021) [24]	Y	Y	Y	N	Y	N	N	PY	Y	N	N	Y	Y	N	N	Y	Critically low
		Wu (2021) [25]	Y	PY	Y	PY	Y	Y	N	PY	Y	N	Y	Y	Y	Y	Y	Y	Low
		Chen (2020) [26]	Y	Y	Y	N	Y	Y	N	PY	Y	N	Y	Y	Y	Y	Y	Y	Critically low
		Chien (2020) [27]	Y	Y	Y	N	Y	Y	N	Y	Y	N	N	N	Y	N	N	Y	Critically low
		Ferreira (2018) [28]	Y	Y	Y	PY	Y	Y	N	Y	Y	N	Y	Y	Y	Y	Y	Y	Low
		Mehrholz (2018) [29]	Y	Y	Y	Y	Y	Y	Y	Y	Y	N	Y	Y	Y	Y	Y	Y	High
		Bertani (2017) [30]	Y	PY	N	PY	N	N	N	N	N	N	Y	N	N	Y	N	Y	Critically low
		Kim (2017) [31]	N	PY	Y	N	N	N	N	PY	Y	N	N/A	N/A	Y	N	N/A	Y	Critically low
		Veerbeek (2017) [32]	Y	PY	Y	N	N	Y	N	PY	Y	N	Y	Y	Y	Y	N	Y	Critically low
		Zhang (2017) [33]	Y	PY	Y	PY	Y	N	N	PY	Y	N	N	Y	Y	N	N	Y	Critically low
		Norouzi (2012) [34]	Y	PY	Y	PY	Y	N	Y	PY	Y	N	Y	Y	Y	N	N	Y	Low
	Lower-limb device	Leow (2023) [35]	Y	Y	Y	N	Y	Y	Y	PY	Y	Y	Y	N	N	Y	Y	Y	Critically low
		Yang (2023) [36]	Y	Y	Y	PY	Y	Y	N	PY	Y	N	N/A	N/A	N	N	N/A	Y	Critically low
		Zhu (2023) [37]	Y	Y	Y	N	Y	Y	N	PY	Y	N	Y	N	Y	Y	Y	Y	Critically low
		Calafiore (2022) [38]	Y	Y	Y	N	N	Y	N	Y	Y	N	Y	N	Y	Y	N	Y	Critically low

		Baronchelli (2021) [39]	Y	Y	Y	N	Y	Y	N	PY	Y	N	N	N	N	N	N	Y	Critically low
		Nedergard (2021) [40]	Y	Y	Y	N	Y	Y	N	Y	Y	N	N	N	Y	N	N	Y	Critically low
		Wang (2021) [41]	Y	Y	Y	PY	N	Y	N	Y	Y	N	N	N	N	Y	Y	Y	Critically low
		Hsu (2020) [42]	Y	PY	Y	PY	Y	N	N	PY	Y	N	Y	Y	Y	Y	N	Y	Critically low
		Maranesi (2020) [43]	Y	PY	Y	N	N	Y	N	PY	Y	N	N/A	N/A	Y	N	N/A	Y	Critically low
		Mehrholz (2020) [44]	Y	Y	Y	Y	Y	Y	Y	Y	Y	N	Y	Y	Y	Y	Y	Y	High
		Moucheboeuf (2020) [45]	Y	Y	Y	PY	Y	Y	N	PY	Y	N	Y	Y	Y	Y	Y	Y	Low
		Postol (2019) [46]	Y	PY	N	N	Y	Y	N	PY	Y	N	Y	N	Y	Y	N	N	Critically low
		Zheng (2019) [47]	Y	PY	Y	N	Y	Y	N	Y	Y	N	Y	Y	Y	Y	Y	Y	Critically low
		Bruni (2018) [48]	Y	N	Y	N	N	N	N	N	N	N	Y	N	N	Y	N	Y	Critically low
		Cho (2018) [49]	Y	PY	Y	N	N	N	N	PY	Y	N	N/A	N/A	N	N	N/A	Y	Critically low
		Mehrholz (2017a) [50]	Y	Y	Y	Y	Y	Y	Y	Y	Y	N	Y	Y	Y	Y	Y	Y	High
		Hesse (2013) [51]	Y	N	Y	Y	Y	Y	N	N	N	N	Y	N	N	Y	N	Y	Critically low
		Mehrholz (2012) [52]	Y	Y	Y	Y	Y	Y	N	Y	Y	N	Y	N	N	Y	Y	N	Critically low
		Ada (2010) [53]	Y	PY	Y	Y	Y	Y	Y	PY	Y	N	Y	Y	Y	Y	N	N	Low
	Upper-/lower-limb device	Saragih (2023) [54]	Y	Y	Y	N	Y	Y	N	Y	Y	N	Y	Y	Y	Y	Y	Y	Critically low
		Lo (2017) [55]	Y	Y	Y	PY	Y	N	Y	PY	Y	N	Y	Y	Y	Y	Y	Y	Moderate
Spinal cord injury	Lower-limb device	Huang (2024) [56]	Y	Y	Y	N	Y	Y	N	PY	Y	Y	Y	N	N	Y	Y	Y	Critically low
		Wan (2024) [57]	Y	Y	Y	PY	Y	Y	N	PY	Y	N	Y	N	N	N	Y	Y	Critically low
		Li (2023) [58]	Y	Y	Y	PY	Y	Y	N	PY	Y	N	Y	N	N	Y	N	Y	Critically low
		Fang (2020) [59]	Y	PY	Y	N	Y	Y	N	Y	Y	N	Y	N	N	N	Y	Y	Critically low
		Nam (2017) [60]	Y	Y	Y	PY	Y	Y	N	PY	Y	N	Y	N	Y	Y	N	Y	Critically low
		Mehrholz (2017b) [61]	Y	PY	Y	Y	Y	Y	Y	PY	Y	N	Y	N	Y	Y	N	Y	Low
		Fisahn (2016) [62]	Y	PY	Y	PY	Y	Y	Y	PY	Y	Y	N/A	N/A	Y	N	N/A	Y	Moderate

	Upper-/lower-limb device	Cheung (2017) [63]	Y	PY	Y	N	Y	Y	Y	PY	Y	N	N	N	N	N	N	Y	Critically low
Multiple sclerosis	Lower-limb device	Yang (2023) [64]	Y	Y	Y	PY	Y	Y	N	PY	Y	N	Y	Y	N	Y	Y	Y	Critically low
		Bowman (2021) [65]	Y	PY	Y	N	Y	Y	N	PY	Y	N	N/A	N/A	Y	N	N/A	Y	Critically low
		Yeh (2020) [66]	Y	Y	Y	PY	Y	Y	N	PY	Y	N	Y	N	N	N	N	Y	Critically low
		Sattelmayer (2019) [67]	Y	PY	Y	PY	Y	Y	Y	Y	Y	N	Y	N	Y	Y	Y	Y	Moderate
Cerebral palsy	Lower-limb device	Conner (2022) [68]	Y	N	Y	N	Y	N	Y	PY	N	N	Y	N	N	Y	N	Y	Critically low
		Cortes-Perez (2022) [69]	Y	Y	Y	Y	Y	Y	N	PY	Y	Y	Y	N	N	Y	Y	Y	Critically low
		Llamas-Ramos (2022) [70]	Y	Y	Y	N	Y	Y	N	PY	Y	N	N/A	N/A	N	N	N/A	Y	Critically low
		Lefmann (2017) [71]	N	PY	Y	N	Y	Y	N	PY	Y	N	Y	N	Y	Y	N	Y	Critically low
Parkinson's disease	Lower-limb device	Jiang (2024) [72]	Y	Y	Y	PY	Y	Y	N	PY	Y	N	Y	N	N	N	Y	Y	Critically low
		Xue (2023) [73]	Y	PY	Y	N	Y	Y	N	PY	Y	N	Y	N	Y	Y	Y	Y	Critically low
		Alwardat (2018) [74]	Y	PY	Y	N	Y	Y	N	PY	Y	N	Y	Y	Y	N	Y	Y	Critically low
Neurological disease	Upper-limb device	Ferreira (2021) [75]	Y	Y	Y	PY	Y	Y	N	Y	Y	N	Y	N	N	N	Y	Y	Critically low
		Dixit (2019) [76]	Y	Y	Y	N	Y	Y	N	PY	Y	N	N/A	N/A	Y	N	N/A	Y	Critically low
	Lower-limb device	Garlet (2024) [77]	Y	Y	Y	PY	Y	Y	N	PY	Y	N	Y	N	N	Y	N	Y	Critically low
		Total ratio of Yes	97%	58%	97%	13%	87%	84%	21%	26%	94%	8%	84%	37%	56%	56%	57%	94%	

Abbreviations: Y = Yes; PY = Partial Yes; N = No; N/A = Not Applicable (no meta-analysis conducted).
The colors in the table refer to the following meanings. Green = No flaw; Yellow: Non-critical flaw; Red = Critical flaw; Blue = Not applicable
