# Supplementary material for: Effectiveness of Robotic Devices for Medical Rehabilitation: An Umbrella Review
Source: J Clin Med. 2024 Nov 4;13(21):6616. doi: 10.3390/jcm13216616 (PMC11546060; doi:10.3390/jcm13216616)
Supplement: Supplementary file 1 [file jcm-13-06616-s001.zip › Table S6.pdf]

Table S6 The list of upper-limb devices included in the RCTs

Device	Stroke	Spinal cord injury	Multiple sclerosis	Cerebral palsy	Total
MIT-Manus/InMotion2	13	0	0	0	13
Bi-Manu-Track	12	0	0	0	12
Armeo Spring	7	1	0	0	8
Amadeo	6	0	0	0	6
NeReBot	5	0	0	0	5
MIME (Mirror Image Movement Enabler)	4	0	0	0	4
ReoGo	4	0	0	0	4
Fourier M2	3	0	0	0	3
Hand Mentor/Hand Mentor Pro	3	0	0	0	3
ARM (Assisted Rehabilitation and Measurement) Guide	2	0	0	0	2
Barrett WAM	2	0	0	0	2
CASIA-ARM	2	0	0	0	2
EXO-UL7	2	0	0	0	2
Gloreha (model unknown)	2	0	0	0	2
Gloreha Sinfonia	2	0	0	0	2
Haptic Master	1	0	1	0	2
InMotion3	2	0	0	0	2
Motore, Amadeo, Pablo, Diego	2	0	0	0	2
Myomo/Myomo e100	2	0	0	0	2
RA-shoulder therapy	2	0	0	0	2
REAplan	1	0	0	1	2
Reha-Slide	2	0	0	0	2
T-WREX	2	0	0	0	2
3D-DHD	1	0	0	0	1
Actuated virtual keypad	1	0	0	0	1
ArmAssist	1	0	0	0	1
Armeo (model unknown)	1	0	0	0	1
Armeo Boom	1	0	0	0	1
ARMin	1	0	0	0	1
ARMin III robot, HandSOME device	1	0	0	0	1
ARMOR	1	0	0	0	1
Armotion	1	0	0	0	1
Bilateral isometric handgrip force training	1	0	0	0	1
Braccio di Ferro	1	0	0	0	1
Brain-Machine-Interface	1	0	0	0	1
Electromechanical device (Exoskeleton, static orthosis, Glove and Actuator modules)	1	0	0	0	1
EMG-driven robot	1	0	0	0	1
Exoskelton for upper limb rehabilitation	1	0	0	0	1
GENTLE/S	1	0	0	0	1
Glove-like hand exoskeleton combined with a FES system	1	0	0	0	1
Gymnasium for Robotic Rehabilitation	1	0	0	0	1
Hand of hope	1	0	0	0	1
Hand robot	1	0	0	0	1
Haptic knob robot	1	0	0	0	1
InMotion Linear Robot	1	0	0	0	1
LOPES II	1	0	0	0	1
MJS614	1	0	0	0	1
MOTOmed viva2	1	0	0	0	1
Myoelectricity-driven hand robot A5	1	0	0	0	1
Neuro-X	1	0	0	0	1
NMES ROBOT	1	0	0	0	1
NMES-robotic hand	1	0	0	0	1
PneuGlove	1	0	0	0	1
Pneu-WREX	1	0	0	0	1
RAPAEL Smart Glove	1	0	0	0	1
Reha-Digit	1	0	0	0	1
ReHapticKnob	1	0	0	0	1
REHAROB	1	0	0	0	1
Reha-Slide duo	1	0	0	0	1
REJOYCE Robot	1	0	0	0	1
Reo Therapy System	1	0	0	0	1
ReoGo-J	1	0	0	0	1
RETRAINER system	1	0	0	0	1
Robotic exoskeleton	1	0	0	0	1
Robotic System	1	0	0	0	1
RT-AAN	1	0	0	0	1
SMART Arm	1	0	0	0	1
UL-EXO7	1	0	0	0	1
VAEDA Glove	1	0	0	0	1
Virtual reality robotic hand	1	0	0	0	1
ZF1000-2EB-T	1	0	0	0	1
Device unknown	2	0	0	0	2

If an RCT used more than one device, the RCT was counted for each device. Thus, the total number of RCTs in this table is larger than the actual number of RCTs.

Abbreviations: RCT, randomized controlled trial
