# Supplementary material for: Effectiveness of Robotic Devices for Medical Rehabilitation: An Umbrella Review
Source: J Clin Med. 2024 Nov 4;13(21):6616. doi: 10.3390/jcm13216616 (PMC11546060; doi:10.3390/jcm13216616)
Supplement: Supplementary file 1 [file jcm-13-06616-s001.zip › Table S7.pdf]

Table S7 The list of lower-limb devices included in the RCTs

Device	Stroke	Spinal cord injury	Multiple sclerosis	Cerebral palsy	Parkinson's disease	Brain injury	Total
Lokomat	40	23	14	11	5	0	93
Gait Trainer	14	1	2	1	3	0	21
G-EO System	6	0	0	0	4	0	10
Erigo	4	0	0	0	0	5	9
Ekso/Ekso GT	3	2	2	1	0	0	8
HAL (Hybrid Assistive Limb)	7	0	0	0	0	0	7
Lokohelp	5	0	0	0	0	0	5
Walkbot/Walkbot-K/Walkbot-S	3	0	0	1	1	0	5
AutoAmbulator	3	0	0	0	0	0	3
BEAR-H1	3	0	0	0	0	0	3
Exowalk	3	0	0	0	0	0	3
RoboGait	2	0	0	1	0	0	3
SMA (Stride Management Assistance)	3	0	0	0	0	0	3
3DCaLT	1	0	0	1	0	0	2
A3 lower limb rehabilitation robot	2	0	0	0	0	0	2
Bionic Leg	2	0	0	0	0	0	2
Flexbot/Flexbot-B	1	0	0	0	1	0	2
GEAR (Gait Exercise Assist Robot)	2	0	0	0	0	0	2
Morning Walk	2	0	0	0	0	0	2
AIDER	0	1	0	0	0	0	1
ALEX II (Active Leg Exoskeleton)	1	0	0	0	0	0	1
Anklebot	1	0	0	0	0	0	1
AVATAR-02 lower limb rehabilitation robot	1	0	0	0	0	0	1
E-Go	1	0	0	0	0	0	1
Exoskeleton Stride Management Assist	0	0	0	0	1	0	1
Gait Master	1	0	0	0	0	0	1
GAR (Gait-Assistance Robot)	1	0	0	0	0	0	1
GEMS (Gait Enhancing and Motivating System)	1	0	0	0	0	0	1
hunova	0	0	0	0	1	0	1
Innowalk Pro	0	0	0	1	0	0	1
I-Walker	1	0	0	0	0	0	1
MBZ-CPM1	0	1	0	0	0	0	1
PAAR	1	0	0	0	0	0	1
Robot AFO	1	0	0	0	0	0	1
Robowalk	1	0	0	0	0	0	1
RT600	0	0	0	1	0	0	1
SUBAR	1	0	0	0	0	0	1
Walkaround	1	0	0	0	0	0	1
YASKAWA lower limb rehabilitation robot	1	0	0	0	0	0	1
Device unknown	3	4	0	0	1	0	8

If an RCT used more than one device, the RCT was counted for each device. Thus, the total number of RCTs in this table is larger than the actual number of RCTs.

Abbreviations: RCT, Randomized controlled trial
