# Supplementary material for: Effectiveness of Robotic Devices for Medical Rehabilitation: An Umbrella Review
Source: J Clin Med. 2024 Nov 4;13(21):6616. doi: 10.3390/jcm13216616 (PMC11546060; doi:10.3390/jcm13216616)
Supplement: Supplementary file 1 [file jcm-13-06616-s001.zip › Table S8.pdf]

Table S8 Outcome measures used in the included studies

Disease	Devices	Study	Primary outcome of interest	Secondary outcomes
Stroke	Upper-limb devices	Carrillo (2023) [16]	· FMA-UE	· AMAT, ARAT, BBT, BI, DN4, dynamometer, FAT, FIM, MBI, MAL, MAS, Mental Composite Score, MI, MRC, NRS, Physical Composite Score, pROM, Reaching Performance Scale, Revised Nottingham Sensation Assessment, surface electromyography, SF-36, Semmes-Weinstein hand monofilament test, Shoulder/Elbow Coordination Index, SIS, The Goal Attainment Scale, The quality of 124 movement section of the MAL, WFMT
		Doumen (2023) [17]	· Motor impairment and disability: ARAT, FMA-UE	
		Gnasso (2023) [18]	· Pain assessment scale: 10-point VAS, DN4, K-SDQ, NRS, Pain-free pROM of the shoulder, The severity degree of the painful shoulder was defined in four grades	
		Lee (2023) [19]	· Upper limb function: ARAT, FAT, FMA-UE, MI, MRC, QuickDASH, SIS	
		Yang (2023) [20]	· Motor function of the upper limb: FMA-UE	· Daily living: FIM, MBI<br>· Motor function: WMFT<br>· Spasticity: MAS
		Moggio (2022) [21]	· Entire upper-extremity function: QuickDASH<br>· Motor damage: MI<br>· Motor recovery: FMA-UE	
		Zhang (2022) [22]	· Motor control function: FMA-UE	· Activity function: ABILHAND, BI, FAT, FIM, MBI, SIS<br>· Motor control function: ARAT, BBT, Grip strength, MAL, MAS, MRC, NHPT, pROM, ROM, WMFT
		Zhao (2022) [23]	· Distal upper-extremity motor function: FMA-WH<br>· Hand dexterity: BBT, NHPT<br>· Spasticity: MAS<br>· Strength: MRC, MI	· ADL: MBI, SIS<br>· Proximal upper-extremity motor function: FMA-SE
		Rozevink (2021) [24]	· Arm function: FMA-UE<br>· Activity level: ARAT, WMFT<br>· Participation level and activity level: MAL	
		Wu (2021) [25]	· Upper-extremity motor impairment: FMA-UE	
		Chen (2020) [26]	· Motor impairment: FMA-UE	· ADL: BI, FIM, MAL, mRS<br>· Social participation: SF-36, SIS<br>· Upper-limb capacity: AMAT, ARAT, BBT, CAHAI, NHPT, WMFT
		Chien (2020) [27]	· Motor control: FMA-UE (FMA-Motor, FMA-SEC, FMA-WH)	· Functional independence: ACTIVLIM questionnaire, BI, FIM (cognition, motor, self-care)<br>· Muscle tone: MAS<br>· QOL: SIS<br>· Upper-extremity performance: ARAT, QuickDASH, WMFT
		Ferreira (2018) [28]	· Motor control: CMSA, FMA-UE, MSS<br>· ROM: pROM with the assistance of WAM or therapist for the elbows, total pROM<br>· Spasticity: MAS<br>· Strength: grip strength, maximum resistive force with WAM control program, MI, MMT, motor power range, MPS, MRC<br>· Pain: CMSA Pain Inventory Scale range 1–7, Pain Scale of FM, VAS	
		Mehrholz (2018) [29]	· ADL: ABILHAND, BI, FAT, FIM, MBI, SIS 2.0, SIS 3.0 (motor function, social participation)	· Acceptability: dropouts during the intervention period<br>· Motor function: CMSA, FMA-UE, WMFT<br>· Muscle strength: grip force, MI, MRC
		Bertani (2017) [30]	· Impairment in motor function: FMA-UE<br>· Muscle tone: MAS	· ADL: FIM, MAL
		Kim (2017) [31]	· Upper-extremity function assessment: BBT, FIM, FMA-UE, MAL, MAS, MI, pROM, VAS, WMF	
		Veerbeek (2017) [32]	· Basic ADL: BI, FIM, mRS<br>· Motor control: CMSA, FMA-UE (FMA-SEC, FMA-WH)<br>· Muscle strength: MI (Arm subscale), MPS, MRC<br>· Muscle tone: AS, MAS<br>· Upper-limb capacity: AMAT, ARAT, BBT, WMFT	
		Zhang (2017) [33]	· Motor recovery: FMA-UE	
		Norouzi (2012) [34]	· Upper-limb functional or motor recovery: FIM, FMA-UE, MPS, MSS	
	Lower-limb devices	Leow (2023) [35]	· Walking ability: FAC<br>· Walking endurance: 2MWT, 6MWT<br>· Walking speed: 10MWT, 5MWT	
		Yang (2023) [36]	· Balance function: BBS, TUG<br>· Gait performance: 10MWT, consistency of intralimb movements on the impaired limb, FAC, Functional Gait Assessment, gait speed, impaired single-limb stance time, Rivermead Visual Gait Assessment, step length of the affected limb<br>· Physical endurance: 6MWT, number of steps taken during walking	
		Zhu (2023) [37]	· Balance: BBS<br>· Walking ability: FAC	· Gait assessment: Cadence<br>· Walking endurance: 6MWT<br>· Walking speed: 10MWT
		Calafiore (2022) [38]	· Qualitative gait assessment: FAC, mEFAP, MM, WHS<br>· Quantitative gait scale: 10MWT, 2MWT, 6MWT, TUG<br>· Quantitative parameters: maximum walking speed, stance duration and single support time for both legs, stride duration cadence, walking distance, walking speed	
		Baronchelli (2021) [39]	· Balance: BBS, TUG, RMI, mEFAP, MM, PASS, POMA-B, RMA, SPPB	
		Nedergard (2021) [40]	· Parameters related to temporal and spatial information based on kinematics and kinetics: cadence, spatial symmetry, step length, stride length, temporal symmetry, walking speed	
		Wang (2021) [41]	· Balance function: BBS	
		Hsu (2020) [42]	· ADL: ADL-IADL, FAI, FIM, SAS, SIS<br>· Balance capacity: BBS, Brunel Balance Assessment, PASS, TUG<br>· Endurance/ fitness: 2MWT, 5MWT, 6MWT, peak VO_2, VO_2 during the 5MWT<br>· Mobility capacity: EU walking, EMS, FAC, Motor Assessment Scale, MeEAP, MMAS, RMA, RMI<br>· Motor impairment: CMSR, FMA-LE, MI<br>· Walking speed: 10MWT, 2MWT, 5MWT	

		Maranesi (2020) [43]	· Gait and gait-related function: 6MWT, BBS, BI, FAC, MI, RMI	
		Mehrholz (2020) [44]	· Independent walking: BI, FAC, FIM, RMI	· Adverse outcomes: death from all causes until the end of the intervention phase, lost to study during the intervention phase, dropouts<br>· Walking capacity: 6MWT<br>· Walking speed
		Mouchebouef (2020) [45]	· BBS<br>· FAC<br>· Gait endurance<br>· Gait speed (self-selected gait speed)<br>· TUG	
		Postol (2019) [46]	· Neuromotor function related to the lower limbs: 10MWT, 6MWT, BBS, TUG, Functional Reach Test, Sit-to-Stand Test	· Acceptability<br>· Mood<br>· QOL<br>· Safety
		Zheng (2019) [47]	· Balance function: BBS, FMA-B, TUG, ABC, dynamic balance time, dynamic balance trip, Standing Forward Reach Test, Static Balance Test, Trunk Impairment Scale	
		Bruni (2018) [48]	· Walking speed: 10MWT, 5MWT, 6MWT, TUG	· Ambulation ability: FAC
		Cho (2018) [49]	· Gait and gait-related function: 6MWT, BBS, BI, FAC, FIM, RMI, TUG, TWT	
		Mehrholz (2017a) [50]	· Independent walking: BI, FAC, FIM, RMI	· Adverse outcomes: death from all causes until the end of the intervention phase, lost to study during the intervention phase, dropouts<br>· Walking capacity: 6MWT<br>· Walking speed
		Hesse (2013) [51]	· Ability to walk independently: BI, FAC, FIM, RMI	
		Mehrholz (2012) [52]	· Walking independence: BI, FAC, FIM, RMI	
		Ada (2010) [53]	· Independent walking: 15 m continuously with no aids, FAC	· Walking capacity: 2MWT, 6MWT<br>· Walking speed: 10MWT, 5MWT
	Upper-/lower-limb devices	Saragih (2023) [54]	· ADL: BI, Korean MBI, MBI, SIS<br>· Balance: BBS<br>· Cognitive function: Addenbrooke cognitive examination-revised, FIM, MMSE, participants cognitive function, SIS<br>· Motor activity: MAL<br>· Sensorimotor function: FMA-lower, FMA-upper<br>· Somatosensory function: California functional evaluation, DN4, pain scale, VAS	
		Lo (2017) [55]	· Lower-limb walking: BI, FAC, FIM<br>· Upper-limb movement: FMA-UE, MI	· ADL: BI, FIM
Spinal cord injury	Lower-limb devices	Huang (2024) [56]	· LEMS, MBI, walking speed	
		Wan (2024) [57]	· Lower extremity strength: LEMS<br>· Cardiopulmonary function: anaerobic threshold, FEV1, FVC, maximal voluntary ventilation, maximum heart rate, metabolic equivalent of energy, PEF, VO2max, VO2peak	
		Li (2023) [58]	· Cardiopulmonary fitness : peak oxygen consumption, FEV1, FVC, peak, PEF<br>· Endurance: 6MWT<br>· Functional independence: SCIM III<br>· Functional level of mobility: WISCI II<br>· Spasticity: MAS, intrinsic & reflex stiffness, Spinal Cord Assessment Tools for Spastic reflexes<br>· Strength: LEMS, Ambulatory Motor Index, MRC<br>· Walking speed: 10MWT, TUG	· ADL: FIM-Locomotor,<br>· Ankle kinematic and kinetic assessments<br>· Balance: BBS, modified functional reach test<br>· Gait characteristics<br>· Pain: Patient Global Impression of Change, VAS<br>· Spasticity: MAS
		Fang (2020) [59]	· Pain: VAS<br>· Spaticity: AS, MAS	· Motor score for the lower limbs: LEMS<br>· Physical functions: FIM-Locomotor<br>· Walking distance: 6MWT<br>· Walking independence: WISCI<br>· Walking speed: 10MWT
		Nam (2017) [60]	· Balance: TUG<br>· Functional level of mobility and independence: FIM-Locomotion, WISCI-II<br>· Gait capacity: 2MWT, 6MWT<br>· Gait speed: 10MWT<br>· Leg strength: LEMS<br>· Spasticity: MAS	
		Mehrholz (2017b) [61]	· Walking speed: 10MWT, 15MWT<br>· Walking distance: 2MWT, 6MWT	
		Fisahn (2016) [62]	· Functional improvement: FIM-Locomotor, SCIM, WISCI I, WISCI II<br>· Walking speed: 10MWT, 6MWT	· Lower-extremity motor function: LEMS
	Upper-/lower-limb devices	Cheung (2017) [63]	· Walking endurance: 6MWT<br>· Walking independence: WISCI, WISCI II<br>· Walking speed: 10MWT<br>· ARAT	· LEMS<br>· Lower-limb muscle spasticity: AS, intrinsic stiffness (amount of torque per unit change in ankle position), reflex stiffness (amount of torque per unit change in perturbation velocity)<br>· GRASSP<br>· Maximum oxygen consumption during a functional task<br>· Maximum oxygen consumption during a nonfunctional task
Multiple sclerosis	Lower-limb devices	Yang (2023) [64]	· Walking velocity: 10MWT, 20MWT, gait speed (measured using temporal and spatial parameters on a walkway equipped with a camera motion capture system), T25FW<br>· Walking endurance: 2MWT, 3MWT, 6MWT	· ADL: FIM, MBI<br>· Balance: BBS, Tinetti Test<br>· Disease progression: EDSS<br>· Fatigue: Fatigue severity scale, Modified fatigue impact scale, Würzburger Erschöpfungsinventar bei Multipler Sklerose scale<br>· Mobility: RMI, TUG<br>· Pain: Medical Outcomes Study Pain Effects Scale, VAS<br>· QOL: MSQOL-54, RAND-36, SF-36<br>· Spasticity: MAS
		Bowman (2021) [65]	· Balance: ABC, BBS, SOT, TUG<br>· Gait: 10MWT, 2MWT, 20MWT, 3MWS, 6MWT, cadence, double support time, FAC, speed, step length, T25FW	

		Yeh (2020) [66]	· Ambulation capability: FAC, TUG<br>· Balance: BBS<br>· Gait endurance: 2MWT, 6MWT<br>· Gait speed: 10MWT, 20MWT, T25FW<br>· Gait parameters: Cadence, double support time, stride length	· ADL: BI, FIM<br>· EDSS<br>· Fatigue: cognitive and physical fatigue score, Fatigue Severity Scale<br>· Global mobility: RMI<br>· Pain: "bodily pain" on SF-36, VAS<br>· QOL: MSQOL-54, RAND-36, SF-36<br>· Spasticity: MAS, VAS<br>· Treatment acceptance: VAS
		Sattelmayer (2019) [67]	· Walking performance over short distances: 10MWT, 20MWT, laboratory measures for walking speed evaluation, T25FW	· Walking performance over long distances: 2MWT, 3MWT, 6MWT<br>· Disease-related mobility impairment: EDSS
Cerebral palsy	Lower-limb devices	Conner (2022) [68]	· 6MWT<br>· GMFM-D (standing)<br>· GMFM-E (jumping, running, walking)<br>· Walking speed: 10MWT, free walking speed	
		Cortes-Perez (2022) [69]	· 3D gait analysis: cadence, step length, step width, stride length<br>· Funcrtional independence: FAQ-WL, WeeFIM<br>· Standing ability: GMFM-D<br>· Walking distance: 6MWT<br>· Walking, running and jumping ability: GMFM- E<br>· Walking speed: 10MWT	
		Llamas-Ramos（2022）[70]	· Motor dysfunction: balance, functional independence, gait patterns, lower-limb kinematics, muscle strength, peripheral O2 saturation, postural and locomotor functions, running and climbing activities, standing activity, upper-body control, walking ability, walking speed	
		Lefmann (2017) [71]	· Gait speed: 10MWT, 3D gait<br>· Function: WeeFIM<br>· Gait assist: FAC<br>· Gait distance: 6MWT<br>· GMFM-D (standing)<br>· GMFM-E (walking)<br>· Participation: COPM	
Parkinson's disease	Lower-limb devices	Jiang (2024) [72]	· Motor dysfunction: 10MWT, 6MWT, ABC, BBS, cadence, gait speed, stride length, TUG, UPDRS III	
		Xue (2023) [73]	· 10MWT, 6MWT, BBS, TUG, UPDRS III	· Cadence, Step length, Stride length, walking speed
		Alwardat (2018) [74]	· Balance capacity: ABC, BBS, TUG<br>· Gait speed: 10MWT<br>· Motor performance and function: UPDRS-III<br>· Spatiotemporal gait characteristics: gait characteristics analysis (cadence, stride length, time)	
Neurological disease	Upper-limb devices	Ferreira (2021) [75]	· Participation: Life Habits, SIS<br>· QOL: SF-36, SIS	
		Dixit (2019) [76]	· Arm motor section: FMA-UE<br>· Functional independence score: FIM<br>· Jebsen-Taylor Hand Function Test<br>· MI<br>· QOL: QUEST<br>· UPDRS	
	Lower-limb devices	Garlet (2024) [77]	· Cardiac parameters (blood pressure, heart rate, oxygen saturation)	· Muscle strength: MRC, dynamometry<br>· Spasticity: MAS<br>· Coma state and level of consciousness: Coma Recovery Scale-Revised<br>· Functional measurement: FM, FIM<br>· Gait and balance: Tinetti scale

The underline shows outcome measures that were used for meta-analysis.

Abbreviations: 10MWT, 10-Meter Walk Test; 15MWT, 15-Meter Walk Test; 20MWT, 20-Meter Walk Test; 2MWT, 2-Minute Walk Test; 3D, 3-dimensional; 3MWS, 3-Minute Walking Speed; 3MWT, 3-Minute Walk Test; 5MWT, 5-Meter Walk Test; 6MWT, 6-Minute Walk Test; ABC, Activities-specific Balance Confidence; ABILHAND, A Measure of Manual Ability for People with Upper Limb Impairment; ACTIVLIM, Activity Limitations for Patients with Upper and/or Lower Limb Impairments; ADL, activities of daily living; AMAT, Arm Motor Ability Test; ARAT, Action Research Arm Test; AS, Ashworth Scale; BBS, Berg Balance Scale; BBT, Box and Block Test; BI, Barthel Index; CAHAI, Chedoke Arm and Hand Activity Inventory; CMSA, Chedoke McMaster Stroke Assessment; CMSR, Chedoke-McMaster Stages of Recovery; COPM, Canadian Occupational Performance Measure; DN4, Neuropathic Pain Diagnostic Questionnaire; EDSS, Expanded Disability Status Scale; EMS, Elderly Mobility Scale; FAC, Functional Ambulation Scale; FAI, Frenchay Activities Index; FAQ-WL, Functional Assessment Questionnaire Walking Scale; FAT, Frenchay Arm Test; FEV1, forced expiratory volume in the first 1 second; FIM, Functional Independence Measure; FM, Fugl-Meyer; FMA-B, Fugl-Meyer Assessment-Balance; FMA-LE, Fugl-Meyer Assessment-Lower Extremity; FMA-SE, Fugl-Meyer Assessment-Shoulder and Elbow; FMA-SEC, Fugl-Meyer Assessment-Shoulder/Elbow and Coordination; FMA-UE, Fugl-Meyer Assessment-Upper Extremity; FMA-WH, Fugl-Meyer Assessment-Wrist and Hand; FVC, forced vital capacity; GMFM, Gross Motor Function Measure; GRASSP, Graded Redefined Assessment of Strength, Sensibility and Prehension; IADL, instrumental activities of daily living; K-SDQ, Korean version of the Shoulder Disability Questionnaire; LEMS, Lower Extremity Motor Score; MAL, Motor Activity Log; MAS, Modified Ashworth Scale; MBI, Modified Barthel Index; MeEAP, Measure of Experiential Aspects of Participation; mEFAP, modified Emory Functional Ambulation Profile; MI, Motricity Index; MM, Mobility Milestones; MMAS, Modified Motor Assessment Scale; MMSE, Mini-Mental State Examination; MMT, Manual Muscle Testing; MPS, Motor Power Scale; MRC, Medical Research Council; mRS, modified Rankin Scale; MSQOL-54, Multiple Sclerosis Quality of Life-54; MSS, Motor Status Scale; NHPT, Nine-Hole Peg Test; PASS, Postural Assessment Scale for Stroke; PEF, peak expiratory flow; POMA-B, Performance-Oriented Mobility Assessment Balance subscale; pROM, passive range of motion; QUEST, Quality of Upper Extremity Skills Test; QuickDASH, Quick version of the Disabilities of the Arm, Shoulder, and Hand questionnaire; RAND-36, Rand-36 Item Health Survey; RMA, Rivermead Motor Assessment; RMI, Rivermead Mobility Index; SAS, Stroke Activity Scale; SCIM, Spinal Cord Independence Measure; SF-36, Short-Form 36; SIS, Stroke Impact Scale; SOT, Sensory Organization Balance Test; SPPB, Short Physical Performance Battery; T25FW, Timed 25-Foot Walk; TUG, Timed Up and Go; TWT, Timed Walking Test; UPDRS, Unified Parkinson's Disease Rating Scale; VAS, Visual Analog Scale; WAM, Whole Arm Manipulator; WHS, Walking Handicap Scale; WISCI, Walking Index for Spinal Cord Injury; WMFT, Wolf Motor Function Test
