# Supplementary material for: Effectiveness of Robotic Devices for Medical Rehabilitation: An Umbrella Review
Source: J Clin Med. 2024 Nov 4;13(21):6616. doi: 10.3390/jcm13216616 (PMC11546060; doi:10.3390/jcm13216616)
Supplement: Supplementary file 1 [file jcm-13-06616-s001.zip › Table S9.pdf]

Table S9 Summary of evidence grading and statistics used in meta-analyses of upper-limb devices

Disease	Study	Outcome categories	Outcomes	Subgroups	Details of subgroups	Number of participants	Number of RCTs	Effect model	Effect size metric	Effect size (95% CI)	P	I^2	Grading	Classification of dose matching										
														A	B	C	D	E	F	G	H	I	J	Unknown
Stroke	Lee (2023) [19]	Motor control	FMA-UE	-	-	856	14	Random	SMD	0.23 [0.01 0.45]	0.04	39.36	Weak	5	3	-	-	-	-	-	1	-	5	-
		Motor control	FMA-UE	Period of onset	Chronic	223	6	Random	SMD	0.08 [-0.18 0.34]	0.7	0	Nonsignificant	-	2	-	-	-	-	-	-	-	4	-
		Motor control	FMA-UE	Period of onset	Subacute	149	4	Random	SMD	0.62 [0.08 1.16]	0.07	56.84	Nonsignificant	3	-	-	-	-	-	-	1	-	-	-
		Motor control	FMA-UE	Period of onset	-	372	10	Random	SMD	0.33 [0.04 0.63]	0.08	40.95	Nonsignificant	3	2	-	-	-	-	-	1	-	4	-
		Motor control	FMA-UE	Type of robot device	End-effector	786	10	Random	SMD	0.24 [-0.01 0.50]	0.03	52.37	Weak	4	1	-	-	-	-	-	1	-	4	-
		Motor control	FMA-UE	Type of robot device	Exoskelton	70	4	Random	SMD	0.24 [-0.24 0.71]	0.53	0	Nonsignificant	1	2	-	-	-	-	-	-	-	1	-
		Motor control	FMA-UE	Type of robot device	-	856	14	Random	SMD	0.23 [0.01 0.45]	0.98	39.36	Nonsignificant	5	3	-	-	-	-	-	1	-	5	-
		Motor control	FMA-UE	Duration of the intervention	3 to 4 weeks	178	7	Random	SMD	0.43 [0.08 0.78]	0.24	25.2	Nonsignificant	4	1	-	-	-	-	-	-	-	2	-
		Motor control	FMA-UE	Duration of the intervention	> 4 weeks	640	4	Random	SMD	0.10 [-0.16 0.36]	0.14	39.45	Nonsignificant	1	2	-	-	-	-	-	-	-	1	-
		Motor control	FMA-UE	Duration of the intervention	-	818	11	Random	SMD	0.25 [0.01 0.48]	0.04	44.01	Weak	5	3	-	-	-	-	-	-	-	3	-
		Motor control	FMA-UE	Total number of intervention sessions per week	5 times a week	746	11	Random	SMD	0.22 [-0.02 0.46]	0.08	40.88	Nonsignificant	5	3	-	-	-	-	-	-	-	3	-
		Motor control	FMA-UE	Total number of intervention sessions per week	< 5times a week	82	2	Random	SMD	0.27 [-0.60 1.14]	0.16	49.14	Nonsignificant	-	-	-	-	-	-	-	1	-	1	-
		Motor control	FMA-UE	Total number of intervention sessions per week	-	828	13	Random	SMD	0.25 [0.01 0.48]	0.04	44.01	Weak	5	3	-	-	-	-	-	1	-	4	-
		Motor control	FMA-UE	Duration per each session	30-60 minutes	712	8	Random	SMD	0.30 [-0.03 0.63]	0.01	65.23	Weak	2	3	-	-	-	-	-	1	-	2	-
		Motor control	FMA-UE	Duration per each session	> 60 minutes	116	5	Random	SMD	0.20 [-0.16 0.57]	0.91	0	Nonsignificant	3	-	-	-	-	-	-	-	-	2	-
		Motor control	FMA-UE	Duration per each session	-	828	13	Random	SMD	0.25 [0.01 0.48]	0.04	44.01	Weak	5	3	-	-	-	-	-	1	-	4	-
		Upper-limb capacity	ARAT, FAT, FM-hand, QuickDASH, SIS	Effect for upper limb	Hand function	692	8	Random	SMD	0.18 [0.03 0.33]	0.02	0	Weak	4	1	-	-	-	-	-	1	-	2	-
		Upper-limb capacity	FAT, MI, MRC, FMA-UE, FMA-SEC	Effect for upper limb	Shoulder and elbow	158	4	Random	SMD	0.26 [-0.26 0.77]	0.32	55.83	Nonsignificant	2	1	-	-	-	-	-	1	-	1	-
		Upper-limb capacity	ARAT, SIS, FAT, FM-hand, QuickDASH, MRC, FMA-WH	Effect for upper limb	Wrist and hand	198	4	Random	SMD	-0.01 [-0.29 0.27]	0.93	0	Nonsignificant	1	2	-	-	-	-	-	-	-	1	-
	Yang (2023) [20]	Motor control	FMA-UE	-	-	1275	14	Random	SMD	0.69 [0.34 1.04]	0.0001	86	Suggestive	2	-	1	-	1	-	-	1	-	-	9
		ADL	MBI	-	-	436	6	Random	SMD	0.95 [0.75 1.15]	< 0.00001	45	Weak	-	-	1	-	-	-	-	-	-	-	5
		Muscle tone	MAS	-	-	485	6	Random	SMD	-1.03 [-2.06 0.01]	< 0.00001	96	Weak	2	-	-	-	-	-	-	-	-	-	4
		ADL	FIM	-	-	113	3	Random	SMD	0.24 [-0.45 0.92]	0.5	64	Nonsignificant	1	-	-	-	-	-	-	1	-	-	1
		Upper-limb capacity	WMFT	-	-	133	3	Random	SMD	0.27 [-0.73 1.27]	0.6	88	Nonsignificant	1	-	-	-	-	-	-	1	-	-	1
		Motor control	FMA-UE	Period of onset	Acute	278	5	Random	SMD	1.04 [0.59 1.50]	< 0.00001	66	Weak	1	-	-	-	-	-	-	1	-	-	3
		Motor control	FMA-UE	Period of onset	Subacute	96	2	Random	SMD	-0.16 [-0.56 0.24]	0.44	0	Nonsignificant	1	-	1	-	-	-	-	-	-	-	-
		Motor control	FMA-UE	Period of onset	Chronic	901	7	Random	SMD	0.68 [0.15 1.21]	0.01	90	Weak	-	-	-	-	1	-	-	-	-	-	6
		Motor control	FMA-UE	Period of onset	-	1275	14	Random	SMD	0.69 [0.34 1.04]	0.0001	87.6	Suggestive	2	-	1	-	1	-	-	1	-	-	9
		Motor control	FMA-UE	Intervention duration	4w	182	4	Random	SMD	1.05 [0.30 1.80]	0.006	78	Weak	-	-	-	-	-	-	-	-	-	-	4
		Motor control	FMA-UE	Intervention duration	6w	187	4	Random	SMD	0.14 [-0.32 0.60]	0.55	59	Nonsignificant	2	-	1	-	1	-	-	-	-	-	-
		Motor control	FMA-UE	Intervention duration	12w	827	4	Random	SMD	1.00 [0.23 1.77]	0.01	95	Weak	-	-	-	-	-	-	-	-	-	-	4
		Motor control	FMA-UE	Intervention duration	-	1196	12	Random	SMD	0.72 [0.33 1.11]	0.05	67.1	Nonsignificant	2	-	1	-	1	-	-	-	-	-	8
		Muscle tone	MAS	Period of onset	Acute	143	2	Random	SMD	-0.38 [-0.78 0.02]	0.02	0	Weak	1	-	-	-	-	-	-	-	-	-	1
		Muscle tone	MAS	Period of onset	Chronic	276	3	Random	SMD	-1.89 [-3.33 -0.44]	0.01	94	Weak	-	-	-	-	-	-	-	-	-	-	3
		Muscle tone	MAS	Period of onset	-	419	5	Random	SMD	-1.26 [-2.40 -0.12]	0.03	96	Weak	1	-	-	-	-	-	-	-	-	-	4
		ADL	MBI	Intervention duration	4w	80	2	Fixed	SMD	0.99 [0.52 1.46]	< 0.0001	0	Weak	-	-	-	-	-	-	-	-	-	-	2
		ADL	MBI	Intervention duration	12w	316	3	Fixed	SMD	1.07 [0.83 1.31]	< 0.00001	0	Weak	-	-	-	-	-	-	-	-	-	-	3
		ADL	MBI	Intervention duration	-	396	5	Random	SMD	1.05 [0.84 1.26]	< 0.00001	0	Weak	-	-	-	-	-	-	-	-	-	-	5
	Moggio (2022) [21]	Muscle strength	MI	-	-	99	4	Random	MD	9.47 [3.91 15.03]	0.0008	86	Weak	3	1	-	-	-	-	-	-	-	-	-
		Upper limb capacity	QuickDASH	-	-	62	2	Fixed	MD	-6.71 [-9.17 -4.25]	0.00001	97	Weak	2	-	-	-	-	-	-	-	-	-	-
		Motor control	FMA-UE	-	-	87	3	Fixed	MD	3.00 [1.97 4.04]	0.00001	68	Weak	2	1	-	-	-	-	-	-	-	-	-
	Zhang (2022) [22]	Motor control	FMA-UE	Total training time	< 15 hours	528	19	Random	SMD	0.26 [-0.02 0.55]	0.07	61	Nonsignificant	6	2	1	-	-	-	-	2	-	5	2
		Motor control	FMA-UE	Total training time	> 15 hours	1777	34	Random	SMD	0.15 [0.05 0.25]	0.005	6	Weak	8	6	-	-	-	-	1	1	-	15	3
		Motor control	FMA-UE	Total training time	-	2305	53	Random	SMD	0.20 [0.09 0.32]	0.0007	37	Suggestive	14	8	1	-	-	-	1	3	-	20	5
		ADL	ABILHAND, BI, FIM, MBI, SIS	Total training time, comparison	< 15 hours, non-robotic therapy	470	15	Random	SMD	0.25 [-0.00, 0.51]	0.05	46	Nonsignificant	4	2	-	-	-	-	-	1	-	8	-
		ADL	ABILHAND, BI, FIM, MBI, SIS	Total training time, comparison	> 15 hours, non-robotic therapy	1298	18	Random	SMD	0.32 [0.12 0.53]	0.002	59	Weak	4	2	-	-	-	-	1	1	-	10	-
		ADL	ABILHAND, BI, FIM, MBI, SIS	-	-	1768	30	Random	SMD	0.32 [0.16 0.47]	< 0.0001	53	Suggestive	9	4	1	-	-	-	1	4	-	10	1
		ADL	ABILHAND, BI, FIM, MBI, SIS	Total training time, comparison	-	1768	33	Random	SMD	0.30 [0.14 0.45]	0.0002	53	Suggestive	9	5	1	-	-	-	1	4	-	12	1
		ADL	ABILHAND, BI, FIM, MBI, SIS	Difference level of impairment	Mild to moderate (22 < FMA-UE < 66)	544	16	Random	SMD	0.27 [0.07 0.48]	0.009	29	Weak	6	1	-	-	-	-	1	2	-	6	-
		ADL	ABILHAND, BI, FIM, MBI, SIS	Difference level of impairment	Severe (FMA-UE < 21)	987	10	Random	SMD	0.21 [-0.01 0.42]	0.06	45	Nonsignificant	1	2	1	-	-	-	-	1	-	4	1
		ADL	ABILHAND, BI, FIM, MBI, SIS	Difference level of impairment	-	1531	26	Random	SMD	0.24 [0.10 0.39]	0.001	37	Suggestive	1	2	1	-	-	-	-	1	-	4	1
		Motor control	FMA-UE	Training mode	Patient-passive	72	2	Random	SMD	-0.09 [-1.04 0.86]	0.85	75	Nonsignificant	-	-	-	-	-	-	-	-	-	-	2
		Motor control	FMA-UE	Training mode	Passive-active	353	15	Random	SMD	0.33 [0.06, 0.59]	0.01	30	Weak	5	3	1	-	-	-	-	3	-	2	1
		Motor control	FMA-UE	Training mode	Patient-active	1664	32	Random	SMD	0.17 [0.03 0.31]	0.02	35	Weak	10	6	1	-	-	-	1	3	-	7	4
		Motor control	FMA-UE	Training mode	-	2089	49	Random	SMD	0.19 [0.07 0.32]	0.002	36	Weak	14	8	1	-	-	-	1	4	-	17	4
		ADL	ABILHAND, BI, FIM, MBI, SIS	Training mode	Patient-passive	104	3	Random	SMD	0.70 [-0.27, 1.67]	0.16	82	Nonsignificant	1	-	-	-	-	-	-	-	-	-	2
		ADL	ABILHAND, BI, FIM, MBI, SIS	Training mode	Passive-active	233	9	Random	SMD	0.42 [0.15, 0.68]	0.002	0	Weak	2	1	-	-	-	-	-	-	-	6	-
		ADL	ABILHAND, BI, FIM, MBI, SIS	Training mode	Patient-active	1240	18	Random	SMD	0.22 [0.03, 0.40]	0.02	47	Weak	6	3	1	-	-	-	1	4	-	2	1
		ADL	ABILHAND, BI, FIM, MBI, SIS	Training mode	-	1577	30	Random	SMD	0.31 [0.15, 0.48]	0.0002	51	Suggestive	9	4	1	-	-	-	1	4	-	10	1
	Zhao (2022) [23]	Motor control	FMA-WH	-	-	333	10	-	MD	3.92 [3.04 -4.80]	< 0.001	31	Weak	2	1	-	-	-	-	-	-	-	4	3
	Rozevink (2021) [24]	Motor control	FMA-UE	Period of onset	Subacute	88	3	-	SMD	1.24 [0.20 2.28]	0.02	79	Weak	3	-	-	-	-	-	-	-	-	-	-
		Motor control	FMA-UE	Period of onset	Chronic	196	8	-	SMD	0.08 [-0.28 0.45]	0.66	30	Nonsignificant	1	4	-	-	-	-	-	-	-	3	-
		Motor control	FMA-UE	-	Overall	284	11	-	SMD	0.39 [-0.08 0.87]	0.1	70	Nonsignificant	4	4	-	-	-	-	-	-	-	3	-
		Motor control	FMA-UE	Follow-up	After 4−36 weeks	210	5	-	SMD	0.14 [-0.13 0.41]	0.31	0	Nonsignificant	2	3	-	-	-	-	-	-	-	-	-
	Wu (2021) [25]	Motor control	FMA-UE	-	-	1906	50	-	MD	0.25 [0.11 0.38]	< 0.001	45.9	Suggestive	17	15	-	-	-	-	-	-	-	18	-
		Motor control	FMA-UE	Unilateral or bilateral	Combined unilateral and bilateral	80	3	-	MD	0.22 [-0.71 1.15]	0.645	74.5	Nonsignificant	-	1	-	-	-	-	-	-	-	2	-
		Motor control	FMA-UE	Unilateral or bilateral	Unilateral	1548	33	-	MD	0.32 [0.15 0.50]	< 0.001	55.9	Suggestive	-	13	-	-	-	-	-	-	13	7	-
		Motor control	FMA-UE	Unilateral or bilateral	Bilateral	312	15	-	MD	0.07 [-0.15 0.28]	0.542	0	Nonsignificant	4	2	-	-	-	-	-	-	-	9	-
		Motor control	FMA-UE	Period of onset	> 3 months	809	32	-	MD	0.33 [0.16 0.50]	< 0.001	34.2	Weak	5	11	-	-	-	-	-	-	-	16	-
		Motor control	FMA-UE	Period of onset	< 3 months	457	11	-	MD	0.21 [-0.12 0.54]	0.22	64.8	Nonsignificant	9	-	-	-	-	-	-	-	-	2	-
		Motor control	FMA-UE	Period of onset	> 6 months	826	21	-	MD	0.26 [0.12 0.41]	< 0.001	2.3	Weak	-	-	-	-	-	-	-	-	-	-	-
		Motor control	FMA-UE	Period of onset	< 6 months	518	14	-	MD	0.17 [-0.08 0.42]	0.177	53.2	Nonsignificant	-	-	-	-	-	-	-	-	-	-	-
		Motor control	FMA-UE	Severity	Moderate-severe	1295	30	-	MD	0.27 [0.08 0.46]	0.004	53	Weak	9	11	-	-	-	-	-	-	-	10	-
		Motor control	FMA-UE	Severity	Mild-moderate	316	14	-	MD	0.19 [-0.01 0.40]	0.063	0	Nonsignificant	3	1	-	-	-	-	-	-	-	10	-
		Motor control	FMA-UE	Severity	Mild-severe	-	6	-	-	0.34 [-0.21 0.89]	-	80	-	5	1	-	-	-	-	-	-	-	-	-
		Motor control	FMA-UE	Type of robot device	End-effector	1605	40	-	MD	0.22 [0.09 0.36]	0.001	35.4	Suggestive	13	6	-	-	-	-	-	-	-	21	-
		Motor control	FMA-UE	Type of robot device	Exoskeleton	301	10	-	MD	0.31 [-0.14 0.76]	0.171	68.9	Nonsignificant	3	5	-	-	-	-	-	-	-	2	-
		Motor control	FMA-UE	Trained part	Proximal joint	1097	21	-	MD	0.27 [0.05 0.50]	0.018	61.9	Weak	8	7	-	-	-	-	-	-	-	6	-
		Motor control	FMA-UE	Trained part	Distal joint	417	17	-	MD	0.19 [0.01 0.37]	0.042	0	Weak	5	1	-	-	-	-	-	-	-	11	-
	Chen (2020) [26]	Motor control	FMA-UE	-	-	1682	29	Fixed	WMD	0.763 [0.403 1.123]	< 0.001	31.8	Suggestive	11	6	-	-	-	-	-	2	-	8	2
		Motor control	FMA-UE	Trail design	Add-on design	797	20	-	WMD	0.74 [0.29 1.19]	0.001	49.6	Weak	-	-	-	-	-	-	-	-	-	-	-
		Motor control	FMA-UE	Trail design	Alone design	885	9	-	WMD	0.80 [0.21 1.39]	0.008	0	Weak	-	-	-	-	-	-	-	-	-	-	-
		Motor control	FMA-UE	Type of robot device	End-effector	1359	23	-	WMD	1.43 [0.84 2.03]	-	14.8	-	-	-	-	-	-	-	-	-	-	-	-
		Motor control	FMA-UE	Type of robot device	Exoskeleton	333	6	-	WMD	0.38 [-0.07 0.83]	-	34.8	-	-	-	-	-	-	-	-	-	-	-	-
		Motor control	FMA-UE	Training part	Proximal	1081	15	-	WMD	1.08 [0.61 1.54]	-	0	-	-	-	-	-	-	-	-	-	-	-	-
		Motor control	FMA-UE	Training part	Distal	400	10	-	WMD	0.24 [-0.35 0.84]	-	52.5	-	-	-	-	-	-	-	-	-	-	-	-
		Motor control	FMA-UE	Training part	Both	201	4	-	WMD	0.80 [-1.48 3.07]	-	56.8	-	-	-	-	-	-	-	-	-	-	-	-
		Motor control	FMA-UE	Bilateral/unilateral	Unilateral	1500	24	-	WMD	0.51 [0.10 0.92]	-	27.7	-	-	-	-	-	-	-	-	-	-	-	-
		Motor control	FMA-UE	Bilateral/unilateral	Bilateral	186	5	-	WMD	1.57 [0.84 2.31]	-	0	-	-	-	-	-	-	-	-	-	-	-	-
		Upper-limb capacity	AMAT, ARAT, BBT, CAHAI, NHPT, WMFT	Period of onset	> 6 months	1557	26	Random	SMD	0.109 [-0.066 0.284]	0.02	56.4	Weak	-	-	-	-	-	-	-	-	-	-	-
		Upper-limb capacity	AMAT, ARAT, BBT, CAHAI, NHPT, WMFT	Trail design	Add-on design	652	16	-	SMD	0.16 [-0.12 0.44]	0.309	66.8	Nonsignificant	-	-	-	-	-	-	-	-	-	-	-
		Upper-limb capacity	AMAT, ARAT, BBT, CAHAI, NHPT, WMFT	Trail design	Alone design	905	10	-	SMD	-0.02 [-0.15 0.11]	0.766	0	Nonsignificant	-	-	-	-	-	-	-	-	-	-	-
		Upper-limb capacity	AMAT, ARAT, BBT, CAHAI, NHPT, WMFT	Type of robot device	End-effector	1207	19	-	SMD	0.10 [-0.11 0.31]	-	56.6	-	-	-	-	-	-	-	-	-	-	-	-
		Upper-limb capacity	AMAT, ARAT, BBT, CAHAI, NHPT, WMFT	Type of robot device	Exoskeleton	350	7	-	SMD	0.15 [-0.21 0.52]	-	62.1	-	-	-	-	-	-	-	-	-	-	-	-
		Upper-limb capacity	AMAT, ARAT, BBT, CAHAI, NHPT, WMFT	Training part	Proximal	1012	13	-	SMD	0.05 [-0.13 0.23]	-	25.9	-	-	-	-	-	-	-	-	-	-	-	-
		Upper-limb capacity	AMAT, ARAT, BBT, CAHAI, NHPT, WMFT	Training part	Distal	354	9	-	SMD	0.27 [-0.20 0.74]	-	77	-	-	-	-	-	-	-	-	-	-	-	-

	Upper-limb capacity	AMAT, ARAT, BBT, CAHAI, NHPT, WMFT	Training part	Both	191	4	-	SMD	-0.07 [-0.04 0.30]	-	30.6	-	-	-	-	-	-	-	-	-	-	-	-
	Upper-limb capacity	AMAT, ARAT, BBT, CAHAI, NHPT, WMFT	Bilateral/unilateral	Unilateral	1583	24	-	SMD	0.18 [0.01 0.36]	-	57.2	-	-	-	-	-	-	-	-	-	-	-	-
	Upper-limb capacity	AMAT, ARAT, BBT, CAHAI, NHPT, WMFT	Bilateral/unilateral	Bilateral	74	2	-	SMD	0.36 [-0.06 0.77]	-	0	-	-	-	-	-	-	-	-	-	-	-	-
	ADL	BI, FIM, MAL, mRS	-	-	1468	26	Fixed	SMD	0.05 [-0.06 0.15]	0.153	19.8	Nonsignificant	-	-	-	-	-	-	-	-	-	-	-
	ADL	BI, FIM, MAL, mRS	Trail design	Add-on design	780	20	-	SMD	0.18 [0.03 0.32]	0.016	0	Weak	-	-	-	-	-	-	-	-	-	-	-
	ADL	BI, FIM, MAL, mRS	Trail design	Alone design	688	6	-	SMD	-0.09 [-0.24 0.06]	0.229	41.9	Nonsignificant	-	-	-	-	-	-	-	-	-	-	-
	ADL	BI, FIM, MAL, mRS	Type of robot device	End-effector	1110	18	-	SMD	-0.01 [-0.13 0.11]	-	9.4	-	-	-	-	-	-	-	-	-	-	-	-
	ADL	BI, FIM, MAL, mRS	Type of robot device	Exoskeleton	358	8	-	SMD	0.24 [0.03 0.46]	-	13.3	-	-	-	-	-	-	-	-	-	-	-	-
	ADL	BI, FIM, MAL, mRS	Training part	Proximal	973	13	-	SMD	-0.02 [-0.15 0.11]	-	29.8	-	-	-	-	-	-	-	-	-	-	-	-
	ADL	BI, FIM, MAL, mRS	Training part	Distal	393	10	-	SMD	0.10 [-0.10 0.31]	-	0	-	-	-	-	-	-	-	-	-	-	-	-
	ADL	BI, FIM, MAL, mRS	Training part	Both	102	3	-	SMD	0.49 [0.10 0.89]	-	0	-	-	-	-	-	-	-	-	-	-	-	-
	ADL	BI, FIM, MAL, mRS	Bilateral/unilateral	Unilateral	1282	21	-	SMD	0.03 [-0.08 0.14]	-	28.7	-	-	-	-	-	-	-	-	-	-	-	-
	ADL	BI, FIM, MAL, mRS	Bilateral/unilateral	Bilateral	186	5	-	SMD	0.17 [-0.13 0.47]	-	0	-	-	-	-	-	-	-	-	-	-	-	-
	Social participation	SF-36, SIS	-	-	849	8	Fixed	SMD	-0.06 [-0.20 0.08]	0.378	35.6	Nonsignificant	-	-	-	-	-	-	-	-	-	-	-
	Social participation	SF-36, SIS	Trail design	Add-on design	191	4	-	SMD	0.12 [-0.17 0.41]	0.417	0	Nonsignificant	-	-	-	-	-	-	-	-	-	-	-
	Social participation	SF-36, SIS	Trail design	Alone design	658	4	-	SMD	-0.11 [-0.27 0.04]	0.153	53.3	Nonsignificant	-	-	-	-	-	-	-	-	-	-	-
	Social participation	SF-36, SIS	Type of robot device	End-effector	732	6	-	SMD	-0.06 [-0.21 0.08]	-	53.4	-	-	-	-	-	-	-	-	-	-	-	-
	Social participation	SF-36, SIS	Type of robot device	Exoskeleton	117	2	-	SMD	-0.04 [-0.40 0.32]	-	0	-	-	-	-	-	-	-	-	-	-	-	-
	Social participation	SF-36, SIS	Training part	Proximal	579	3	-	SMD	-0.09 [-0.20 0.08]	-	78	-	-	-	-	-	-	-	-	-	-	-	-
	Social participation	SF-36, SIS	Training part	Distal	149	3	-	SMD	0.02 [-0.31 0.34]	-	0	-	-	-	-	-	-	-	-	-	-	-	-
	Social participation	SF-36, SIS	Training part	Both	121	2	-	SMD	-0.03 [-0.39 0.32]	-	0	-	-	-	-	-	-	-	-	-	-	-	-
	Social participation	SF-36, SIS	Bilateral/unilateral	Unilateral	807	7	-	SMD	-0.09 [-0.27 0.05]	-	19.8	-	-	-	-	-	-	-	-	-	-	-	-
	Social participation	SF-36, SIS	Bilateral/unilateral	Bilateral	42	1	-	SMD	0.54 [-0.11 1.19]	-	-	-	-	-	-	-	-	-	-	-	-	-	-
Chien (2020) [27]	Motor control	FMA-UE (FMA-Motor, FMA-SEC, FMA-WH)	-	-	274	5	-	SMD	0.18 [-0.16 0.51]	0.31	48	Nonsignificant	5	-	-	-	-	-	-	-	-	-	-
	ADL	ACTIVLIM questionnaire, BI, FIM (cognition, motor, self-care)	-	-	183	4	-	SMD	0.40 [-0.16 0.95]	0.16	71	Nonsignificant	3	-	-	-	-	-	-	-	1	-	-
	Upper-limb capacity	ARAT, QuickDASH, WMFT	-	-	219	4	-	SMD	0.01 [-0.28 0.30]	0.96	12	Nonsignificant	4	-	-	-	-	-	-	-	-	-	-
	Range of motion	MAS	-	-	135	3	-	SMD	-0.04 [-0.38 0.30]	0.81	0	Nonsignificant	3	-	-	-	-	-	-	-	-	-	-
	Comprehensive measures	SIS	-	-	149	3	-	SMD	0.03 [-0.30 0.36]	0.86	0	Nonsignificant	1	-	-	-	-	-	1	-	-	1	-
Ferreira (2018) [28]	Motor control	CMSA, FMA-UE, MSS	Intervention term, comparison	Short term, minimum	137	5	Fixed	SMD	0.2 [-0.1 0.5]	0.218	0	Nonsignificant	-	1	-	-	-	-	-	1	3	-	-
	Motor control	CMSA, FMA-UE, MSS	Intervention term, comparison	Medium term, minimum	19	1	-	SMD	0.4 [-0.5 1.3]	0.367	-	Nonsignificant	-	1	-	-	-	-	-	-	-	-	-
	Motor control	CMSA, FMA-UE, MSS	Intervention term, comparison	Long term, minimum	12	1	-	SMD	-0.0 [-1.1 1.1]	0.98	-	Nonsignificant	-	-	-	-	-	-	-	-	1	-	-
	Motor control	CMSA, FMA-UE, MSS	Intervention term, comparison	Short term, other interventions	595	22	Fixed	SMD	0.3 [0.1 0.4]	0.001	45.68	Weak	2	6	-	1	-	-	1	1	-	11	-
	Motor control	CMSA, FMA-UE, MSS	Intervention term, comparison	Medium term, other interventions	205	6	Fixed	SMD	0.4 [0.1 0.7]	0.01	17.64	Weak	-	1	-	-	-	-	1	-	-	4	-
	Motor control	CMSA, FMA-UE, MSS	Intervention, intervention term, comparison	RT add, short term, other interventions	184	9	Fixed	SMD	0.2 [-0.1 0.5]	0.238	0	Nonsignificant	2	4	-	-	-	-	-	-	-	3	-
	Motor control	CMSA, FMA-UE, MSS	Intervention, intervention term, comparison	RT add, medium term, other interventions	52	2	Random	SMD	0.5 [-0.6 1.6]	0.39	0	Nonsignificant	-	1	-	-	-	-	-	-	1	-	-
	Muscle tone	MAS	Intervention term, comparison	Short term, minimum	30	1	-	SMD	0.4 [-0.3 1.1]	0.253	-	Nonsignificant	-	-	-	-	-	-	-	-	1	-	-
	Muscle tone	MAS	Intervention term, comparison	Short term, other interventions	281	8	Random	SMD	-0.5 [-1.4 0.4]	0.283	6.96	Nonsignificant	1	1	-	-	-	-	1	-	-	5	-
	Muscle tone	MAS	Intervention term, comparison	Medium term, other interventions	88	3	Random	SMD	0.1 [-0.6 0.9]	0.714	9.8	Nonsignificant	-	-	-	-	-	-	1	-	-	2	-
	Muscle tone	MAS	Intervention, intervention term, comparison	RT add, short term, other interventions	78	4	Random	SMD	-0.4 [-1.1 0.3]	0.252	0	Nonsignificant	1	-	-	-	-	-	-	-	-	3	-
	Muscle tone	MAS	Intervention, intervention term, comparison	RT add, medium term, other interventions	30	1	-	SMD	-0.7 [-1.4 0.1]	0.076	-	Nonsignificant	1	-	-	-	-	-	-	-	-	-	-
	Muscle strength	Grip strength, maximum resistive force with WAM control program, MI, MMT, motor power range, MPS, MRC	Intervention term, comparison	Short term, other interventions	103	10	Fixed	SMD	0.5 [0.2 0.8]	0.002	32.08	Weak	-	1	-	-	-	-	1	-	-	8	-
	Muscle strength	Grip strength, maximum resistive force with WAM control program, MI, MMT, motor power range, MPS, MRC	Intervention term, comparison	Medium term, other interventions	110	5	Random	SMD	0.3 [-0.5 1.1]	0.474	0	Nonsignificant	-	-	-	-	-	-	-	-	-	5	-
	Muscle strength	Grip strength, maximum resistive force with WAM control program, MI, MMT, motor power range, MPS, MRC	Intervention, intervention term, comparison	RT add, short term, other interventions	28	2	Fixed	SMD	-0.1 [-0.8 0.7]	0.863	0	Nonsignificant	-	-	-	-	-	-	-	-	-	2	-
	Motor control	CMSA, FMA-UE, MSS	Methodological quality	Over 5 out of 10	-	-	Random	SMD	0.3 [0.1 0.5]	0.001	43.44	Weak	-	-	-	-	-	-	-	-	-	-	-
	Muscle tone	MAS	Methodological quality	Over 5 out of 10	-	-	Random	SMD	-0.6 [-1.8 0.5]	0.255	21.59	Nonsignificant	-	-	-	-	-	-	-	-	-	-	-
	Muscle strength	Grip strength, maximum resistive force with WAM control program, MI, MMT, motor power range, MPS, MRC	Methodological quality	Over 5 out of 10	53	1	Random	SMD	0.4 [-0.3 1.2]	0.247	0	Nonsignificant	1	-	-	-	-	-	-	-	-	-	-
	Range of motion	pROM with the assistance of WAM or therapist for the elbows, total pROM	Methodological quality	Over 5 out of 10	-	-	-	SMD	0.2 [-0.4 0.7]	0.491	-	Nonsignificant	-	-	-	-	-	-	-	-	-	-	-
	Pain	CMSA Pain Inventory Scale range 1–7, Pain Scale of FM, VAS	Methodological quality	Over 5 out of 10	-	-	Fixed	SMD	-0.1 [-0.5 0.3]	0.535	14.48	Nonsignificant	-	-	-	-	-	-	-	-	-	-	-
	Motor control	CMSA, FMA-UE, MSS	Intervention	Proximal	-	-	Random	SMD	0.3 [-0.0 0.7]	0.061	0	Nonsignificant	-	-	-	-	-	-	-	-	-	-	-
	Motor control	CMSA, FMA-UE, MSS	Intervention	Distal	-	-	Random	SMD	0.2 [-0.4 0.8]	0.573	0	Nonsignificant	-	-	-	-	-	-	-	-	-	-	-
	Muscle tone	MAS	Intervention	Proximal	-	-	Random	SMD	0.5 [-0.1 1.0]	0.101	0	Nonsignificant	-	-	-	-	-	-	-	-	-	-	-
	Muscle tone	MAS	Intervention	Distal	-	-	Random	SMD	0.0 [-0.7 0.8]	0.959	22.09	Nonsignificant	-	-	-	-	-	-	-	-	-	-	-
	Muscle strength	Grip strength, maximum resistive force with WAM control program, MI, MMT, motor power range, MPS, MRC	Intervention	Proximal	-	-	Random	SMD	-0.6 [-2.0 0.8]	0.414	89.07	Nonsignificant	-	-	-	-	-	-	-	-	-	-	-
	Muscle strength	Grip strength, maximum resistive force with WAM control program, MI, MMT, motor power range, MPS, MRC	Intervention	Distal	-	-	Random	SMD	0.5 [-0.8 1.9]	0.452	8.27	Nonsignificant	-	-	-	-	-	-	-	-	-	-	-
	Range of motion	pROM with the assistance of WAM or therapist for the elbows, total pROM	Intervention	Proximal	-	-	Random	SMD	-1.4 [-3.7 0.8]	0.214	15.95	Nonsignificant	-	-	-	-	-	-	-	-	-	-	-
	Range of motion	pROM with the assistance of WAM or therapist for the elbows, total pROM	Intervention	Distal	-	-	Random	SMD	-10.8 [-17.6 -4.0]	0.002	30.87	Weak	-	-	-	-	-	-	-	-	-	-	-
	Motor control	CMSA, FMA-UE, MSS	Dose	Same amount	-	-	Random	SMD	0.3 [0.2 0.5]	0.000	47.81	Weak	-	-	-	-	-	-	-	-	-	-	-
	Motor control	CMSA, FMA-UE, MSS	Dose	Different amount	-	-	Fixed	SMD	-0.1 [-0.5 0.3]	0.578	38.77	Nonsignificant	-	-	-	-	-	-	-	-	-	-	-
	Muscle tone	MAS	Dose	Same amount	-	-	Fixed	SMD	-0.7 [-2.0 0.6]	0.32	0	Nonsignificant	-	-	-	-	-	-	-	-	-	-	-
	Muscle tone	MAS	Dose	Different amount	-	-	Fixed	SMD	-0.1 [-0.5 0.4]	0.787	16.56	Nonsignificant	-	-	-	-	-	-	-	-	-	-	-
	Muscle strength	Grip strength, maximum resistive force with WAM control program, MI, MMT, motor power range, MPS, MRC	Dose	Same amount	-	-	Fixed	SMD	0.9 [-0.4 2.1]	0.185	30.65	Nonsignificant	-	-	-	-	-	-	-	-	-	-	-
	Muscle strength	Grip strength, maximum resistive force with WAM control program, MI, MMT, motor power range, MPS, MRC	Dose	Different amount	-	-	Fixed	SMD	0.0 [-0.4 0.4]	0.85	0	Nonsignificant	-	-	-	-	-	-	-	-	-	-	-
	Range of motion	pROM with the assistance of WAM or therapist for the elbows, total pROM	Dose	Same amount	-	-	Fixed	SMD	-0.1 [-0.5 0.3]	0.705	0	Nonsignificant	-	-	-	-	-	-	-	-	-	-	-
	Pain	CMSA Pain Inventory Scale range 1–7, Pain Scale of FM, VAS	Dose	Same amount	-	-	Fixed	SMD	-0.1 [-0.5 0.2]	0.413	0	Nonsignificant	-	-	-	-	-	-	-	-	-	-	-
	Motor control	CMSA, FMA-UE, MSS	Number of sessions	≤ 20 sessions	-	-	Fixed	SMD	0.3 [-0.0 0.6]	0.073	0	Nonsignificant	-	-	-	-	-	-	-	-	-	-	-
	Motor control	CMSA, FMA-UE, MSS	Number of sessions	> 20 sessions	-	-	Random	SMD	0.4 [0.1 0.6]	0.001	16.22	Weak	-	-	-	-	-	-	-	-	-	-	-
	Muscle tone	MAS	Number of sessions	≤ 20 sessions	-	-	Fixed	SMD	0.0 [-0.6 0.6]	0.926	0	Nonsignificant	-	-	-	-	-	-	-	-	-	-	-
	Muscle tone	MAS	Number of sessions	> 20 sessions	-	-	Random	SMD	-0.9 [-2.6 0.8]	0.283	24.85	Nonsignificant	-	-	-	-	-	-	-	-	-	-	-
	Muscle strength	Grip strength, maximum resistive force with WAM control program, MI, MMT, motor power range, MPS, MRC	Number of sessions	≤ 20 sessions	-	-	Fixed	SMD	0.1 [-0.7 0.9]	0.799	16.1	Nonsignificant	-	-	-	-	-	-	-	-	-	-	-
	Muscle strength	Grip strength, maximum resistive force with WAM control program, MI, MMT, motor power range, MPS, MRC	Number of sessions	> 20 sessions	-	-	Random	SMD	1.7 [-0.0 3.4]	0.054	33.54	Nonsignificant	-	-	-	-	-	-	-	-	-	-	-
	Range of motion	pROM with the assistance of WAM or therapist for the elbows, total pROM	Number of sessions	≤ 20 sessions	-	-	Random	SMD	-0.3 [-1.2 0.6]	0.504	0	Nonsignificant	-	-	-	-	-	-	-	-	-	-	-
	Range of motion	pROM with the assistance of WAM or therapist for the elbows, total pROM	Number of sessions	> 20 sessions	-	-	Random	SMD	-0.0 [-0.5 0.4]	0.923	37.52	Nonsignificant	-	-	-	-	-	-	-	-	-	-	-
	Pain	CMSA Pain Inventory Scale range 1–7, Pain Scale of FM, VAS	Number of sessions	≤ 20 sessions	-	-	Random	SMD	-0.1 [-0.7 0.6]	0.842	0	Nonsignificant	-	-	-	-	-	-	-	-	-	-	-
	Pain	CMSA Pain Inventory Scale range 1–7, Pain Scale of FM, VAS	Number of sessions	> 20 sessions	-	-	Random	SMD	-0.2 [-0.6 0.2]	0.367	0	Nonsignificant	-	-	-	-	-	-	-	-	-	-	-
	Motor control	CMSA, FMA-UE, MSS	Total volume	≤ 20 hours	-	-	Random	SMD	0.2 [-0.3 0.7]	0.401	0	Nonsignificant	-	-	-	-	-	-	-	-	-	-	-
	Motor control	CMSA, FMA-UE, MSS	Total volume	> 20 hours	-	-	Random	SMD	0.3 [0.1 0.5]	0.009	39.48	Weak	-	-	-	-	-	-	-	-	-	-	-
	Muscle tone	MAS	Total volume	≤ 20 hours	-	-	Random	SMD	-0.1 [-0.5 0.3]	0.57	0	Nonsignificant	-	-	-	-	-	-	-	-	-	-	-
	Muscle tone	MAS	Total volume	> 20 hours	-	-	Random	SMD	-1.2 [-3.6 1.1]	0.316	17.85	Nonsignificant	-	-	-	-	-	-	-	-	-	-	-
	Muscle strength	Grip strength, maximum resistive force with WAM control program, MI, MMT, motor power range, MPS, MRC	Total volume	≤ 20 hours	-	-	Random	SMD	0.7 [-0.1 1.4]	0.099	0	Nonsignificant	-	-	-	-	-	-	-	-	-	-	-
	Muscle strength	Grip strength, maximum resistive force with WAM control program, MI, MMT, motor power range, MPS, MRC	Total volume	> 20 hours	-	-	Random	SMD	-0.3 [-1.2 0.7]	0.594	28.13	Nonsignificant	-	-	-	-	-	-	-	-	-	-	-
	Range of motion	pROM with the assistance of WAM or therapist for the elbows, total pROM	Total volume	≤ 20 hours	-	-	Fixed	SMD	-0.3 [-1.2 0.6]	0.504	0	Nonsignificant	-	-	-	-	-	-	-	-	-	-	-
	Range of motion	pROM with the assistance of WAM or therapist for the elbows, total pROM	Total volume	> 20 hours	-	-	Fixed	SMD	-0.0 [-0.5 0.4]	0.923	37.52	Nonsignificant	-	-	-	-	-	-	-	-	-	-	-
	Pain	CMSA Pain Inventory Scale range 1–7, Pain Scale of FM, VAS	Total volume	≤ 20 hours	-	-	Fixed	SMD	-0.1 [-0.7 0.6]	0.842	0	Nonsignificant	-	-	-	-	-	-	-	-	-	-	-
	Pain	CMSA Pain Inventory Scale range 1–7, Pain Scale of FM, VAS	Total volume	> 20 hours	-	-	Fixed	SMD	-0.2 [-0.6 0.2]	0.395	0	Nonsignificant	-	-	-	-	-	-	-	-	-	-	-
	Motor control	CMSA, FMA-UE, MSS	Period of onset	Acute	-	-	Random	SMD	0.4 [-0.4 1.1]	0.356	4.01	Nonsignificant	-	-	-	-	-	-	-	-	-	-	-
	Motor control	CMSA, FMA-UE, MSS	Period of onset	Chronic	-	-	Fixed	SMD	0.2 [0.0 0.4]	0.026	26.66	Weak	-	-	-	-	-	-	-	-	-	-	-
	Muscle tone	MAS	Period of onset	Acute	-	-	Fixed	SMD	-0.1 [-0.4 0.3]	0.757	0	Nonsignificant	-	-	-	-	-	-	-	-	-	-	-
	Muscle tone	MAS	Period of onset	Chronic	-	-	Random	SMD	-0.8 [-2.5 0.9]	0.36	0	Nonsignificant	-	-	-	-	-	-	-	-	-	-	-
	Muscle strength	Grip strength, maximum resistive force with WAM control program, MI, MMT, motor power range, MPS, MRC	Period of onset	Acute	-	-	Random	SMD	0.0 [-0.9 1.0]	0.967	27.02	Nonsignificant	-	-	-	-	-	-	-	-	-	-	-
	Muscle strength	Grip strength, maximum resistive force with WAM control program, MI, MMT, motor power range, MPS, MRC	Period of onset	Chronic	-	-	Random	SMD	1.2 [-0.0 2.3]	0.051	16.51	Nonsignificant	-	-	-	-	-	-	-	-	-	-	-
	Range of motion	pROM with the assistance of WAM or therapist for the elbows, total pROM	Period of onset	Acute	53	1	-	SMD	0.2 [-0.4 0.7]	0.491	-	Nonsignificant	1	-	-	-	-	-	-	-	-	-	-
	Range of motion	pROM with the assistance of WAM or therapist for the elbows, total pROM	Period of onset	Chronic	-	-	Fixed	SMD	-0.4 [-0.9 0.2]	0.212	0	Nonsignificant	-	-	-	-	-	-	-	-	-	-	-
	Pain	CMSA Pain Inventory Scale range 1–7, Pain Scale of FM, VAS	Period of onset	Acute	19	1	-	SMD	0.3 [-0.6 1.2]	0.565	-	Nonsignificant	-	1	-	-	-	-	-	-	-	-	-
	Pain	CMSA Pain Inventory Scale range 1–7, Pain Scale of FM, VAS	Period of onset	Chronic	-	-	Fixed	SMD	-0.2 [-0.6 0.2]	0.264	0	Nonsignificant	-	-	-	-	-	-	-	-	-	-	-
Mehrholz (2018) [29]	ADL	ABILHAND, BI, FIM, Frenchay Arm Test, MBI, SIS 2.0, SIS 3.0 (motor function, social participation)	Comparison	Other interventions	957	24	Random	SMD	0.31 [0.09 0.52]	0.0049	59	Weak	9	3	-	-	-	-	1	2	2	6	1
	ADL	ABILHAND, BI, FIM, Frenchay Arm Test, MBI, SIS 2.0, SIS 3.0 (motor function, social participation)	Comparison, period of onset	Other interventions, acute (within 3 months)	532	13	Random	SMD	0.40 [0.10 0.70]	0.0085	63	Weak	7	-	-	-	-	-	1	-	2	3	-
	ADL	ABILHAND, BI, FIM, Frenchay Arm Test, MBI, SIS 2.0, SIS 3.0 (motor function, social participation)	Comparison, period of onset	Other interventions, chronic (more than 3 months)	425	11	Random	SMD	0.19 [-0.13 0.50]	0.24	54	Nonsignificant	2	3	-	-	-	-	-	2	-	3	1
	Motor control	CMSA, FMA-UE, WMFT	-	-	1452	41	Random	SMD	0.32 [0.18 0.46]	< 0.00001	36	Suggestive	12	9	1	1	-	-	1	3	2	9	3
	Muscle strength	Grip force, MI, MRC	-	-	826	23	Random	SMD	0.46 [0.16 0.77]	0.0032	76	Weak	9	3	1	-	-	-	1	1	2	4	2

	Other measures	Dropouts during the intervention period	-	-	1619	45	Random	SMD	0.00 [-0.02 0.02]	0.93	0	Nonsignificant	13	12	1	1	-	-	1	3	2	9	3
	ADL	ABILHAND, BI, FIM, Frenchay Arm Test, MBI, SIS 2.0, SIS 3.0 (motor function, social participation)	Sensitivity analysis: by trial methodology	All studies with description of randomization procedure	661	15	Random	SMD	0.32 [0.15 0.49]	0.00018	9	Weak	8	3	-	-	-	-	1	-	-	3	-
	ADL	ABILHAND, BI, FIM, Frenchay Arm Test, MBI, SIS 2.0, SIS 3.0 (motor function, social participation)	Sensitivity analysis: by trial methodology	All studies with adequate concealed allocation	392	10	Random	SMD	0.28 [0.03 0.52]	0.028	30	Weak	5	1	-	-	-	-	1	-	-	3	-
	ADL	ABILHAND, BI, FIM, Frenchay Arm Test, MBI, SIS 2.0, SIS 3.0 (motor function, social participation)	Sensitivity analysis: by trial methodology	All studies with blinded assessors	808	20	Random	SMD	0.29 [0.10 0.49]	0.0035	41	Weak	8	2	-	-	-	-	1	2	1	6	-
	Motor control	CMSA, FMA-UE, WMFT	Sensitivity analysis: by trial methodology	All studies with description of randomization procedure	1048	29	Random	SMD	0.32 [0.16 0.47]	0.000058	28	Suggestive	10	9	1	-	-	-	1	-	-	6	2
	Motor control	CMSA, FMA-UE, WMFT	Sensitivity analysis: by trial methodology	All studies with adequate concealed allocation	532	12	Random	SMD	0.43 [0.21 0.64]	0.00013	21	Weak	5	2	-	-	-	-	1	-	-	4	-
	Motor control	CMSA, FMA-UE, WMFT	Sensitivity analysis: by trial methodology	All studies with blinded assessors	1220	32	Random	SMD	0.33 [0.18 0.49]	0.000022	37	Suggestive	11	7	1	1	-	-	1	2	1	8	-
	ADL	ABILHAND, BI, FIM, Frenchay Arm Test, MBI, SIS 2.0, SIS 3.0 (motor function, social participation)	Treatment approach	Distal training (finger, hand and radio-ulnar joints)	255	8	Random	SMD	0.37 [0.08 0.67]	0.013	23	Weak	-	-	-	-	-	-	-	-	-	-	-
	ADL	ABILHAND, BI, FIM, Frenchay Arm Test, MBI, SIS 2.0, SIS 3.0 (motor function, social participation)	Treatment approach	Proximal training (shoulder and elbow joints)	702	16	Random	SMD	0.28 [-0.01 0.56]	0.056	68	Nonsignificant	-	-	-	-	-	-	-	-	-	-	-
	Motor control	CMSA, FMA-UE, WMFT	Treatment approach	Distal training (finger, hand and radio-ulnar joints)	547	17	Random	SMD	0.34 [0.09 0.59]	0.0085	48	Weak	-	-	-	-	-	-	-	-	-	-	-
	Motor control	CMSA, FMA-UE, WMFT	Treatment approach	Proximal training (shoulder and elbow joints)	905	24	Random	SMD	0.31 [0.15 0.48]	0.0002	27	Weak	-	-	-	-	-	-	-	-	-	-	-
Bertani (2017) [30]	Motor control	Fugl-Meyer scale	-	-	576	15	Fixed	SMD	0.21 [0.04 0.38]	0.01	54.9	Weak	4	4	-	-	-	-	1	-	1	5	-
	Motor control	Fugl-Meyer scale	-	-	576	15	Random	SMD	0.21 [-0.05 0.47]	-	54.9	-	4	4	-	-	-	-	1	-	1	5	-
	Muscle tone	Modified Ashworth Scale	-	-	385	9	Fixed	SMD	-0.15 [-0.35 0.05]	0.15	0	Nonsignificant	2	1	-	-	-	-	1	-	1	4	-
	Muscle tone	Modified Ashworth Scale	-	-	385	9	Random	SMD	-0.15 [-0.35 0.06]	0.15	0	Nonsignificant	2	1	-	-	-	-	1	-	1	4	-
	ADL	FIM, MAL	-	-	242	8	Fixed	SMD	0.60 [0.32 0.88]	-	81.9	Nonsignificant	2	2	-	-	-	-	-	-	1	3	-
	ADL	FIM, MAL	-	-	242	8	Random	SMD	0.51 [-0.15 1.17]	0.13	81.9	Nonsignificant	2	2	-	-	-	-	-	-	1	3	-
	ADL	FIM	Period of onset	Subacute	237	6	Fixed	SMD	0.13 [-0.15 0.40]	-	81.3	Nonsignificant	2	-	-	-	-	-	1	-	1	2	-
	ADL	FIM	Period of onset	Subacute	237	6	Random	SMD	0.12 [-0.52 0.77]	0.7	81.3	Nonsignificant	2	-	-	-	-	-	1	-	1	2	-
	ADL	FIM	Period of onset	Chronic	339	9	Fixed	SMD	0.26 [0.05 0.47]	0.01	0	Weak	2	4	-	-	-	-	-	-	-	3	-
	ADL	FIM	Period of onset	Chronic	339	9	Random	SMD	0.26 [0.05 0.47]	0.01	0	Weak	2	4	-	-	-	-	-	-	-	3	-
	Motor control	Fugl-Meyer scale	Type of robot device	End-effector	453	12	Fixed	SMD	0.15 [-0.04 0.34]	0.11	62	Nonsignificant	3	2	-	-	-	-	1	-	1	5	-
	Motor control	Fugl-Meyer scale	Type of robot device	End-effector	453	12	Random	SMD	0.17 [-0.15 0.49]	-	62	Nonsignificant	3	2	-	-	-	-	1	-	1	5	-
	Motor control	Fugl-Meyer scale	Type of robot device	Exoskeleton	123	3	Fixed	SMD	0.43 [0.07 0.79]	0.01	0	Weak	1	2	-	-	-	-	-	-	-	-	-
	Motor control	Fugl-Meyer scale	Type of robot device	Exoskeleton	123	3	Random	SMD	0.43 [0.07 0.79]	-	0	Nonsignificant	1	2	-	-	-	-	-	-	-	-	-
Veerbeek (2017) [32]	Motor control	FMA-arm score	-	Overall	884	34	Fixed	MD	2.23 [0.87 3.59]	0.001	30	Weak	6	9	-	1	-	-	1	1	1	14	1
	Motor control	FMA-SEC score	Joints targeted	Proximal	369	17	Fixed	MD	2.62 [1.48 3.76]	< 0.00001	34	Weak	2	2	1	1	-	-	1	-	3	7	-
	Motor control	FMA-WH score	Joints targeted	Distal	443	21	Random	MD	1.22 [-0.61 3.05]	0.19	75	Nonsignificant	2	3	1	1	-	-	1	-	3	10	-
	Muscle strength	MI (arm subscale), MPS, MRC	-	Overall	494	21	Random	SMD	0.19 [-0.12 0.50]	0.22	56	Nonsignificant	4	1	-	-	-	-	1	1	2	12	-
	Muscle tone	AS, MAS	-	Overall	429	18	Fixed	SMD	0.24 [0.04 0.44]	0.02	25	Weak	5	-	-	-	-	-	1	-	2	10	-
	Upper-limb capacity	MAT, ARAT, BBT, WMFT	-	Overall	682	24	Fixed	SMD	0.04 [-0.12 0.19]	0.64	2	Nonsignificant	6	6	1	2	-	-	-	1	-	8	-
	ADL	BI, FIM, mRS	-	Overall	427	17	Random	SMD	0.27 [-0.05 0.59]	0.09	56	Nonsignificant	4	-	-	1	-	-	-	2	3	7	-
	Motor control	FMA-arm score	Type of robot device, joints targeted	Shoulder/elbow robotics, overall	528	17	Fixed	MD	2.45 [0.63 4.27]	0.008	14	Weak	3	5	-	1	-	-	-	1	1	6	-
	Motor control	FMA-SEC score	Type of robot device, joints targeted	Shoulder/elbow robotics, proximal	228	10	Fixed	MD	2.15 [0.73 3.57]	0.003	31	Weak	2	1	-	-	-	-	-	-	3	4	-
	Motor control	FMA-WH score	Type of robot device, joints targeted	Shoulder/elbow robotics, distal	290	13	Fixed	MD	0.66 [-0.39 1.72]	0.22	33	Nonsignificant	2	1	-	-	-	-	-	-	3	7	-
	Muscle strength	MI (arm subscale), MPS, MRC	Type of robot device	Shoulder/elbow robotics	254	10	Fixed	SMD	0.36 [0.10 0.63]	0.006	44	Weak	1	1	-	-	-	-	-	1	1	6	-
	Muscle tone	AS, MAS	Type of robot device	Shoulder/elbow robotics	206	10	Random	SMD	0.43 [-0.02 0.87]	0.06	50	Nonsignificant	2	1	-	-	-	-	-	-	1	6	-
	Upper-limb capacity	AMAT, ARAT, BBT, WMFT	Type of robot device	Shoulder/elbow robotics	413	15	Fixed	SMD	0.07 [-0.13 0.27]	0.49	17	Nonsignificant	2	5	-	1	-	-	-	1	-	6	-
	ADL	FIM	Type of robot device	Shoulder/elbow robotics	330	14	Random	SMD	0.24 [-.017 0.65]	0.25	64	Nonsignificant	2	-	-	-	-	-	-	2	3	7	-
	Motor control	FMA-arm score	Type of robot device	Whole-arm robotics	62	2	Random	MD	2.17 [-11.90 16.23]	0.76	81	Nonsignificant	1	1	-	-	-	-	-	-	-	-	-
	Upper-limb capacity	AMAT, ARAT, BBT, WMFT	Type of robot device	Whole-arm robotics	62	2	Random	SMD	0.03 [-1.03 1.08]	0.96	63	Nonsignificant	1	1	-	-	-	-	-	-	-	-	-
	Motor control	FMA-arm score	Type of robot device	Shoulder/elbow/wrist robotics	101	4	Fixed	MD	-0.36 [-3.48 2.76]	0.82	82	Nonsignificant	1	-	-	-	-	-	-	-	-	2	1
	Muscle strength	MI (arm subscale), MPS, MRC	Type of robot device	Shoulder/elbow/wrist robotics	88	3	Fixed	SMD	-0.14 [-0.57 0.29]	0.53	22	Nonsignificant	1	-	-	-	-	-	-	-	-	2	-
	Muscle tone	AS, MAS	Type of robot device	Shoulder/elbow/wrist robotics	88	3	Fixed	SMD	0.42 [-0.01 0.84]	0.06	0	Nonsignificant	1	-	-	-	-	-	-	-	-	2	-
	Motor control	FMA-arm score	Type of robot device	Elbow/wrist robotics	131	7	Fixed	MD	6.55 [2.60 10.51]	0.001	46	Weak	1	-	-	-	-	-	1	-	-	5	-
	Motor control	FMA-SEC score	Type of robot device, joints targeted	Elbow/wrist robotics, proximal	65	3	Fixed	MD	3.51 [0.96 6.07]	0.007	44	Weak	-	-	-	-	-	-	1	-	-	2	-
	Motor control	FMA-WH score	Type of robot device, joints targeted	Elbow/wrist robotics, distal	65	3	Random	MD	4.88 [-1.22 10.99]	0.12	53	Nonsignificant	-	-	-	-	-	-	1	-	-	2	-
	Muscle strength	MI (arm subscale), MPS, MRC	Type of robot device	Elbow/wrist robotics	82	5	Random	SMD	0.51 [-0.31 1.34]	0.22	59	Nonsignificant	-	-	-	-	-	-	1	-	-	4	-
	Motor control	FMA-SEC score	Type of robot device, joints targeted	Wrist/hand robotics, proximal	30	2	Fixed	MD	4.28 [-1.06 9.63]	0.12	0	Nonsignificant	-	-	1	-	-	-	-	-	-	1	-
	Motor control	FMA-WH score	Type of robot device, joints targeted	Wrist/hand robotics, distal	30	2	Fixed	MD	1.60[-2.30 5.50]	0.42	0	Nonsignificant	-	-	1	-	-	-	-	-	-	1	-
	Upper-limb capacity	AMAT, ARAT, BBT, WMFT	Type of robot device	Hand robotics	39	2	Fixed	SMD	0.19 [-0.45 0.82]	0.56	0	Nonsignificant	1	1	-	-	-	-	-	-	-	-	-
	Motor control	FMA-arm score	Exoskeleton versus end-effector robotics	Exoskeleton	214	10	Fixed	MD	1.76 [-0.29 3.82]	0.09	22	Nonsignificant	1	6	-	-	-	-	-	-	-	2	1
	Motor control	FMA-WH score	Exoskeleton versus end-effector robotics, joints targeted	Exoskeleton, distal	31	2	Random	MD	-3.54 [-9.78 2.70]	0.27	73	Nonsignificant	-	2	-	-	-	-	-	-	-	-	-
	Muscle strength	MI (arm subscale), MPS, MRC	Exoskeleton versus end-effector robotics	Exoskeleton	88	3	Fixed	SMD	-0.14 [-0.57 0.29]	0.53	22	Nonsignificant	1	-	-	-	-	-	-	-	-	2	-
	Muscle tone	AS, MAS	Exoskeleton versus end-effector robotics	Exoskeleton	88	3	Fixed	SMD	0.42 [-0.01 0.84]	0.06	0	Nonsignificant	1	-	-	-	-	-	-	-	-	2	-
	Upper-limb capacity	AMAT, ARAT, BBT, WMFT	Exoskeleton versus end-effector robotics	Exoskeleton	130	4	Fixed	SMD	0.18 [-0.16 0.53]	0.3	0	Nonsignificant	1	3	-	-	-	-	-	-	-	-	-
	Motor control	FMA-arm score	Exoskeleton versus end-effector robotics	End-effector	670	24	Fixed	MD	2.59 [0.77 4.41]	0.005	35	Weak	5	3	-	1	-	-	1	1	1	12	-
	Motor control	FMA-SEC score	Exoskeleton versus end-effector robotics, joints targeted	End-effector, proximal	369	17	Fixed	MD	2.62 [1.48 3.76]	< 0.00001	34	Weak	2	2	1	1	-	-	1	-	3	7	-
	Motor control	FMA-WH score	Type of robot device, joints targeted, joints targeted	End-effector, distal	412	19	Random	MD	1.17 [0.31 3.23]	0.02	74	Weak	2	1	1	1	-	-	1	-	3	10	-
	Muscle strength	MI (arm subscale), MPS, MRC	Type of robot device	End-effector	406	17	Random	SMD	0.20 [-0.14 0.54]	0.24	57	Nonsignificant	3	1	-	-	-	-	1	1	1	10	-
	Muscle tone	AS, MAS	Type of robot device	End-effector	341	15	Fixed	SMD	0.19 [-0.03 0.41]	0.09	33	Nonsignificant	4	1	-	-	-	-	1	-	1	8	-
	Upper-limb capacity	AMAT, ARAT, BBT, WMFT	Type of robot device	End-effector	552	21	Fixed	SMD	0.00 [-0.17 0.17]	0.99	6	Nonsignificant	4	5	1	2	-	-	-	1	-	8	-
	ADL	BI, FIM, mRS	Type of robot device	End-effector	427	17	Random	SMD	0.27 [-0.05 0.59]	0.09	56	Nonsignificant	4	-	-	1	-	-	-	2	3	7	-
	Motor control	FMA-arm score	Time of RT-UL delivery	< 3 months	360	11	Random	MD	3.13 [-2.39 8.65]	0.27	62	Nonsignificant	4	1	-	-	-	-	1	1	1	2	1
	Motor control	FMA-SEC score	Time of RT-UL delivery, joints targeted	< 3 months, proximal	251	10	Fixed	MD	2.81 [1.44 4.17]	< 0.0001	44	Weak	2	-	-	-	-	-	1	-	3	4	-
	Motor control	FMA-WH score	Period of onset, joints targeted	< 3 months, distal	251	10	Random	MD	2.53 [0.46 4.60]	0.02	85	Weak	2	3	-	-	-	-	1	-	-	4	-
	Muscle strength	MI (arm subscale), MPS, MRC	Period of onset	< 3 months	346	12	Random	SMD	0.21 [-0.23 0.64]	0.35	69	Nonsignificant	3	-	-	-	-	-	1	1	1	6	-
	Muscle tone	AS, MAS	Period of onset	< 3 months	299	12	Fixed	SMD	0.21 [-0.03 0.45]	0.08	47	Nonsignificant	4	-	-	-	-	-	1	-	1	6	-
	Upper-limb capacity	AMAT, ARAT, BBT, WMFT	Period of onset	< 3 months	195	7	Fixed	SMD	-0.00 [-0.29 0.29]	0.99	40	Nonsignificant	4	-	-	-	-	-	-	-	-	3	-
	ADL	BI, FIM, mRS	Period of onset	< 3 months	331	13	Random	SMD	0.22 [-0.20 0.65]	0.3	67	Nonsignificant	3	-	-	-	-	-	-	1	3	6	-
	Motor control	FMA-arm score	Period of onset	> 3 months	506	23	Fixed	MD	1.52 [-0.04 3.09]	0.06	0	Nonsignificant	2	7	-	1	-	-	-	-	-	12	1
	Motor control	FMA-SEC score	Period of onset, joints targeted	> 3 months, proximal	118	7	Fixed	MD	2.17 [0.09 4.25]	0.04	23	Weak	2	-	1	1	-	-	-	-	-	3	-
	Motor control	FMA-WH score	Period of onset, joints targeted	> 3 months, distal	192	11	Fixed	MD	-0.19 [-1.65 1.27]	0.8	27	Nonsignificant	-	4	1	1	-	-	-	-	1	4	-
	Muscle strength	MI (arm subscale), MPS, MRC	Period of onset	> 3 months	148	8	Fixed	SMD	-0.04 [-0.37 0.29]	0.82	0	Nonsignificant	1	1	-	-	-	-	-	-	-	6	-
	Muscle tone	AS, MAS	Period of onset	> 3 months	130	6	Fixed	SMD	0.29 [-0.06 0.65]	0.1	0	Nonsignificant	1	1	-	-	-	-	-	-	-	4	-
	Upper-limb capacity	AMAT, ARAT, BBT, WMFT	Period of onset	> 3 months	487	17	Fixed	SMD	0.05 [-0.13 0.23]	0.58	0	Nonsignificant	2	6	1	2	-	-	-	1	-	5	-
	ADL	BI, FIM, mRS	Period of onset	> 3 months	96	4	Fixed	SMD	0.32 [-0.08 0.72]	0.12	0	Nonsignificant	1	-	-	1	-	-	-	1	-	1	-
	Motor control	FMA-arm score	Therapy dose	Dose-matched trials	808	32	Fixed	MD	2.28 [0.89 3.68]	0.001	33	Weak	6	9	-	1	-	-	1	-	-	14	1
	Motor control	FMA-SEC score	Therapy dose, joints targeted	Dose-matched trials, proximal	263	14	Fixed	MD	2.73 [1.24 4.21]	0.0003	40	Weak	2	2	1	1	-	-	1	-	-	7	-
	Motor control	FMA-WH score	Intervention, joints targeted	Dose-matched trials, distal	337	18	Random	MD	1.33 [-0.83 3.50]	0.23	75	Nonsignificant	2	3	1	1	-	-	1	1	-	9	-
	Muscle strength	MI (arm subscale), MPS, MRC	Intervention	Dose-matched trials	418	18	Random	SMD	0.12 [-0.20 0.43]	0.47	50	Nonsignificant	4	1	-	-	-	-	1	-	-	12	-
	Muscle tone	AS, MAS	Intervention	Dose-matched trials	399	17	Fixed	SMD	0.26 [0.05 0.47]	0.01	28	Weak	5	1	-	-	-	-	1	-	-	10	-
	Upper-limb capacity	AMAT, ARAT, BBT, WMFT	Intervention	Dose-matched trials	660	23	Fixed	SMD	0.06 [-0.09 0.22]	0.43	0	Nonsignificant	6	6	1	2	-	-	-	-	-	8	-
	ADL	BI, FIM, mRS	Intervention	Dose-matched trials	279	12	Fixed	SMD	0.08 [-0.16 0.33]	0.51	32	Nonsignificant	5	-	-	1	-	-	-	-	-	6	-
	Motor control	FMA-arm score	Intervention	Non-dose-matched trials	76	2	Fixed	MD	1.07 [-5.35 7.49]	0.74	0	Nonsignificant	1	-	-	-	-	-	-	1	-	-	-
	Motor control	FMA-SEC score	Intervention, joints targeted	Non-dose-matched trials, proximal	50	2	Fixed	MD	6.45 [0.92 11.98]	0.02	0	Weak	-	-	-	-	-	-	-	-	2	-	-
	Motor control	FMA-WH score	Intervention, joints targeted	Non-dose-matched trials, distal	106	3	Fixed	MD	0.96 [0.61 1.31]	< 0.00001	0	Weak	1	-	-	-	-	-	-	-	2	-	-
	Muscle strength	MI (arm subscale), MPS, MRC	Intervention	Non-dose-matched trials	76	2	Random	SMD	0.73 [-0.07 1.54]	0.07	59	Nonsignificant	1	-	-	-	-	-	-	1	-	-	-
	ADL	BI, FIM, mRS	Intervention	Non-dose-matched trials	148	5	Random	SMD	0.85 [0.32 1.38]	0.002	54	Weak	-	-	-	-	-	-	-	2	3	-	-
Zhang (2017) [33]	Motor control	FMA-UE	-	-	426	13	Random	SMD	0.56 [0.19 0.93]	0.003	68	Weak	4	4	-	-	-	-	-	-	4	1	-
	Motor control	FMA-UE	RT intervention	Additional RT (RT + CT vs CT (additional))	135	4	Random	SMD	0.71 [0.36 1.06]	< 0.0001	0	Weak	-	-	-	-	-	-	-	-	4	-	-
	Motor control	FMA-UE	RT intervention	Substitutional (RT vs CT)	291	9	Random	SMD	0.48 [-0.05 1.02]	0.08	78	Nonsignificant	4	4	-	-	-	-	-	-	-	1	-
	Motor control	FMA-UE	Period of onset	Acute/subacute	228	7	Random	SMD	0.38 [-0.11 0.86]	0.13	67	Nonsignificant	2	-	-	-	-	-	-	-	4	1	-
	Motor control	FMA-UE	Period of onset	Chronic	198	6	Random	SMD	0.82 [0.20 1.43]	0.01	74	Weak	2	4	-	-	-	-	-	-	-	-	-
Norouzi (2012) [34]	Motor control	FMA-UE, Chedoke-Mcmaster measure	Dose	Same duration/ Intensity therapy	204	6	Random	SMD	0.17 [-0.14 0.48]	0.28	13	Nonsignificant	-	3	-	-	-	-	-	-	-	3	-
	Motor control	FMA-UE, Chedoke-Mcmaster measure	Dose, phase	Same duration/ Intensity therapy, Chronic	169	4	Random	SMD	0.23 [-0.19 0.64]	0.28	35	Nonsignificant	-	3	-	-	-	-	-	-	-	1	-
	Motor control	FMA-UE, Chedoke-Mcmaster measure	Dose, Follow up	Additonal RT, 6 months follow up	160	4	Random	SMD	0.26 [-0.06 0.57]	0.11	0	Nonsignificant	-	2	-	-	-	-	-	-	-	2	-
	Motor control	FMA-UE, Chedoke-Mcmaster measure	Dose	Additonal RT	158	4	Random	SMD	0.46 [0.14 0.78]	0.004	0	Weak	-	-	-	-	-	-	-	1	2	1	-
	ADL	FIM	Dose	Additonal RT	106	3	Random	SMD	0.81 [-0.07 1.70]	0.07	77	Nonsignificant	-	-	-	-	-	-	-	1	2	-	-
	Motor control	FMA-UE, Chedoke-Mcmaster measure	Dose, phase	Additonal RT, Acute/Subacute	106	3	Random	SMD	0.48 [0.09 0.87]	0.01	0	Weak	-	-	-	-	-	-	-	1	2	-	-
	Motor control	Motor status scale	Dose	Additonal RT	76	2	Random	SMD	5.20 [1.71 8.70]	0.004	65	Weak	-	-	-	-	-	-	-	1	1	-	-

		Muscle strength	MPS	Dose. MPS score	Additonal RT, MPS (out of 20)	76	2	Random	SMD	2.31 [1.53 3.09]	<0.001	0	Weak	-	-	-	-	-	-	-	1	1	-	-
		ADL	FIM	Dose	Same duration/ Intensity therapy	62	3	Random	SMD	-0.00 [-0.62 0.61]	0.99	30	Nonsignificant	-	1	-	-	-	-	-	-	-	2	-
		Muscle strength	MPS	Dose. MPS score	Same duration/ Intensity therapy, MPS (out of 70)	56	3	Random	SMD	1.21 [-2.86 5.27]	0.56	0	Nonsignificant	-	1	-	-	-	-	-	-	-	2	-
		Motor control	FMA-UE, Chedoke-Mcmaster measure	Dose, phase	Additonal RT, Chronic	52	1	Random	SMD	0.42 [-0.13 0.97]	0.14	-	Nonsignificant	-	-	-	-	-	-	-	-	-	1	-
		Motor control	FMA-UE, Chedoke-Mcmaster measure	Dose, phase	Same duration/ Intensity therapy, Acute/Subacute	35	2	Random	SMD	0.08 [-0.61 0.77]	0.83	4	Nonsignificant	-	-	-	-	-	-	-	-	-	2	-
		Motor control	FMA-UE, Chedoke-Mcmaster measure	Dose, Follow up	Same duration/ Intensity therapy, 8 months follow up	30	1	Random	SMD	0.86 [0.10 1.61]	0.03	-	Weak	-	-	-	-	-	-	-	-	1	-	-
		Motor control	Motor status scale	Dose	Same duration/ Intensity therapy	20	1	Random	SMD	0.65 [-4.83 6.13]	0.82	-	Nonsignificant	-	-	-	-	-	-	-	-	-	1	-
	Saragih (2023) [54]	Motor control	FMA-UE	Period of onset	Acute	650	7	Random	SMD	-0.11 [-2.38 2.16]	0.93	0.21	Nonsignificant	4	1	-	-	-	-	-	-	-	2	-
		Pain	California functional evaluation, Douleur Neuropathique pain scale, pain scale, VAS	-	-	261	5	Random	SMD	-0.34 [-0.58 -0.09]	0.01	0	Weak	1	-	-	-	-	-	-	-	1	3	-
		Other measures	SIS, MMSE, FIM, Addenbrooke cognitive examination-revised, participants`cognitive function	-	-	143	5	Random	SMD	0.25 [-0.08 0.59]	0.13	0	Nonsignificant	3	-	-	-	-	-	-	-	-	2	-
		ADL	MAL	Amount of use	-	189	6	Random	WMD	0.12 [-0.10 0.35]	0.28	0	Nonsignificant	1	-	-	-	-	-	-	1	-	4	-
		ADL	MAL	Quality of movement	-	189	6	Random	WMD	0.15 [0.03 0.28]	0.71	0	Nonsignificant	1	-	-	-	-	-	-	1	-	4	-
	Lo (2017) [55]	Motor control	FMA-UE, MI	-	-	860	29	Random	SMD	0.07 [-0.11 0.26]	0.45	41	Nonsignificant	9	8	-	-	-	-	-	-	-	12	-
		Motor control	FMA-UE, MI	Dose	TR: 0	507	16	Random	SMD	0.13 [-0.13 0.39]	0.33	48	Nonsignificant	3	8	-	-	-	-	-	-	-	5	-
		Motor control	FMA-UE, MI	Duration	Follow-up at less than or equal to 3 months	293	9	Random	SMD	0.06 [-0.22 0.33]	0.68	24	Nonsignificant	5	3	-	-	-	-	-	-	-	1	-
		Motor control	FMA-UE, MI	Duration	Follow-up at more than 3 months	217	7	Random	SMD	0.00 [-0.45 0.45]	1	59	Nonsignificant	2	2	-	-	-	-	-	-	-	3	-
Neurological disease	Ferreira (2021) [75]	Comprehensive measure	Life Habits, SIS	Dose	RT vs minimum intervention	52	1	-	SMD	0.60 [0.10, 1.20]	0.025	-	Weak	-	-	-	-	-	-	-	-	-	1	-
		Comprehensive measure	Life Habits, SIS	Dose	RT vs OI	301	6	Fixed	SMD	0.20 [-0.00, 0.40]	0.085	0	Nonsignificant	1	2	-	-	-	-	-	-	-	3	-
		Comprehensive measure	Life Habits, SIS	Dose, bias, duration	RT add vs OI, excluding low-quality studies, short term	492	6	Fixed	SMD	-0.60 [-1.10 -0.10]	0.022	69.9	Weak	3	2	-	-	-	-	-	-	-	1	-
		Comprehensive measure	Life Habits, SIS	Dose, bias, duration	RT add vs OI, excluding low-quality studies, medium term	50	2	Fixed	SMD	0.20 [-0.30 0.80]	0.405	0	Nonsignificant	1	1	-	-	-	-	-	-	-	-	-
		Comprehensive measure	Life Habits, SIS	Dose, duration	RT vs minimum intervention, short term	52	1	-	SMD	0.60 [0.10 1.20]	0.025	-	Weak	-	-	-	-	-	-	-	-	-	1	-
		Comprehensive measure	Life Habits, SIS	Dose, duration	RT vs OI, short term	286	5	Fixed	SMD	0.20 [0.00 0.40]	0.155	0	Nonsignificant	1	2	-	-	-	-	-	-	-	2	-
		Comprehensive measure	Life Habits, SIS	Dose, duration	RT add vs OI, short term	459	4	Random	SMD	-0.50 [-1.10 -0.10]	0.13	33.78	Nonsignificant	2	1	-	-	-	-	-	-	-	1	-
		Comprehensive measure	Life Habits, SIS	Dose, duration	RT add vs OI, medium term	50	2	Fixed	SMD	0.20 [-0.30 0.80]	0.405	0	Nonsignificant	1	1	-	-	-	-	-	-	-	-	-

Abbreviations: ABILHAND, A Measure of Manual Ability for People with Upper Limb Impairment; ACTIVLIM, Activity Limitations for Patients with Upper and/or Lower Limb Impairments; AMAT, Arm Motor Ability Test; ARAT, Action Research Arm Test; AS, Ashworth Scale; BBT, Box and Block Test; BI, Barthel Index; CAHAI, Chedoke Arm and Hand Activity Inventory; CI, confidence interval; CMSA, Chedoke-McMaster Stroke Assessment; EMG, electromyography; FIM, Functional Independence Measure; FMA, Fugl-Meyer Assessment; FMA-SE, Fugl-Meyer Assessment-Shoulder and Elbow; FMA-UE, Fugl-Meyer Assessment-Upper Extremity; FMA-WH, Fugl-Meyer Assessment-Wrist and Hand; FAT, Frenchay Arm Test; GRASSP, Graded Redefined Assessment of Strength, Sensibility and Prehension; K-SDQ, Korean version of the Shoulder Disability Questionnaire; MAL, Motor Activity Log; MAL-QOM, the quality of 124 movement section of the Motor Activity Log ; MAS, Modified Ashworth Scale; MBI, Modified Barthel Index; MCS, Mental Composite Score; MD, mean difference; MI, Motricity Index; MMT, Manual Muscle Testing; MPS, Motor Power Scale; MRC, Medical Research Council; mRS, modified Rankin Scale; MSS, Motor Status Scale; NHPT, Nine-Hole Peg Test; NRS, Numerical rating scale; PCS, Physical Composite Score; QUEST, Quality of Upper Extremity Skills Test; QuickDASH, Quick version of the Disabilities of the Arm, Shoulder, and Hand questionnaire; pROM, passive range of motion; SF-36, Short-Form 36; SIS, Stroke Impact Scale; SMD, standardised mean difference; UPDRS, Unified Parkinson's Disease Rating Scale; VAS, Visual Analog Scale; WMD, weighted mean difference; WMFT, Wolf Motor Function Test

Classification of dose matching:

A: Robot-assisted training in addition to conventional therapy was compared with dose-matched conventional therapy.

B: Robot-assisted training was compared with dose-matched conventional therapy.

C: Robot-assisted training was compared with dose matched training consisting of robot-assisted training and conventional therapy.

D: Robot-assisted training was compared with dose-matched other intervention (e.g. body weight support training or elgometer).

E: Robot-assisted training was compared with dose-matched other robot-assisted training.

F: Robot-assisted training was compared with dose-matched training using the same robot on other training mode.

G: Robot-assisted training with other interventions was compared with dose-matched conventional therapy.

H: Robot-assisted training in addition to conventional therapy was compared with conventional therapy. Conventional therapy in both groups was the same amount.

I: Robot-assisted training was compared with minimal or no conventional therapy including sham.

J: Comparisons between more than three groups that included the robot assisted training.
